# Deuteration of Six‐Membered N‐Heteroarenes: Chemistry and Applications

**DOI:** 10.1002/chem.202502659

**Published:** 2025-10-27

**Authors:** Liju Raju, Davide Benedetto Tiz, Adrian Iles, Janez Košmrlj, Ross D. Jansen‐van Vuuren

**Affiliations:** ^1^ Faculty of Chemistry and Chemical Technology University of Ljubljana Večna pot 113 Ljubljana 1000 Slovenia; ^2^ Department of Chemistry Queen's University Chernoff Hall, 90 Bader Lane Ontario K7L 3N6 Canada

**Keywords:** deuteration, deuterium, heteroaromatics, pyrazine, pyridazine, pyridine, pyrimidine, regioselectivity

## Abstract

Six‐membered N‐heteroarenes (“6NHetAr”) play pivotal roles in the pharmaceutical, agrochemical, and materials science industries. In recent times, deuterium (D)‐labelled 6NHetAr have demonstrated enhanced performance over their unlabelled counterparts. For example, VV116 and VX‐984, approved therapeutics for the treatment of COVID‐19 and cancer, and deuterated functional materials for use as ligands in catalysts and organometallic light‐emitters. As interest in this field continues to grow, there is a corresponding need to develop more efficient, scalable, and sustainable approaches to deuterate 6NHetAr. In this work, we comprehensively review approaches developed for the incorporation of D into 6NHetAr, their fused counterparts, and complex compounds containing 6NHetAr. We also provide an overview of established and emerging applications for these materials.

## Graphical abstract

Deuterated six‐membered N‐Heteroarenes (6NHetAr) are finding utility in an increasing repertoire of applications. Our paper comprehensively reviews synthetic approaches that have been reported for the deuteration of 6NHetAr and their fused counterparts while providing an overview of established and emerging applications for these materials.

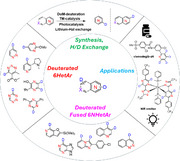

**Abbreviations**
6NHetAr: 6‐membered N‐Heteroarenes
CMD: concerted metalation deprotonation
DG: Directing Group
DMG: Directed Metalating Group
D*m*M: Directed *meta*‐Metalation
D*o*M: Directed *ortho* Metalation
EQE: External quantum efficiency
ETL: Electron‐transporting layer
FG: Functional Group
HIE: Hydrogen Isotope Exchange
H/D: Hydrogen/Deuterium
ISC: Intersystem crossing
KIE: Kinetic Isotope Effect
LDA: lithium diisopropylamide
LL‐RTP: Long‐Lived Room Temperature Phosphorescence
LiTMP: lithium tetramethylpiperidide
*n*‐BuLi: *n*‐Butyllithium
NaBD_4_
: Sodium borodeuteride
NIR: Near Infrared
OLED: Organic light emitting diode
pK_a_
: The negative logarithm of an acid dissociation constant (K_a_)
SET: Single Electron Transfer (process)
TM: Transition Metal
TMS: Trimethylsilyl

## 1 Introduction

### 1.1 Six‐membered N‐Heteroaromatic Compounds (6NHetAr)

**Figure 1:**
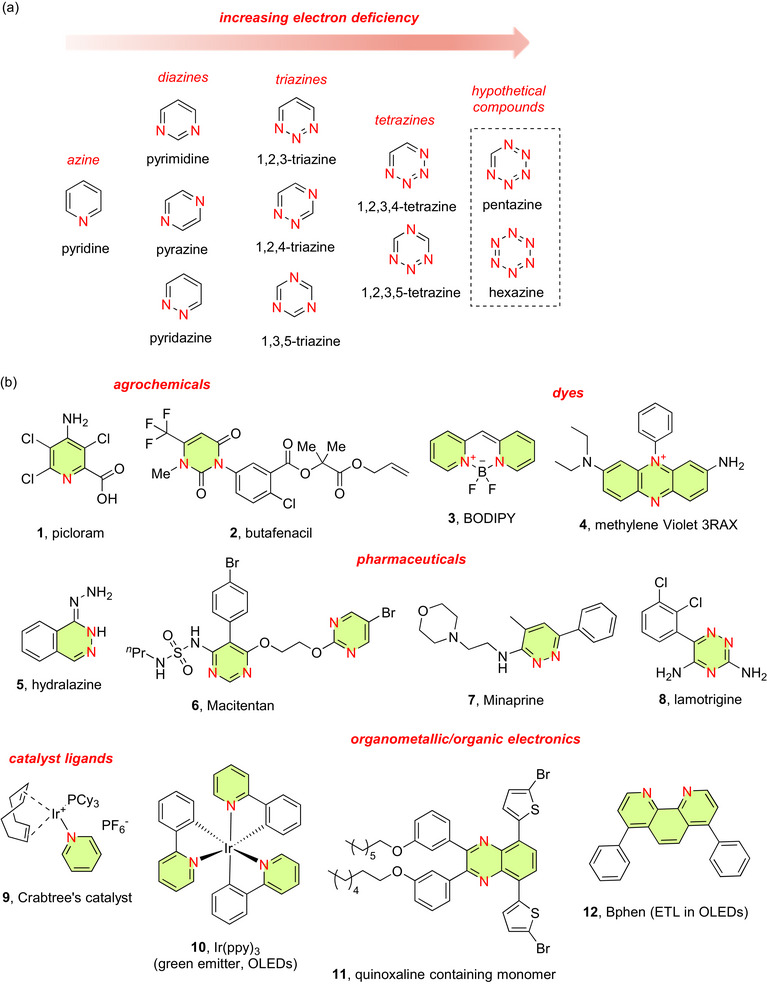
a) Chart showing progressively increased electron deficiency with additional Ns present in the rings of basic 6NHetAr compounds, b) Examples of compounds containing 6NHetAr (**1**–**12**) used in a wide range of applications.

6‐Membered N‐heteroarenes (abbreviated 6NHetAr) are distinctly electron deficient, becoming increasingly electron deficient with each additional N added to the ring. Therefore, the order of increasing electron deficiency is pyridine < pyrimidine < triazine < tetrazine (Figure 1a). The theoretical compounds pentazine and hexazine, also shown, would have even larger electron deficiencies if they could be prepared and stabilized.^[^ ^1^ ^]^

This electronic character plays an important role in their functions and applications. For example, their electron‐deficient nature allows for interactions with electron‐rich species, leading to diverse applications in areas like bio‐orthogonal chemistry, material science, and catalysis (they play a critical role as ligands in metal complexes).^[^ ^2^, ^3^, ^4^, ^5^ ^]^ 6NHetAr and their fused derivatives are therefore important components of many agrochemicals,^[^ ^6^, ^7^ ^]^ dyes,^[^ ^8^, ^9^ ^]^ pharmaceuticals^[^ ^10^, ^11^, ^12^, ^13^, ^14^, ^15^ ^]^ metal catalysts (as stabilizing ligands), and organic/organometallic electronic materials (Figure 1b). Examples of significant compounds containing 6NHetAr components include the pesticide picloram (**1**),^[^ ^16^ ^]^ the herbicide butafenacil (**2**),^[^ ^17^ ^]^ dyes such as the BODIPY analogue (**3**),^[^ ^18^ ^]^ and methylene violet 3RAX (**4**),^[^ ^19^ ^]^ the hypertension drugs hydralazine (Apresoline) (**5**)^[^ ^20^ ^]^ and macitentan (Opsumit®) (**6**),^[^ ^21^ ^]^ the antidepression agent minaprine (**7**),^[^ ^22^ ^]^ and the anti‐epilepsy drug lamotrigine (**8**),^[^ ^23^ ^]^ and Crabtree's catalyst (**9**),^[^ ^24^ ^]^ a homogeneous catalyst for hydrogentation/deuteration and hydrogen transfer reactions.^[^ ^25^ ^]^ Additionally, tris(2‐phenylpyridine)iridium(III), Ir(ppy)_3_) (**10**) is used in organic light‐emitting diodes (OLEDs),^[^ ^26^ ^]^ a thiophene‐quinoxaline‐thiophene monomer, **11**, used to prepare polymers for Organic Photovoltaics,^[^ ^27^ ^]^ while the emissive material 4,7‐diphenyl‐1,10‐phenanthroline (**12**, Bphen) is widely used as an electron‐transporting layer (ETL) in OLEDs.^[^ ^28^ ^]^

### 1.2 Deuteration of Organic Compounds

In reactions involving C─H bond cleavage, replacement of C─H by slightly stronger C─D bond(s) can result in considerable changes in the rate of reaction as D has a mass twice that of H, giving the C─D bond a reduced vibrational stretching frequency compared to the C─H bond, and thus a lower zero‐point energy.^[^ ^29^ ^]^ Thus, the replacement of a H atom with D at a metabolic “hot spot” can enable modulation of the metabolism of a drug^[^ ^13^, ^29^, ^30^, ^31^, ^32^ ^]^ and its binding energetics with receptors^[^ ^33^ ^]^ or nucleic acids.^[^ ^34^, ^35^ ^]^ A prominent example of D‐inhibited metabolism is found in deutetrabenazine (Austedo, **13a**), the deuterated analogue of tetrabenazine (**13b**) and the first deuterated drug to be formally approved for commercial usage in 2017 by the U.S. FDA.^[^ ^36^ ^]^ Although both **13a** and **13b** are useful for treating chorea associated with Huntington's disease and tardive dyskinesia, by replacing the methoxy groups with deuteromethoxy groups (i.e., in **13b**), the drug's metabolism is inhibited, improving the drug bioavailability and lowering the dosage requirement.^[^ ^31^ ^]^

Since the approval of deutetrabenazine, several drugs containing deuteration at an N‐heteroaromatic portion have been approved by various regulatory bodies, including VX‐984 (**14**), vismodegib‐*d*_4_ (**15**), and VV116 (**16**). Many more are currently in clinical trials^[^ ^40^ ^],^ e.g., JNJ38877605‐d_1_ (**17**), an anticancer drug in which D incorporation limits liver toxicity.^[^ ^41^ ^]^

Strategically exchanging C─H for C─D in 6NHetAr has also improved the stability and enhanced select properties of functional materials such as light emissive molecules, e.g., Near Infrared (NIR) light emitter **18**,^[^ ^38^ ^]^ and metal complex ligands such as 1,10‐phenanthroline‐2,3,5,6,8,9‐*d*_6_, 4,7‐di(phenyl‐*d*_5_) (**19**).^[^ ^42^ ^]^

Deuterated compounds are also valuable as internal standards for bioanalytical studies in drug development.^[^ ^43^, ^44^ ^]^ For instance, deuterated pyridine and pyrimidine internal standards have allowed for alternative approaches to diagnose disorders based on differential pyridine/pyrimidine metabolism.^[^ ^45^, ^46^ ^]^

**Figure 2:**
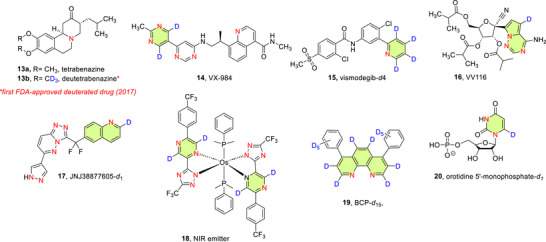
Chemical structures of deutetrabenazine (**13a**) and its nonlabelled counterpart **13b**, VX‐984 (**14**), vismodegib‐*d4* (**15**), VV116 (**16**), Anticancer drug (**17**),^[^ ^37^ ^]^ NIR emitter (**18**),^[^ ^38^ ^]^ 1,10‐Phenanthroline‐2,3,5,6,8,9‐d6, 4,7‐di(phenyl‐d5 (**19**) and C‐5 deuterated orotidine 5′‐monophosphate (**20**).^[^ ^39^ ^]^

Additionally, the kinetic isotope effect (KIE) is exploited in elucidating enzymatic and chemical reaction mechanisms. For example, deuterated pyridines and pyrimidines find extensive utility in the elucidation of enzymatic mechanisms.^[^ ^47^, ^48^, ^49^, ^50^, ^51^ ^]^ A particularly illustrative example is found in the study of orotidine 5′‐monophosphate (OMP) decarboxylase^[^ ^39^ ^]^ in which deuteration of OMP at the C‐5 position of the pyrimidine OMP‐d_1_, **20** (Figure 2) permits measurement of the secondary H/D KIE at this position for the enzyme‐catalyzed decarboxylation.^[^ ^52^ ^]^ This mechanistic insight has subsequently contributed to the development of a small‐molecule inhibitor of OMP decarboxylase that has garnered interest as a potential anticancer agent.^[^ ^53^ ^]^

### 1.3 Synthesis of Deuterated N‐Heteroaromatics

In this paper, we review approaches to the synthesis of deuterated 6NHetAr. Other reviews by Atzrodt et al., ^[^ ^54^ ^]^ Kopf et al., ^[^ ^40^ ^]^ Jiang et al.,^[^ ^55^ ^]^ Li et al., ^[^ ^56^ ^]^ and Prakash et al.^[^ ^30^ ^]^ are rather more general, covering deuteration techniques used across a broad spectrum of organic compounds (aromatic, aliphatic, and heterocyclic). Additional reviews by Yang and Hesk,^[^ ^57^ ^]^ Valero and Derdau,^[^ ^58^ ^]^ and Reid^[^ ^59^ ^]^ outline the current state of transition‐metal (TM)‐catalyzed deuteration of sp^2^ (and sp^3^) C─H bonds, also covering a range of substrates.

Our review is unique in that it focuses on 6NHetAr ‐ namely azines, diazines, triazines, and tetrazines (and their fused derivatives), providing a comprehensive analysis of the different synthetic approaches taken to deuterate these electron‐deficient substrates at the various positions on their rings (mono‐selectively as well as in more than one position, e.g., di‐ / tri‐ / per‐deuteration etc.).

Reviews focused on deuteration of 6NHetAr include an older review by Turck et al. in 2001, ^[^ ^60^ ^]^ *only* considering metalation‐deuteration of pyridines, pyrimidines, pyrazines, pyridazines, and quinolines, with more recent reviews (2014) by Quéguiner and Mongin^[^ ^61^, ^62^ ^]^ and Gros,^[^ ^63^ ^]^ highlighting advances in the deuteration of pyridine and some of the diazines via directed *ortho*‐metalation (D*o*M). These reviews focus on metalation of some 6NHetAr, and, in all cases, deuteration is not the focus of the review.

Yang and Hesk^[^ ^57^ ^]^ also recently reviewed the literature related to HIE chemistry used to prepare poly‐deuterated pyridines and diazines using Fe and Ni catalysts, while Ila et al.^[^ ^64^ ^]^ and Alonso et al.^[^ ^65^ ^]^ have provided valuable reviews on the functionalization of heteroaromatics and aromatics via dehalogenative methods, with some examples of deuterated pyrimidines. Lim et al.^[^ ^66^ ^]^ recently provided a review covering transition metal‐catalyzed multideuteration of N‐heteroarenes. Pieters et al.^[^ ^67^ ^]^ also recently published an account on catalyzed HIE reactions using Ru, Ru/C, and Ir catalysts and/or nanoparticles (NPs). These methods enable deuteration of pyridine and other *N*‐heterocycles, drugs, and biological molecules. A review by Modak and Maiti^[^ ^68^ ^]^ covers metal catalyzed defunctionalisation reactions of arenes, including examples of diazines. Lastly, in 2004, Yamamoto^[^ ^69^ ^]^ provided a short overview of deutero‐dechlorination of diazines.

Since our paper would be the first review encompassing deuteration of *all* 6NHetAr (and their fused derivatives), we have not placed any date restrictions on the work. Our hope is for this to be a comprehensive guide and useful resource for practitioners of isotope, medicinal, and materials chemistry, and providing the earliest results would give context to the development of certain transformations (in some cases, no further work has been done to advance early results, so their unearthing is useful and interesting). We first discuss the deuteration of pyridine and its derivatives before reviewing chemistry used for the synthesis of 6NHetAr with increasing number of Ns in the N‐heteroaromatic ring (diazines, triazines, and tetrazines). We then review protocols reported which enable the deuteration of compounds containing more than one 6NHetAr, followed by single processes used for the deuteration of several types of 6NHetAr, and conclude our paper by highlighting the reported applications of deuterated 6NHetAr.

## 2 Deuteration of 6NHetAr

### 2.1 Pyridines

**Figure 3:**
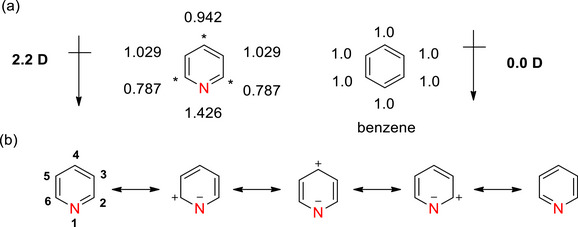
a) Dipole moment and π‐electron densities of pyridine and benzene. The asterisks indicate the electron‐deficient positions *α*‐ and *γ*‐ relative to the ring nitrogen atom (modified from^[^ ^70^ ^]^). b) Limiting resonance structures and numbering of pyridine.

Compared with benzene, the electrons in the pyridine ring are polarized toward the more electronegative nitrogen atom through inductive and resonance effects, introducing a permanent dipole (Figure 3a). This polarization causes partial positive charges on the *α*‐ and *γ*‐positions of the pyridine ring as they possess *π*‐electron densities < 1. Pyridine is therefore a *π*‐deficient heterocyclic system that can be represented with resonance structures as shown in Figure 3b.^[^ ^70^ ^]^

**Scheme 1:**
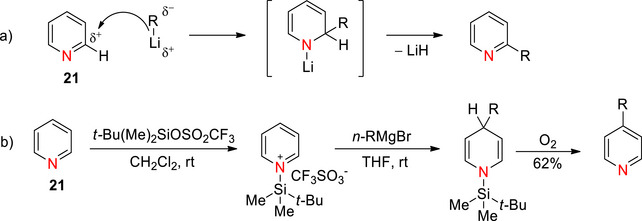
a) General C‐2 nucleophilic addition‐elimination of pyridine.^[^ ^10^ ^]^ b) C‐4 nucleophilic addition‐elimination of pyridine.^[^ ^73^ ^]^

The electron‐rich nitrogen atom acts as the weakly basic (p*K*_aH_ ∼5.2)^[^ ^71^ ^]^ and nucleophilic center of pyridine. Typical reactions of pyridine include C‐2(C‐6)‐ and C‐4‐nucleophilic substitution/additions that are enabled by mesomeric stabilization as the developing negative charge can be delocalized onto the nitrogen atom. For example, pyridine reacts with alkyl or aryl lithium species through nucleophilic addition at C‐2 followed by elimination of lithium hydride, producing 2‐alkyl(aryl)pyridines (Scheme 1a).^[^ ^72^ ^]^ The C‐4 nucleophilic substitutions on pyridine can be achieved using Grignard reagents, for example 4‐substituted *N*‐tri‐*iso*‐propylsilylpyridinium salts react with hindered dialkylmagnesiums at C‐4, providing a route through to 4,4‐dialkylpiperidines (Scheme 1b).^[^ ^73^ ^]^

Harsh conditions can also be used to elicit S_E_Ar reactions at C‐3. These include nitration and sulphonation. Furthermore, the presence of additional substituents on the ring can influence relative patterns of reactivity through steric and/or *ortho*/*para*‐ and *meta*‐directing effects.^[^ ^10^, ^11^, ^71^, ^74^ ^]^

There are three main approaches that have been taken to deuterate pyridines.

#### 2.1.1 Lithiation‐deuteration

**Scheme 2:**
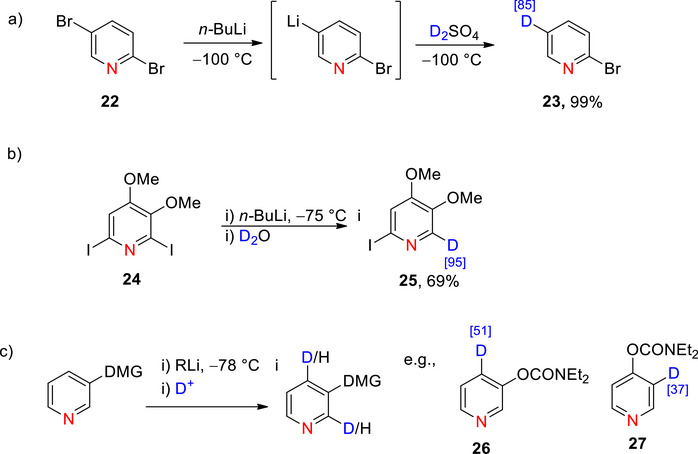
a) Lithium‐halogen exchange of 2,5‐dibromopyridine **22**.^[^ ^81^ ^]^ (NB: % D incorporation is reported in blue square parentheses, here and throughout the manuscript). b) Lithium‐iodine exchange in a substituted pyridine **24** by Mongin et al.^[^ ^82^ ^]^ c) General deuteration of a DMG‐functionalized pyridine via D*o*M with an early example from Miah and Snieckus, resulting in the formation of **26** and **27**.^[^ ^83^ ^]^

Within the use of lithiation for incorporating D within aromatic/heteroaromatic ring systems, there are two strategies that can be used: lithium‐halogen exchange and D*o*M. Independently discovered in 1938 by Gilman^[^ ^75^ ^]^ and Wittig,^[^ ^76^ ^]^ lithium‐halogen exchange has been utilized for aromatic/heteroaromatic hydrodehalogenation and deuterodehalogenation.^[^ ^65^, ^77^, ^78^, ^79^, ^80^ ^]^ In general, low temperatures (−60 to −120 °C) are required for these reactions to avoid nucleophilic addition and minimize side reactions caused by the low functional group (FG) tolerance of the highly reactive alkyllithium reagents.^[^ ^10^ ^]^ An early example of deuterodebromination was demonstrated by *ipso*‐substitution at C‐5 of 2,5‐dibromopyridine (**22**) in 1977 (Scheme 2a).^[^ ^81^ ^]^ In this case, extreme conditions were employed: react with *n*‐BuLi at −100 °C followed by quench with deuterated acid (D_2_SO_4_) to provide 2‐bromo‐5‐deuteropyridine (**2**) in quantitative yield and 85% D incorporation via the lithiated derivative. This result was unexpected since C‐2 should be able to stabilize the negative charge of the aryl lithium better through induction and resonance (Fig. 3b). However, C‐5 seems to be more sterically accessible.

Examples of lithium‐halogen exchange: iodine−lithium exchange in a substituted pyridine **24** (100% regioselective at C2) followed by deuterodeiodination to form **25**.^[^ ^82^ ^]^ Deuteration by D*o*M proceeds via directed metalation group (DMG)‐coordinated *ortho*‐deprotonation, followed by quenching of the reactive carbanion with an electrophilic D source, e.g., CH_3_OD, CD_3_OD, D_2_O. Effective DMGs contain Lewis basic heteroatoms enabling coordination with Lewis acidic lithium cations and are sterically hindered and/or charge deactivated, reducing the likelihood of attack by nucleophilic lithium bases., e.g., ─OCONR_2_, ─CONR_2_,^[^ ^84^ ^]^ ─OP(O)(NEt_2_)_2_.^[^ ^85^ ^]^ Both alkyllithiums and lithium amides can be utilized for D*o*M;^[^ ^60^, ^61^, ^62^, ^63^ ^]^ however, to avoid undesired nucleophilic additions, sterically hindered bases are typically used to lithiate pyridine and other *π*‐deficient ring systems.

This is exemplified by the H/D exchange of functionalized pyridines **26** and **27** with 51 and 37% D incorporation, respectively (Scheme 2c).^[^ ^60^, ^61^, ^62^, ^63^, ^83^, ^84^, ^85^ ^]^ Examples of other DMGs that have been employed to initiate H/D exchange in pyridines are shown in Table 1. Snieckus and Tsukazaki^[^ ^86^ ^]^ reported an *O*‐carbamate directed deuteration at the C‐2 of a 4‐trimethylsilyl substituted pyridine, with moderate (58%) D incorporation (Table 1, Entry 1). Additionally, Mongin et al.^[^ ^87^ ^]^ lithiated unprotected carboxylic acid‐substituted pyridines. For example, deuteration of 5‐bromo‐nicotinic acid was achieved using 2 eq. of lithium tetramethylpiperidide (LiTMP) at ─50 °C followed by a D_2_O quench and acidic work‐up, affording 58% yield with 99% D incorporation (Table 1, Entry 2).

**Table 1:** Monodeuteration of pyridine via DoM.

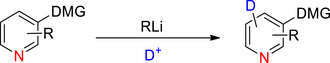						
Entry	DMG	Position(s) of H/D	R	Conditions	%Yield (% D)	Refs.
1	3‐OCONEt_2_	C2	4‐TMS	LiTMP, THF, CD_3_OD, −78 °C	87 (58)	[86]
2	3‐CO_2_H	C4	5‐Br	LiTMP, THF, D_2_O, −50 °C	58 (99)	[87]
3	3‐Br	C4	6‐Cl	*t*‐BuLi, THF, D_2_O, −78 °C	95 (98)	[88]
4	3,5‐Cl_2_	C4	‐	*n*‐BuLi, THF, D_2_O, −75 °C	n.a.^*^ (>99)	[89]
5	3,5‐Cl_2_	C4	‐	LiTMP, THF, D_2_O, −75 °C	n.a.^*^(70)	[89]

* n.a. = not available.

In 2004, Pierrat et al.^[^ ^88^ ^]^ achieved excellent (98%) D incorporation at C‐4 of 3‐bromo‐6‐chloropyridine using *t*‐BuLi (Table 1, Entry 3). This result is particularly interesting given the greater directing ability of Cl compared with Br; metalation at C‐4 is favored by resonance stabilization of the developing negative charge. Shortly thereafter, Quéguiner and Mongin^[^ ^89^ ^]^ employed a di‐DMG framework of 3,5‐dichloropyridine for deuteration at the 4‐position using *n*‐BuLi. The *meta*‐substituted Cl atoms cooperatively direct *ortho*‐lithiation at C‐4 resulting in > 99% D incorporation (Table 1, Entry 4). When LiTMP was used in place of *n*‐BuLi, only 70% D incorporation was achieved, likely due to steric hindrance caused by the bulky piperidine (Table 1, Entry 5).

Gros et al.^[^ ^90^ ^]^ demonstrated direct lithiation at the 6‐position of 2‐chloro and 2‐methoxypyridine using *n*‐BuLi‐LiDMAE (Table 2, Entry 1–2), rather than DMG directed *ortho*‐lithiation with LiTMP (Table 2, Entry 3–4). *n*‐BuLi‐LiDMAE allowed for regioselective lithiation at the position adjacent to the nitrogen through formation of lithium aggregates which complex with the nitrogen at that position.

**Table 2:** Monodeuteration of pyridine via direct lithiation and DoM.

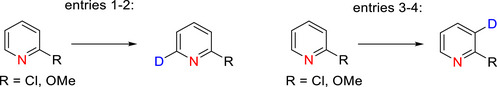						
Entry	DMG	Position(s) of H/D	R	Conditions	%Yield (% D)	Refs.
1	‐	C6	2‐Cl	*n*‐BuLi‐LiDMAE, Hexane, DCl/D_2_O, −78 °C	65 (≤98)	[90]
2	‐	C6	2‐OMe		68 (≤98)	
3	2‐Cl	C3	‐	LiTMP, THF, DCl/D_2_O, −78 °C	70 (≤98)	
4	2‐OMe	C3	‐		92 (≤98)	

#### 2.1.2 Metal‐Catalyzed Deutero‐Defunctionalisation

Metal‐catalyzed defunctionalisation of arenes^[^ ^68^ ^]^ was extended to deutero‐defunctionalisation in heteroaromatic compounds, including pyridines, although there are fewer reported reactions than for aromatics. For example, pyridine derivatives have only been explored in decarboxylation, deborylation, dephosphoniation, and dehalogenation reactions. Compared with other methods of deuteration, deutero‐defunctionalisation is typically regiospecific and boasts high levels of D‐incorporation (Tables 3 and 4) and high FG tolerance.^[^ ^68^, ^91^, ^92^ ^]^

**Table 3:** Monodeuteration of pyridine via deutero‐defunctionalisation.

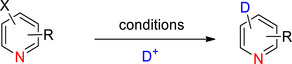					
Entry	X	R	Conditions	%Yield (% D)	Refs.
1	4‐CO_2_H	2‐F	Ag_2_CO_3_, DMSO, D_2_O, 120 °C	81 (95)	[93]
2	3‐ CO_2_H	2‐Cl	Ag_2_CO_3_, DMSO, D_2_O, 120 °C	100 (95)	[93]
3	2‐CO_2_H	3‐CO_2_H	Ag_2_CO_3_, DMSO, D_2_O, 120 °C	91 (95)	[93]
4	3‐B(OH)_2_	2‐OMe, 6‐Cl	PPh_3_AuNTf_2_, THF, D_2_O, 90 °C	58 (95)	[94]
5	4‐Bpin	2,6‐Cl_2_	[Ir(OMe)(COD)]_2_, THF, D_2_O, 80 °C	82 (96)	[95]
6	4‐PPh_3_^+^OTf^−^	2‐pyridyl	K_2_CO_3_, CD_3_OD, D_2_O, rt	87 (89)	[96]

**Table 4:** Monodeuteration of pyridine via deuterodehalogenation.

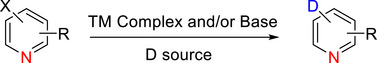					
Entry	X(D)	R	Conditions	%Yield (% D)	Refs.
1	3‐I	2‐NMeBn	KOMe, Me_3_SiSiMe_3_, CD_3_CN, 25 °C	83 (94)	[99]
2	5‐Br	2‐(2,5‐Me_2_‐pyrrole), 3‐(2‐benzoxazole)	Pd(dba)_2_, K_2_CO_3_, S‐Phos, D_8_‐IPA, MeCN, 100 °C	68 (≥95)	[100]
3	3‐Br	2‐CN	Pd(dba)_2_, *t*‐Bu_3_P, DCOONa, DMSO, 80 °C	80 (>98)	[92]
4	3‐Br	5‐[4‐(2‐pyrimidinyl)‐1‐piperazinylmethyl]	Pd(PPh_3_)_4_, NaO*^t^* Bu, D_8_‐IPA, *hv*, rt	n.a. (65)	[101]
5	2‐Br	3‐CO_2_H	Cu NWAs cathode, K_2_CO_3_, MeCN, D_2_O, rt	67 (95)	[91]
6	5‐Cl	2‐NBn_2_	[Pd(allyl)Cl]_2_, NHC ligand, Cs_2_CO_3_, toluene, α‐D‐benzhydrol, 90 °C	92 (>99)	[104]
7	2‐Cl	‐	Pd NSs/KPCN‐2, D_2_O, *hv*	>98 (>98)	[106, 109]
8	2,4‐Cl_2_	3‐COOEt	Pd/C, D_2_, K_2_CO_3_, EtOD, rt	62 (n.a.)	[106]
9	4‐F	2,3,5,6‐F_4_	CdSe, *hv*, Na_2_SO_3_, MeCN, D_2_O	99 (n.a.)	[107]
10	2‐Cl	4‐COOMe	PC1, (MeS)_2_ HCO_2_Na, DMSO:D_2_O, r.t.*hv*,	72 (99)	[108]
11	2‐Cl	5‐COOEt	PC1, (MeS)_2_ HCO_2_Na, DMSO:D_2_O, r.t.*hv*,	77 (92)	[108]

* n.a. = not available.

There is only one report detailing deutero‐decarboxylation of pyridines: Grainger et al.^[^ ^93^ ^]^ employed Ag_2_CO_3_‐catalyzed deutero‐decarboxylation with three substituted pyridines: 2‐chloronicotinic acid, 3‐fluoroisonicotinic acid, and quinolinic acid, providing access to 2‐D‐nicotinic acid, a form of vitamin B3 (Table 3, entries 1–3). The *α*‐heteroatom is a prerequisite for heteroaromatic decarboxylation, stabilizing the generated negative charge. The presence of electron withdrawing substituents on the ring facilitates decarboxylation for the same reason.

**Scheme 3:**
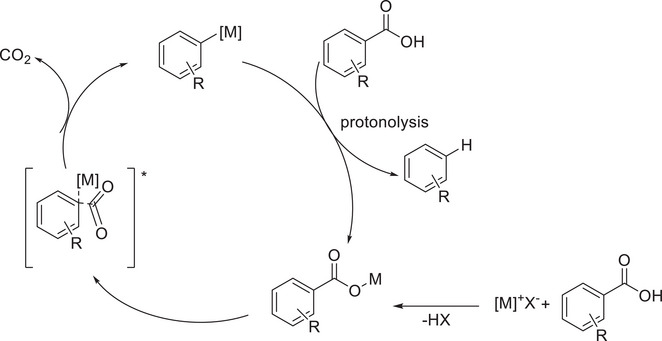
Proposed mechanism of decarboxylative deuteration of aromatic carboxylic acids using copper or silver catalysts according to Rudzki et al.^[^ ^97^ ^]^ Reproduced with permission.

Around the same time that this work was published, Rudzki et al.^[^ ^97^ ^]^ reported the selective decarboxylative deuteration of aromatic carboxylic acids using copper and silver catalysts. Both Rudzki and Grainger^[^ ^93^ ^]^ exploit the concept of decarboxylative deuteration, but the exchange involves different mechanistic sequences. In the work of Rudzki et al., copper‐ and silver‐catalyzed decarboxylations proceed through formation of an aryl–metal intermediate after CO_2_ extrusion from the carboxylate (scheme 3). This intermediate undergoes protonolysis or deuterolysis to give the corresponding arene, with copper being more effective for meta/para‐substituted substrates and silver for *ortho*‐substituted systems. Their study also highlighted mechanistic diversity, since some heteroaromatic acids could undergo decarboxylation thermally without metal catalysis, and scrambling of deuterium was sometimes observed (e.g., in indoles). By contrast, Grainger et al.^[^ ^93^ ^]^ developed a streamlined silver carbonate–catalyzed protocol in DMSO/D_2_O under similarly harsh conditions (120 °C) that also proceeds via an aryl–Ag(I) intermediate but delivers exceptional regioselectivity: deuterium is incorporated exclusively at the *ipso*‐position of the former carboxyl group with no evidence of scrambling and in consistently high yields with excellent levels of D incorporation.

A few years later, Barker and colleagues^[^ ^94^ ^]^ employed a gold catalyst (PPh_3_AuNTf_2_) to deutero‐deborylate heteroaryl and electron‐rich arylboronic acid substrates, complementing previous deuterodecarboxylation methods which are limited to electron‐withdrawing aryls. For example, (6‐chloro‐2‐methoxypyridin‐3‐yl)boronic acid (Table 3, Entry 4). The cooperative directing effects of the *meta*‐related chlorine and methoxy groups enabled a high level of D incorporation as this creates an electron‐rich heteroarylboronic acid system. Furthermore, this chemistry can be carried out in “green” solvents such as dimethyl carbonate.

Similarly, Kallepalli et al.^[^ ^95^ ^]^ demonstrated that deutero‐deborylation of several type of boronate esters containing electron rich and electron deficient substituents, for example, 4‐Bpin‐2,6‐dichloropyridine affords 2,6‐dichloropyridine‐d_1_ in 82% yield and with 96% D incorporation using an [Ir(OMe)(COD)]_2_ catalyst in THF/D_2_O (Table 3, Entry 5).

**Scheme 4:**
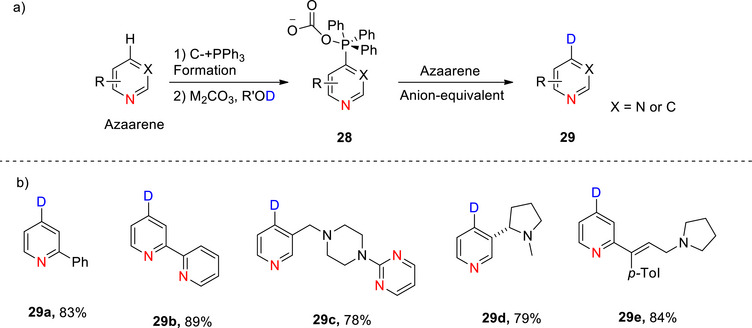
a) Hydrogen isotope exchange via heterocyclic phosphonium salts, from Koniarczyk et al. via an intermediate **28** to form deuterated pyridines **29**.^[^ ^96^ ^]^ b) Examples of deuteration using this strategy, forming compounds **29a–29e**. Regrettably, the authors did not report % D incorporation for any of the compounds.

Using a different approach, Koniarczyk et al.^[^ ^96^ ^]^ generated phosphonium salts at the 4‐position on the pyridine ring of a range of substituted pyridines, other aza‐arenes (e.g., pyrazine, azaindole), and drug‐like compounds. The salt is formed using Tf_2_O, PPh_3_, and NEt_3_ or DBU, and acts as a carbanion for *ipso*‐selective deuteration (Scheme 4a). For example, deuteration was achieved at the 4‐position of the azaarene 2‐(2‐pyridyl)pyridine (**28**) in high yield and D incorporation to produce 2‐(2‐pyridyl)pyridine‐d_1_, **29b** (Table 3, Entry 6). This was achieved using a mixture of CD_3_OD and D_2_O as the D source, with the release of CO_2_ and Ph_3_PO serving as the driving force for the reaction. Other deuterated pyridine‐containing compounds are shown in Scheme 4b.

Concerning deuterodehalogenation, previous reviews focused on dehalogenation transformations of heteroaromatics and aromatics^[^ ^65^, ^98^ ^]^ are worth delving into to understand the chemistry. Deutero‐dehalogenative procedures have been developed to access deuterated pyridines (Table 4), detailed as follows.

An interesting KOMe/disilane‐mediated dehalogenation was reported by Liu et al.^[^ ^99^ ^]^ for various (hetero)aromatics. For example, *N*‐methylbenzyl‐2‐pyridinamine was deuterated at the 3‐position with 94% D incorporation and 83% yield (Table 4, Entry 1). This study showed *ipso*‐selectivity providing precise access to libraries of deuterated indoles and quinolines and other pyridine‐based complex substrates. Pd‐catalyzed deutero‐debromination of more complex pyridines by Donald^[^ ^100^ ^]^ and Liang^[^ ^101^ ^]^ were both achieved using isopropanol‐d_8_ as the D source in varying yields and D incorporation (Table 4, Entry 2 and 4).

Another example of a Pd‐catalyzed deutero‐debromination^[^ ^92^ ^]^ was performed with great FG tolerance and high yield on a cyano‐substituted pyridine (Table 4, Entry 3). Liu et al.^[^ ^91^ ^]^ developed an approach to synthesis deuterated nicotinic acid derivatives (nicotinic‐2‐d_1_, nicotinic‐5‐d_1_, and nicotinic‐6‐d_1_ acids) involving the deutero‐debromination of corresponding bromonicotinic acids using a Cu nanowire array‐based cathode and D_2_O. For example nicotinic acid‐2‐d_1_ was obtained with an isolated yield of 67% and 95% D incorporation (Table 4, Entry 5). Although this yield was lower than that of Grainger and colleagues,^[^ ^93^ ^]^ Zhang's method also produced nicotinic‐5‐d_1_ and nicotinic‐6‐d_1_ acids in high yields.

Three Pd‐catalyzed deutero‐dechlorination methods provided excellent D‐incorporation on a range of pyridines (Table 4, Entry 6–8).^[^ ^102^, ^103^, ^104^ ^]^

Another approach to deuterodehalogenation involves the use of photocatalysis.^[^ ^105^ ^]^ Several authors report novel approaches. For example, a novel Pd nanosheet photocatalyst was used by Ling et al.^[^ ^106^ ^]^ to provide, e.g., 2‐deuteropyridine from the chloro‐derivative in near quantitative yield and D‐incorporation (Table 4, Entry 8). Another photocatalytic method by Liu et al.^[^ ^107^ ^]^ deuterated the 4‐position of pentafluoropyridine using a newly developed CdSe photocatalyst (Table 4, Entry 9). These results are remarkable, given the strength of the C–F bond and the lack of similar deutero‐defluorination methods. Importantly, this deutero‐dehalogenation permits regiospecific D‐incorporation while displaying broad FG tolerance and the CdSe catalyst is recyclable, maintaining a yield of >90% after 3 runs.

Li et al.^[^ ^108^ ^]^ reported the organophotocatalytic deuterdehalogenation of (hetero)aryl and alkyl chlorides, including pyridine derivatives, using visible light, and D_2_O as the deuterium source. The system combines an aryl‐amine photocatalyst (e.g., tris(4′‐(dimethylamino)phenyl)benzene, PC1) with an organic disulfide cocatalyst (e.g., dimethyl disulfide) under blue LED irradiation in DMSO/D_2_O, with sodium formate as a sacrificial electron and hydrogen donor. The reaction was carried out at room temperature in air and tolerates a wide range of (hetero)aryl, for example, deuterated heterocycles including pyridines with electron‐donating substituent or electron‐withdrawing substituent (Table 4, Entry 10 and 11) The photocatalyst, upon visible‐light excitation, acts as a strongly reducing photoredox catalyst that transfers a single electron to the pyridyl chloride, generating a pyridyl radical after C─Cl bond cleavage. At the same time, the excited photocatalyst can induce homolytic cleavage of the disulfide cocatalyst to form thiyl radicals. Sodium formate serves as both electron donor (closing the photocatalytic cycle) and hydrogen/deuterium shuttle. The thiyl radical abstracts deuterium from D_2_O and delivers it to the pyridyl radical, yielding the deuterated pyridine. This dual role of the photocatalyst, as both a SET reductant and an energy transfer sensitizer, is crucial for efficient C─Cl activation and high % D incorporation.

#### 2.1.3 Transition Metal (TM) or / Base / Organo‐ Catalyzed HIE

Incorporation of D within pyridines (as mono‐, di‐, and poly‐deuteration) can also be performed using metal‐catalyzed hydrogen isotope exchange (HIE) chemistry (Tables 5, 6, 7).

**Table 5:** Monodeuteration of pyridine via HIE.

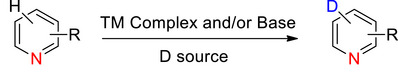					
Entry	Position(s) of H/D	R	Conditions	% Yield, (% D)	Refs.
1	C3	2‐CO_2_H	[Rh(Cp*)(MeCN)_3_](SbF_6_)_2_, NaOAc, D_2_O, 90 °C	n.a. (< 4)	[111]
2	C4	2,6‐Me_2_	[(PC_NHC_P)Fe(H)_2_N_2_)], C_6_D_6_, N_2_, 50–80 °C	n.a. (98)	[112]
3	C4	2‐CN, 3‐F	Ag_2_CO_3_, S‐Phos, K_2_CO_3_, Toluene, 90 °C	53 (97)	[113]
4	C5	2‐Ph	Pd(TFA)_2_, SPO, Na_2_CO_3_, HFIP, D_2_O, 100 °C, 2 h	91 (94)	[114]

* n.a. = not available.

##### Monodeuteration

Selective mono‐deuteration in pyridines is typically achieved through homogeneous transition metal (TM) catalysis,^[^ ^58^ ^]^ with deuteration usually occurring *ortho* to a directing group (DG), although it is possible for deuteration to occur at positions *meta* or *para* to a DG.^[^ ^110^ ^]^ Garreau et al.^[^ ^111^ ^]^ have shown selective *ortho*‐deuteration of 2‐pyridinecarboxylic acid using Rh(III) metal, although with low % D incorporation (Table 5, Entry 1). Similarly, 3‐ and 4‐pyridinecarboxylic acids could be *ortho*‐and *meta* deuterated, albeit with no more than 4% D incorporation in either case.

In many cases, HIE is favourable at positions *ortho* to a heteroatom due to precoordination of the metal center with the heteroatom. However, HIE at the 4‐position is also favourable when the 2‐ and 6‐position are sterically hindered due to mesomeric carbanion stabilization at those positions^[^ ^112^ ^]^ (Table 5, Entry 2) or additional directing group effects (Table 5, Entry 3).

For example, Hu et al.^[^ ^113^ ^]^ reported 53% D incorporation at the 4‐position of a 2‐cyano‐3‐fluoropyridine using an Ag_2_CO_3_/SPhos catalyst (Table 5, Entry 3). In this case, additive directing effects of electron‐withdrawing and donating groups positioned *ortho* to one another appear to dictate the observed regiospecificity. Zheng et al.^[^ ^114^ ^]^ reported Pd‐catalyzed regioselective H/D exchange at the α‐position of pyridines by employing a secondary phosphine oxide (SPO) ligand as an internal base, Na_2_CO_3_ as an external base, and D_2_O as source of D. A wide range of pyridine (and quinoline)‐containing substrates were deuterated, with electron‐rich and ‐deficient functionalization, with D incorporation ranging from 15–98%, e.g., pyridine (Table 5, entry 4). The mechanism of action is dependent on the tautomerization of the SPO ligand, favouring deprotonation (via abstraction of the H form the C─H bond by the anionic oxygen), followed by H/D exchange based on a concerted metalation deprotonation (CMD) pathway.^[^ ^115^ ^]^

Wang et al.^[^ ^116^ ^]^ reported an efficient method of highly selective deuteration of pyridine using easily available barium oxide as a catalyst and D_2_ as a D source. Distinctive from the reported KOtBu‐promoted β‐ and γ‐regioselective D/H exchange of pyridines, the D incorporation catalyzed by BaO occurs at the α‐position of pyridine. Eisele et al.^[^ ^117^ ^]^ reported the deuteration of the pyridine containing fungicide boscalid with 50% yield using a RuCl_2_(PPh_3_)_3_ catalyst and KOD/D_2_O. The procedure enabled H/D exchange at the 4 position of the pyridine unit in moderate D incorporation (33%).

##### Dideuteration

Coordination of metal centers to the aromatic ring N facilitates many high yielding routes that provide access to pyridines that are deuterated in high % D at the 2‐ and 6‐ positions (Table 6). Although heterogeneous catalysis tends to be less site‐selective,^[^ ^54^ ^]^ *ortho*‐deuteration was achieved early on by Rubottom et al.^[^ ^118^ ^]^ using 5% Ru/C (Table 6, entry 1) and later by Alexakis et al.^[^ ^119^ ^]^ using Rh black catalysis (Table 6, entry 2). The DG of these substrates overpowered the affinity of the metal for the 2‐ and 6‐positions, selecting for the 2‐ and 4‐ positions instead. Regrettably, the researchers did not attempt to reuse the filtered catalyst in subsequent reactions runs, although this sustainable approach might be coveted in the current era.^[^ ^120^ ^]^

**Table 6:** Dideuteration of pyridine via HIE.

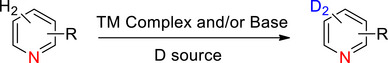					
Entry	Position(s) of H/D	R	Conditions	%Yield (% D)	Refs.
1	C2, C6	4‐NMe_2_	Ru/C, CD_3_OD, D_2_, rt	>90 (n.a.)	[118]
2	C2, C6	4‐COCH_3_	Rh black, THF, D_2_, rt	n.a. (99)	[119]
3	C2, C6	‐	Zn, D_2_SO_4_/D_2_O, 90 °C	n.a. (95)	[122]
4	C2, C6	‐	Ru_3_(CO)_12_, *t*‐BuOD, 115 °C	n.a. (79)	[123]
5	C2, C6	1‐O, 4‐Me	[(*t*‐BuP, NMe)Ir(COD)]BArF_24_, C_6_H_5_Cl, D_2_, rt, 2 h	89 (45)	[124]
6	C2, C6	1‐O, 4‐Ph	KO*^t^* Bu (1 equiv.), DMSO‐d_6_ (0.5 M), 20 °C, rt, 5 min	95 (98)	[125]
7	C2	1‐Me	Atomic C, then CH_3_OD	n.a. (99)	[126]
8	C3	1‐Me	Atomic C + CH_3_OD	n.a. (99)	[126]
9	C2, C6	1‐NBz^†^	CuBr (10 mol%), K_2_CO_3_ (2 equiv.), PhCl, CD_3_OD, 125 °C, 16 h	n.a. (92)	[127]
10	C2, C6	3‐Me	RuNP@PVP catalyst, THF, D_2_, rt	n.a. (>80)	[128]
11	C2, C6	N/A (pyridine)	Pd(TFA)_2_, secondary phosphine oxide ligand, Na_2_CO_3_, HFIP, D_2_O, 100 ^o^C	87 (75)	[114]

* n.a. = not available. ^†^Bz = benzoyl.

**Scheme 5:**
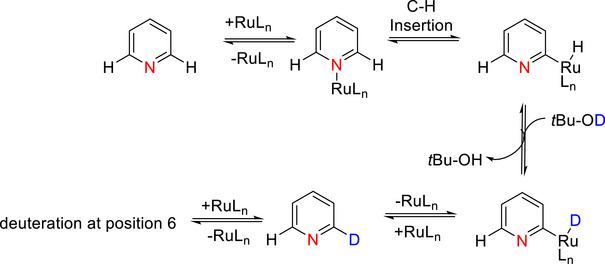
Mechanism of dideuteration of pyridines according to Gröll et al.^[^ ^123^ ^]^ Adapted with permission. Copyright 2025 American Chemical Society.

Huber,^[^ ^121^ ^]^ Perrin,^[^ ^122^ ^]^ and Gröll^[^ ^123^ ^]^ achieved dideuteration at the 2,6‐positions of pyridine in high yields with moderate (>79%) D incorporation using Zn (excess Zn dust in D_2_SO_4_/D_2_O at 90 °C) (Huber and Perrin) and Ru [Ru_3_(CO)_12_] catalysis (Gröll) (Table 6, entry 3 and 4, respectively). The mechanism of action of the Ru catalysis involved metal insertion at the closest C−H bond of the substrate, before hydrogen is exchanged for deuterium originating from the solvent (eventually via reversible HD formation and alkoxide coordination). Finally, reductive elimination leads to the deuterated target compound (Scheme 5).

Valero et al.^[^ ^124^ ^]^ achieved good yields (> 70%) but relatively low (< 50%) D incorporation at the 2‐position by reacting simple pyridine *N*‐oxides with an Ir catalyst (Tamm catalyst) and D_2_. Only two substrates were reported, one being 4‐methyl‐pyridine *N*‐oxide (Table 6, Entry 5). The low % D incorporation may be due to poor binding by the heterocyclic oxides to the cationic iridium(I) complex, i.e., the C─H activation step, the rate‐determining step.^[^ ^129^ ^]^

**Scheme 6:**
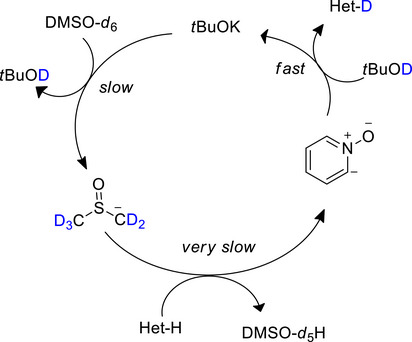
Mechanism of D*o*M‐deuteration according to Montoli et al.^[^ ^125^ ^]^ Reproduced under the terms of the Creative Commons CC BY license,.

Montoli et al.^[^ ^125^ ^]^ reported D*o*M‐deuteration on a large scope (>34 substrates, including pharma compounds) of pyridine *N*‐oxides with the N‐oxide acting as a DMG, e.g., (Table 6, entry 6). Here, the proposed mechanism implied a strong dependence on the initial abstraction of a D from DMSO‐d_6_ by KO*t*Bu, forming *t*BuOD alongside the dimsyl anion, which deprotonates the pyridinium *N*‐oxide at the most acidic (*ortho*)‐positions. The heterocyclic oxide can then be quantitively deuterated by *t*BuOD, providing the desired product and regenerating fresh KO*t*Bu (Scheme 6).

This also explains why DMSO‐d_6_ is the most suitable solvent: its corresponding anion is basic enough to deprotonate the heterocyclic oxide, unlike other solvents such as acetone‐d_6_ / MeCN‐d_3_. An older (1997) study by Pan & Shevlin^[^ ^126^ ^]^ showed that *ortho*‐deuterated pyridinium ylides could be generated by reaction of *N*‐methylpyrrole with atomic C, to generate *N*‐methyl‐3‐dehydropyridinium ylide, which can trap D at either the 2‐ or the 3‐position, depending on when the D source is added (Table 6, entries 7 and 8). Similarly, Mousseau et al.^[^ ^127^ ^]^ were able to synthesize *ortho*‐deuterated pyridinium ylides using CuBr / K_2_CO_3_ in CD_3_OD (Table 6, entry 9).

Rousseau et al.^[^ ^128^ ^]^ demonstrated deuteration of several substituted pyridines (including 3‐methyl pyridine, Table 6, entry 10) at the 2‐ and 6‐positions in high % D by using D_2_ and ruthenium‐basedNPs on a polyvinylpyrrolidone support (PVP). Notably, two nicotinic acetylcholine receptor agonists were deuterated regioselectively on the pyridine component with a high level of deuterium incorporation. The mechanism of deuteration involves initial coordination of the Ns with the Ru catalysts, enabling the H/D exchange process to follow. Zheng et al.^[^ ^114^ ^]^ reported a Pd‐catalyzed regioselective H/D exchange at the α‐position of pyridines has been achieved by employing secondary phosphine oxide as an internal base Table 6, entry 11) the mechanism has been discussed in **section 2.1.3.1**.

Rayner et al.^[^ ^103^ ^]^ reported the dideuteration of pyridine via deutero‐dechlorination of several dichloronicotinate derivatives using K_2_CO_3_, 5% Pd/C catalyst, and D_2_ gas, with moderate yields % D for all compounds (% D incorporation was not reported for these dideuterated nicotinates).

##### Polydeuteration

In 1973, Fischer et al.^[^ ^130^ ^]^ were the first to demonstrate the perdeuteration of pyridine using D_2_O and platinized short fibre asbestos. In this case, the reaction mixture was heated to 170 °C for 24 h to obtain perdeuterated pyridine with 90% yield and 92.5% D incorporation.

In 1989, Werstiuk and Ju^[^ ^131^ ^]^ reported poly‐deuteration of a range of pyridine derivatives by H/D exchange with D_2_O as D‐source at elevated temperatures. The deuterated compounds were generally prepared in good yields, with perdeuterated pyridine obtained in > 80% yield and 95% D incorporation (Table 7, Entry 1). In 1997, Kebede and Pavlik^[^ ^132^ ^]^ reported poly‐deuteration of all 6 dimethylpyridine isomers using D_2_O and K_2_CO_3_ at 180 °C for 5.5 days. D incorporation occurred at unsubstituted ring positions 2 and 6, and on methyl groups at the 2, 4, and 6 position. This demonstrates the inherent reactivity of pyridine caused by mesomeric stabilization of negative charge at the 2, 4, and 6 positions (see Figure 3). 2,5‐Dimethylpyridine was deuterated to produce 6‐deutero‐5‐methyl‐2‐trideuteromethylpyridine in 93% yield and > 95% D incorporation (Table 7, Entry 2).

**Table 7:** Polydeuteration of pyridines via HIE.

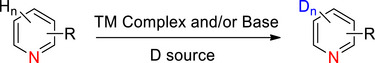					
Entry	Position(s) of H/D	R	Conditions	%Yield (% D)	Refs.
1	C2, C3, C4, C5, C6	‐	D_2_O, 300 °C, 62 h	>80 (95)	[131]
2	C6, C2─(H_3_)	2,5‐Me_2_	D_2_O, K_2_CO_3_, 180 °C, 132 h	93 (>95)	[132]
3	C3, C4, C5, C6, C3′, C4′, C5′, C6′*	2‐(2‐Py)	1 M NaOD/D_2_O, 200 °C	90 (>98)	[133]
4	C2, C5, C6‐	3‐CO_2_H	Pd/C, H_2_, D_2_O, 160 °C	98 (68)	[134]
5	C2, C3, C4, C5, C6	‐	Cp(IMes)RuH_3_, C_6_D_6_, 50 °C	n.a (91 (C4), 90 (C2, C3))	[136]
6	C2, C3, C4, C5, C6	‐	Ir‐H_4_ (Ir PCP Polyhydride complex), C_6_H_12_, D_2_O, 80 °C	n.a. (35)	[137]
7	C2, C3, C4, C5, C6─H_5_	‐	[Ir(COD)(OMe)]_2_, THF, D_2_, 55 °C	n.a. (78 (C2), 20 (C3), 44 (C4))	[13]
8	C3, C4, C5, C6	bisacodyl	[Ir(COD)(OMe)]_2_, THF, D_2_, 55 °C	n.a (total D: 2.79)	[12]
9	C4, 2,6‐(H_3_)_2_	2,6‐(CH_3_)_2_	(^Mes^CNC)Fe(CH_2_SiMe_3_)_2_(N_2_), H_2_, C_6_D_6_, 50 °C	n.a. (98)	[138]
10	C3, C5	2,6‐Cl_2_, 4‐Br	Ag_2_CO_3_/CyPh_2_P, toluene, K_2_CO_3_, D_2_O, 120 °C	90 (95)	[139]

* Primed positions refer to substituent pyridine. When different % D are present, the values are separated by a slash. n.a. = not available.

A range of thienyl‐pyridines and bipyridines were fully or partially deuterated by Browne et al.^[^ ^133^ ^]^ using either 10% Pd/C in D_2_O, pure D_2_O (without catalyst), or 1 M NaOD/D_2_O (all at 200 °C). Yields ranged from 70–95% with > 98% D in most cases, with 2,2′‐bipyridine‐d_8_ synthesized in 90% yield and > 98% D using the NaOD/D_2_O procedure (Table 7, Entry 3). Another route to deuterated nicotinic acid was reported by Esaki et al.^[^ ^134^ ^]^ in which heterogeneous catalysis using 10% Pd/C, H_2_, and D_2_O provided tri‐deuteration at the 2‐, 5‐, and 6‐positions (Table 7, Entry 4).

Shortly thereafter, Guy and Shapley^[^ ^135^ ^]^ determined the rates of H/D exchange at various positions of ─CH_3_, ─N(CH_3_)_2_, ─OCH_3_, ─CN, and ─CO_2_H ‐substituted pyridines using a Pd/PVP colloid catalyst in D_2_O. Deuteration was primarily observed at the α sites of the substituted pyridines, proposed to occur through an intermediate that forms, possessing a Pd─C(α) bond. The rate of H/D exchange at the various C─H bonds could be observed through truncated ^1^H NMR spectra.

Synthesis of perdeuterated pyridine was performed using various Ru and Ir catalysts, with a wide range of % D incorporation values reported (Table 7, Entry 5–7).^[^ ^13^, ^136^, ^137^ ^]^ The highest D incorporation (91%) was achieved by Mai et al.^[^ ^136^ ^]^ at the 4‐position of pyridine using an *N*‐heterocyclic carbene (NHC) supported Ru catalyst and benzene‐d_6_ in only 2 hours (Table 7, Entry 5). Otherwise, % D values ∼ 59–80% were observed for pyridines functionalized with methyl, halogens, acetyl, and other N‐heteroarenes. Daniel‐Bertrand et al.^[^ ^13^ ^]^ reported the perdeuteration of a range of pyridines (simple pyridines as well as those found in nicotine, brompheniramine, bisacodyl, and doxylamine, i.e., demonstrating good FG tolerance (bromide, alkyl amines, phenol esters)) using an air stable [Ir(COD)(OMe)]_2_ precatalyst in conjunction with D_2_ (also enabling tritiation with T_2_) at 55 °C. As an example, pyridine could be deuterated in various % D at the five C─H bonds (Table 7, Entry 7), while bisacodyl (Table 7, Entry 8) showed D incorporation at eight C─H bonds.

**Scheme 7:**
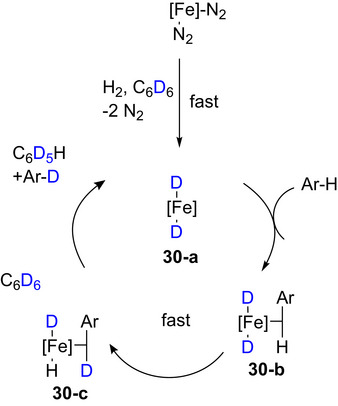
Proposed mechanisms for iron‐catalyzed HIE. Adapted with permission from ref. [138]. Copyright 2025 American Chemical Society.

A recent advance in Fe‐catalyzed HIE reported by the Corpas et al. (Chirik group)^[^ ^138^ ^]^ produced a range of deuterated electron‐rich and ‐poor (hetero)aromatics, including 2,6‐dimethylpyridine‐d_3_. Only the 4‐position of the ring and the methyl protons were deuterated due to the bulky nature of the Fe catalyst (Table 7, Entry 9). The mechanism depended on the presence of H_2_ in the reaction vessel: under H_2_ atmosphere an iron(II) dihydride intermediate is rapidly formed by addition of D_2_ to [Fe]─N_2_, which then rapidly transforms into an iron(II) dideuteride (**30‐a**) through exchange with benzene‐d_6_ as shown in Scheme 7. This iron dideuteride compound is responsible for C─H activation and coordination of an arene C─H bond forming **30‐b**. C─H activation leads to the formation of **30‐c** that is subsequently converted to Ar─D by arene exchange with benzene‐d_6_ and further H/D exchange.

Hu et al.^[^ ^139^ ^]^ investigated the poly‐deuteration of (hetero)aryl bromides using Ag_2_CO_3_/CyPh_2_P and D_2_O at 120 °C. A range of Me‐, MeO‐, Cl‐, and F_3_C‐substituted bromopyridines were deuterated in high yield and D‐incorporation. For example, 4‐Br‐2,6‐Cl_2_‐pyridine was deuterated at the 3,5‐positions in 95% D incorporation and 90% yield (Table 7, Entry 10). Eisele et al.^[^ ^117^ ^]^ also demonstrated the dideuteration of the pyridine containing fungicide boscalid with 40% yield using the RuCl_2_(PPh_3_)_3_ catalyst and KOD/D_2_O. The procedure enabled H/D exchange at the position 2,4, and 6 of the pyridine unit with D incorporation of 68%, 62% and 24% respectively.

**Scheme 8:**
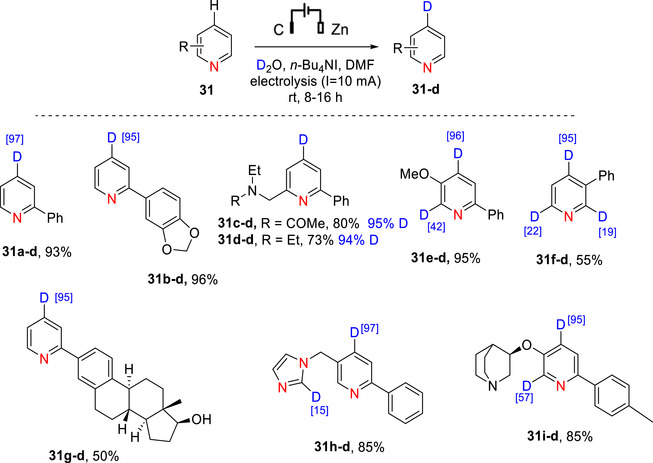
Electrocatalytic γ‐deuteration of functionalized pyridines **31** to form their deuterated counterparts **31‐d** (**31a–d** – **31i–d**).^[^ ^140^ ^]^

Electrochemistry has been employed for the γ‐deuteration of pyridines.^[^ ^140^ ^]^ The process involved an undivided cell containing D_2_O, *n*‐Bu_4_NI, and DMF under constant‐current electrolysis (10 mA), and employs a graphite anode and zinc cathode at room temperature. A large range of substituted and functionalized pyridines were deuterated in moderate‐good yields and mostly high (>80%) D incorporation. As an example, 2‐substituted pyridine‐d_1_ (**31**) was prepared in excellent % D (>95%) from the nondeuterated counterpart, for a range of functionalized pyridines (Scheme 8, 31). There were also some compounds with mixed results such as low deuteration at undesirable positions, e.g., 3‐phenylpyridine was deuterated at the *ortho*‐positions (in 19 and 22% D incorporation) as well as the γ‐position (95%) with a total incorporation of 1.36 deuterium atoms (compound **31f–d**).

**Scheme 9:**
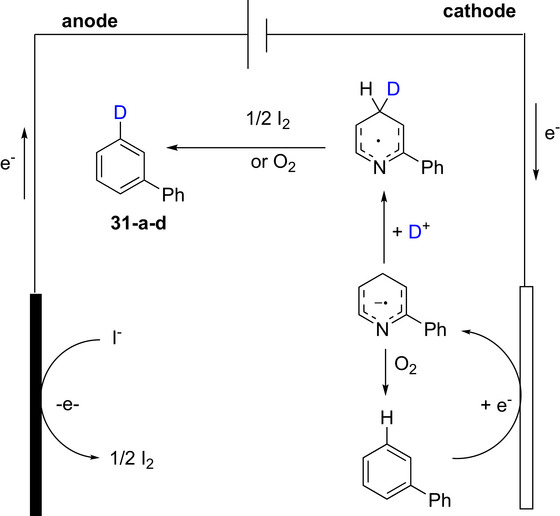
Proposed reaction mechanism for the electrochemical γ‐deuteration of pyridines (also see Scheme 8, above). Modified from (ref. [140]) Copyright 2025 American Chemical Society.

The process could also be used for late‐stage modification of bioactive pyridine‐containing compounds such as estradiol derivative **31e**, CYP11B1 inhibitor **31f**, and AWQ051 **31 g** (Scheme 4). The mechanism (exemplified using compound **31**, Scheme 9) was proposed to involve initial oxidation of the iodide ion from *n*‐Bu_4_NI at the anode to produce I_2_. Simultaneously, **31** is reduced at the cathode, forming the radical anion I, which undergoes electrophilic attack by D^+^ at the γ‐position to produce the stable radical intermediate II. Finally, intermediate II reacts with I_2_ or O_2_ (via oxidative elimination) to provide the desired deuterated product **31a–d**. Intermediate I can also be oxidized by O_2_ to yield starting the nondeuterated substrate (denoted **31**).

**Scheme 10:**
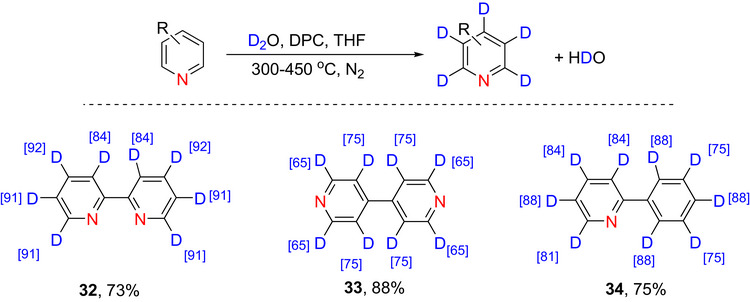
Perdeuteration of pyridines (**32**–**34**) using defective porous carbon reported by Gao et al.^[^ ^141^ ^]^

Finally, Gao et al.^[^ ^141^ ^]^ demonstrated deuteration of a range of pyridines in continuous flow chemistry systems using defective porous carbon (DPC) using D_2_O as the deuterium source. The reaction provided perdeuterated pyridine derivatives as shown in Scheme 10. The mechanism involves the adsorption of D_2_O within the carbon defects (especially hole‐type defects); the resulting OD species enable H/D exchange within the pyridines.

### 2.2 Fused Pyridines

The fused pyridines quinoline and isoquinoline are still relatively electron‐deficient within the pyridine portion, but less so overall because the fused benzene ring provides additional electron density. These compounds therefore demonstrate a type of “split personality” as the benzene part is still relatively electron‐rich while the pyridine component retains its electron deficiency.

In terms of acid‐base properties, compared with pyridine (p*K*_aH_ ≈ 5.2 in water), the fused pyridines are of similar basicity (quinoline p*K*_aH_ ≈ 4.94; isoquinoline ≈ 5.4). However, the extended conjugation which delocalizes the electron density over the fused system slightly lowers the availability of the N lone pair.

When fused with electron‐rich heteroaromatics such as furans, imidazoles, pyrroles, or thiophenes, the system becomes polarized: the heteroaryl ring is electron‐rich, while the pyridine moiety remains electron‐poor but *less deficient* than in isolated pyridine (due to partial electron donation from the fused partner). However, the nitrogen lone pair becomes less basic because of delocalization and inductive withdrawal from O or S. The electron rich ring typically undergoes electrophilic substitution while the electron‐poor pyridine component undergoes nucleophilic substitution.

#### 2.2.1 Deuteration Chemistry of Quinolines and Isoquinolines

##### Quinolines

**Scheme 11:**
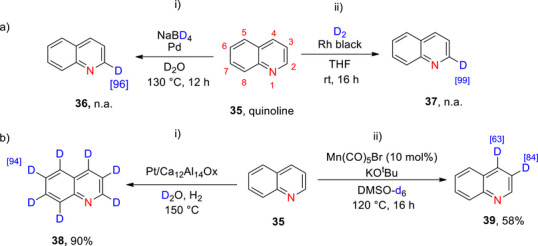
a)i) Deuteration of quinoline (**35**) by Alexakis et al.^[^ ^119^ ^]^ and a) ii)by Cao et al. ^[^ ^143^ ^]^ b) i) Deuteration of quinoline (**35**) by Jiang et al.^[^ ^144^ ^]^ and b) ii) deuteration of quinoline (**35**) by Kopf et al.^[^ ^145^ ^]^

Quinoline (35, Scheme 11), or benzo[*b*]pyridine, is a colorless oily hygroscopic liquid, with p*K*_a_ ≈ 4.85 in water at 20 °C. It undergoes both electrophilic and nucleophilic substitution reactions.^[^ ^142^ ^]^

###### Monodeuteration

Cao et al. used a palladium catalyst, NaBD_4_ and D_2_O to selectively deuterate the same position on the quinolinic ring with high 96% yield (Scheme 11a **(i)**).^[^ ^143^ ^]^ Alexakis et al.^[^ ^119^ ^]^ reported the selective deuteration at the C α to the N in quinoline using D_2_ gas and a heterogeneous rhodium catalyst (Rh black) (Scheme 11a **(ii)**). Interestingly, when either Ru black or a 5%Rh/alumina catalyst were used, the % D was lowered to 27% and 53%, respectively.

###### Polydeuteration and perdeuteration

Perdeuteration of quinoline (**42**) was reported by Jiang et al.^[^ ^144^ ^]^ using D_2_O, H_2_ and a Pt/Ca_12_Al_14_Ox catalyst with an average D content of 94% and yield of 90% (Scheme 11b(i)). Positively, from a sustainability perspective, the catalyst can also be reactivated and reused in subsequent reactions up to 16 times.

Kopf et al.^[^ ^145^ ^]^ selectively deuterated positions 3 and 4 of quinoline (compound 35) using a manganese complex (manganese pentacarbonylbromide) coupled with KO*^t^* Bu/DMSO‐d_6_. The process enabled high D‐incorporation (84%) at position 3 with moderate incorporation (63%) at position 4 (Scheme 11b(ii)). The overall % D incorporation was higher for pyridine derivatives than for quinoline. However, halogenated substrates afforded low yields in both cases of pyridines and quinolines, presumably resulting from competing halogen transfer reactions under the basic reaction conditions.^[^ ^145^ ^]^

**Scheme 12:**
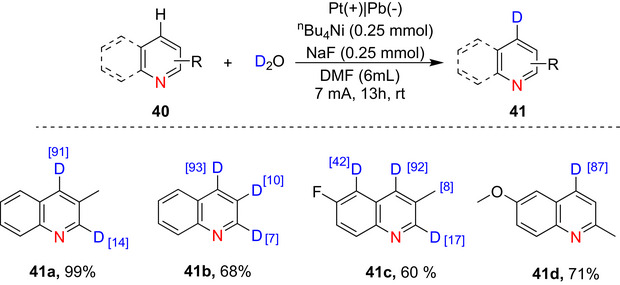
Electrochemical selective deuteriation of fused pyridine (**41a–41d)**. Ref: Qui et al.^[^ ^146^ ^]^

Qui et al.^[^ ^146^ ^]^ reported the electrochemical polydeuteration of substituted quinolines with D_2_O as the D source using an undivided electrochemical cell consisting of a Pt plate anode, lead plate cathode, with a constant current 7 mA at room temperature (Scheme 12).

##### Isoquinolines

Isoquinoline (**44**) is a structural isomer of quinoline. Both isoquinoline and quinoline are benzopyridines composed of a benzene ring fused to a pyridine ring.^[^ ^147^ ^]^ Analogous to quinoline, electrophilic attack in isoquinoline takes place in the benzene ring rather than in the deactivated pyridine ring (which is then further deactivated by protonation).

###### Monodeuteration

**Scheme 13:**
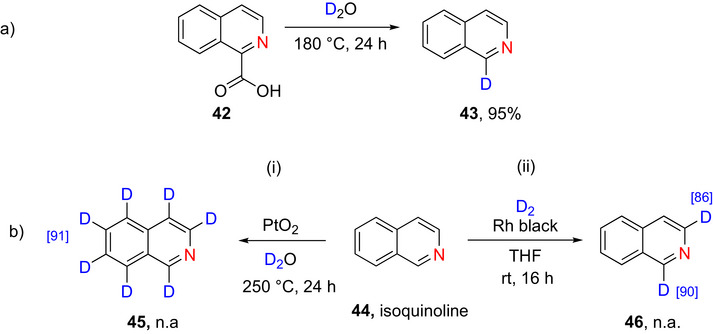
a) Monodeuteration of isoquinoline (**42**) by Stephens et al.^[^ ^148^ ^]^ b‐i) Perdeuteration of isoquinoline (**44**) using platinum dioxide catalyst to form **45**.^[^ ^149^ ^]^ b‐ii) Di‐deuteration of **44** to form **46** by Alexakis et al.^[^ ^119^ ^]^

Stephens et al.^[^ ^148^ ^]^ reported monodeuteration via deuterodecarboxylation of isoquinoline **43** starting from isoquinoline‐1‐carboxylic acid **42** (Scheme 13a). The reaction involves harsh conditions: heating 47 at 180 °C for 24 h in the presence of excess D_2_O. The resulting product was furnished in 95% yield (the %D‐incorporation was not reported).

###### Polydeuteration

Yamamoto et al.^[^ ^149^ ^]^ demonstrated the perdeuteration of isoquinoline (**44**) using a platinum dioxide catalyst to form **45** in an unknown yield but high % D (Scheme 13b **(i)**), while Alexakis and collaborators^[^ ^119^ ^]^ reported regioselective deuteration at positions 1 (86% D) and 3 (90% D) of **44** using D_2_ gas over a heterogeneous Rh catalyst. This latter reaction proceeded with high (86% and 90%) % D‐incorporation to form compound **46** (Scheme 13b **(ii)**).

#### 2.2.2 Acridine

**Scheme 14:**
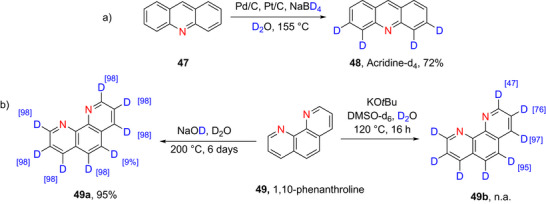
a) Deuteration of acridine **47** to form acridine‐*d*_4_ **48**.^[^ ^153^ ^]^ b) Deuteration of 1,10‐phenanthroline **49**, as demonstrated by Browne et al.^133^ and Clark.^[^ ^157^ ^]^

Acridine (**47**, Scheme 14) consists of three fused rings, providing a planar aromatic surface. Acridines possess three fused rings with one or two N atoms in their scaffold, respectively. Acridine derivatives are known to exhibit an array of bioactivities.^[^ ^150^ ^]^ For example, Amsacrine, a antineoplastic agent, has been used in the treatment of acute lymphoblastic leukemia.^[^ ^151^ ^]^ Furthermore, acridines find use as synthetic dye‐stuffs, optical materials, and sensors.^[^ ^152^ ^]^

There are three reports of deuteration of acridine in the literature. An early study (1973) by Fischer et al.^[^ ^130^ ^]^ reported the synthesis of perdeuterated acridine using a platinized asbestos as catalyst and D_2_O as the D source with 94% deuteration. Kupka et al.^[^ ^153^ ^]^ reported the preparation of partially deuterated acridine by a Pt/Pd catalyzed reaction (**48**, Scheme 14). The purpose of the study was to understand the effects of solvents on the crystallization of non‐, partial‐, and per‐deuterated acridines (“isotopomeric polymorphism”). A more recent study^[^ ^154^ ^]^ reported the synthesis of perdeuterated acridine using Pd(TFA)_2_ in the presence of D_2_O as deuterium source and deuterated 1,1,1,3,3,3‐hexafluoroisopropanol (HFIP‐d_1_) as cosolvent.

#### 2.2.3 Phenanthroline

Phenanthrolines are diazaphenanthrene analogs. The polyaromatic structure of phenanthrolines provide it with robustness and rigidity, and these lead to interesting and useful features in many disciplines (such as sensors, supramolecular chemistry, theranostics, luminous coordination scaffolds, etc.^[^ ^155^, ^156^ ^]^ 1,10‐Phenanthroline (**49**, Scheme 14b) is the most recurrent isomer.

Browne et al.^[^ ^133^ ^]^ obtained 1,10‐phenanthroline‐d_8_ (**49a** Scheme 14b) via regioselective H/D exchange using inexpensive reagents (1 M NaOD/D_2_O) at 200 °C in high % D (98% D incorporation at every position) and yield (95%) (Scheme 14). Clark et al.^[^ ^157^ ^]^ also prepared 1,10‐phenanthroline‐d_8_ (**49b**, Scheme 14b). In this case, a lower temperature (120 °C) and a strong base (KO*^t^* Bu) were employed. Interestingly, the % D was higher at C4 and 5 compared to at positions C2 and 3, likely due to a combination of steric and electronic effects.

#### 2.2.4 Deuteration of Pyridines Fused With Electron‐rich Heteroaromatics

##### Furopyridines

**Scheme 15:**
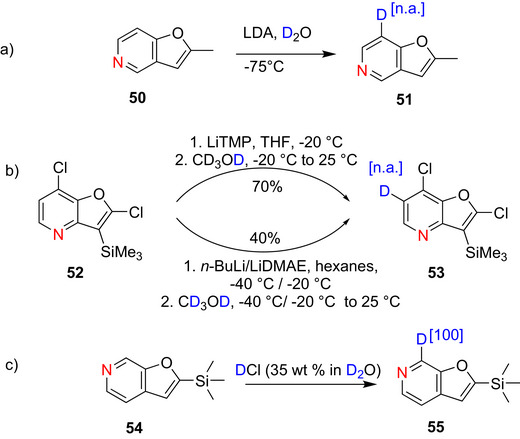
a) Deuteration of 2‐methylfuropyridine **50** reported by Shiotani et al.^[^ ^158^ ^]^ b) Deuteration of 2,7‐dichloro‐3‐trimethylsilylfuro[3,2‐*b*]pyridine **52** via D*o*M^[^ ^159^ ^]^ c) Deuteration of 2‐trimethylsilylfuro‐ [2,3‐*c*]pyridine **54** according to Chartoire et al.^[^ ^160^ ^]^

Furopyridines are fused heterocyclic compounds that consist of a furan ring and a pyridine ring fused together. These demonstrate a range of medicinal properties, including anticonvulsant, antipsychotic antiproliferative and anthelmintic, antianaphlactic activities. There are few selective reports on the deuteration of furopyridines. In 1993, Shiotani et al.^[^ ^158^ ^]^ prepared 2‐methyl‐7‐deuteriofuro[3,2‐c] pyridine (**51**) from 2‐methylfuropyridine (**50**) using lithium diisopropylamide (LDA) at − 75 °C and subsequent treatment with deuterium chloride in deuterium oxide, however in a low yield (∼15%) (Scheme 15a). Subsequently, deuteration of the functionalized furopyridine **52** was achieved by Jasselin–Hinschberger et al.^[^ ^159^ ^]^ using either LiTMP or *n*‐BuLi/LiDMAE to yield the *ortho*‐deuterated product **53** in 70% and 40%, respectively, although the % D incorporation wasn't reported (Scheme 15b). Similarly, Chartoire et al.^[^ ^160^ ^]^ reported the synthesis of 7‐deuterio‐2‐trimethylsilylfuro[2,3‐c]pyridine (**55**) from 2‐trimethylsilylfuro‐ [2,3‐c]pyridine (**54**) using deuterium chloride (35 wt % in deuterium oxide as electrophile, the product was obtained with a yield of 80% and 100% deuteration (Scheme 15c).

##### Imidazopyridines

Imidazopyridines are a class of nitrogen‐containing heterocyclic compounds formed by a fused imidazole and pyridine ring structure. A few reports of deuterated imidazopyridines are known. Kuriyama et al.^[^ ^161^ ^]^ reported the synthesis of 6‐deuterioimidazo[1,2‐*a*]pyridine by deuterodechlorination using palladium/unsymmetrical NHC system as catalyst in presence of Cs_2_CO_3_ as base and α‐deuterioalcohol as D source. Deuterated product was obtained with 99% yield and 99% of D‐incorporation. A plausible mechanism suggests that after forming an NHC‐ligated Pd complex in the presence of a base, the aryl chloride undergoes oxidative addition to the Pd(0) center, producing an aryl–palladium intermediate. Subsequent base‐mediated displacement of the chloride anion yields an α‐deuterioalkoxy–palladium species, which then undergoes β‐deuterium elimination to generate an aryl–palladium–deuteride complex. Finally, reductive elimination delivers the deuterated product while regenerating the Pd(0) catalyst.

**Scheme 16:**
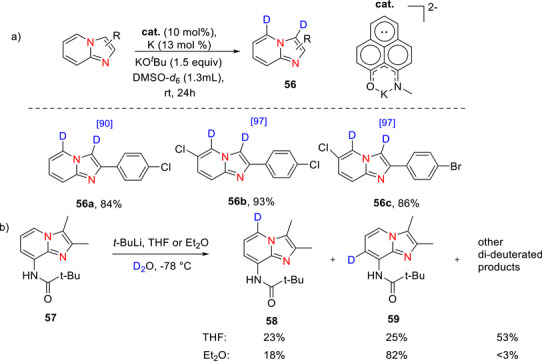
Synthesis of deutero‐deiodinated imidazopyridines **56a–56c**.^[^ ^162^ ^]^ b) Deuterated *N*‐pivaloyl imidazo[1,2‐*a*]pyridine **57** via D*o*M.^[^ ^163^ ^]^

Singh et al.^[^ ^162^ ^]^ recently reported TM‐free deutero‐deiodination of 3‐iodoimidazopyridines, compounds possessing demonstrated antibacterial activity. A reduced phenalenyl catalyst produced from 9‐(methylamino)‐1*H*‐phenalen‐1‐one, metallic K, and KO*^t^* Bu, was used with DMSO‐d_6_ to achieve excellent D incorporation (90%–97%, see compounds **56a**, **56b** and **57c,** Scheme 16a) at the 3‐position on the imidazole portion of the ring with high yields (84%–93%).

**Scheme 17:**
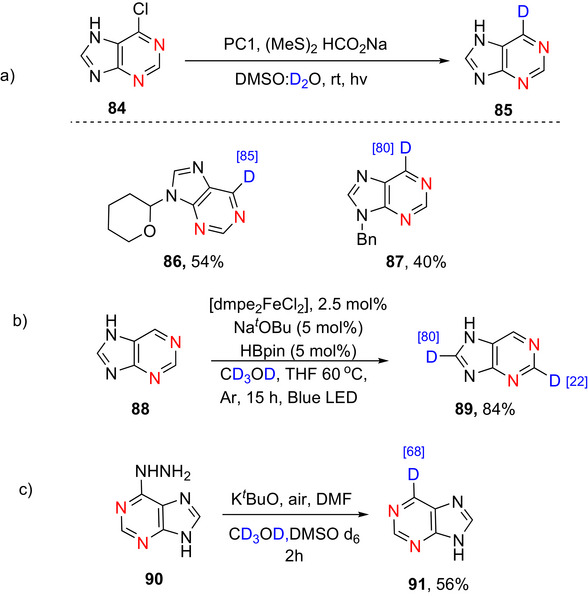
Proposed mechanism for the SET‐induced Catalytic Deutero–Dehalogenation of Aryl Halides (from Singh et al.^[^^162]^(Reproduced with permission) Copyright {2025} American Chemical Society.

The proposed mechanism of the deuterodehalogenation reaction is shown in Scheme 17. Initially, a phenalenyl‐KO*^t^* Bu (PLY‐K) complex **I** was formed by the interaction of the PLY ligand and KO*^t^* Bu (there is precedent for this type of reaction from other studies, e.g., by Paira et al.,^[^ ^164^ ^]^ simultaneously forming *^t^* BuOH). Then, a SET process from another KO*^t^* Bu to **I** generates a mono‐reduced PLY radical‐K complex **II** (contains 13 π electrons) and a O*^t^* Bu radical. Further reduction of **II** by K produces a doubly reduced PLY‐K complex **III** (now an anionic 14π electron species). Complex **III** has a high HOMO energy (−1.68 eV), enabling electron transfer via SET to the LUMO of the heteroaryl halide at r.t., leading to the formation of the heteroaryl halide radical anion **V**, which accepts a H from DMSO through a transition state **TS1**, thereby forming the desired hydrodehalogenated product **VIII** (this can be translated to a deuterodehalogenated product through the use of DMSO‐*d*_6_ instead of normal DMSO). DFT calculations (and a resultant energy diagram, shown in the paper by Singh et al.) show that the transition state **TS1** for this hydrogen atom transfer (HAT) process has an energy barrier that can be overcome at at room temperature to yield **VIII** and the DMSO‐based radical intermediate **IX**. **IX** can also undergo an additive interaction with ^−^O*^t^* Bu via transition state **TS2**, resulting in the formation of intermediate **X**, which can transfer an electron to complex **II** to regenerate the doubly reduced active catalyst (**III**) along with the formation of **XI**.

Board et al.^[^ ^163^ ^]^ reported the deuteration of an *N*‐pivaloyl imidazo[1,2‐*a*]pyridine (**57**) using *t*‐BuLi and D_2_O in either THF or diethyl ether. A mixture of products (**58**, **59** and other di‐deuterated products) was obtained using either solvent, but separation and isolation of the products were not mentioned. However, greater specificity was achieved with diethyl ether (Scheme 16b).

There are also studies where deuteration of imidazopyridines was reported to happen on the imidazo ring, instead of the 6 membered pyridine ring. For example, Balkenhohl^[^ ^193^ ^]^ reported the synthesis of imidazo[1,5‐*a*]pyridine‐3‐d_1_ using in situ‐generated LiTMP in the presence of zinc chloride, with D_2_O as deuterating agent.

##### Pyrrolopyridines

**Scheme 18:**
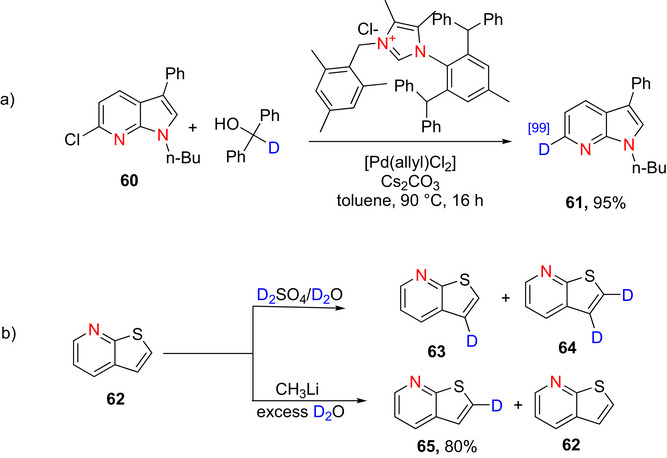
a) Preparation of deuterated pyrrolopyridine **61** according to Kuriyama et al.^[^ ^161^ ^]^ b) Preparation of deuterated thienopyridines **63**–**65** according to Klemm et al.^[^ ^171^ ^]^

Kuriyama et al.^[^ ^161^ ^]^ reported a Pd/unsymmetrical NHC system catalyzed synthesizes of deuterated pyrrolopyridines using lithium aluminum deuteride in THF via deuterodechlorination of 1‐butyl‐6‐chloro‐3‐phenyl‐1H‐pyrrolo[2,3‐*b*]pyridine (**60**) to give 1‐butyl‐3‐phenyl‐1H‐pyrrolo[2,3‐*b*]pyridine‐6‐d (**61**) with 99% D incorporation and 95% yield (Scheme 18a). Other reported synthetic procedures to deuterated pyrrolopyridines involved only deuteration of the pyrrole ring. For example, Li et al.^[^ ^165^ ^]^ reported the synthesis of 1*H*‐pyrrolo[2,3‐*b*]pyridine‐3‐d_1_ (95% D) using a nanostructured iron catalyst via H/D exchange. Similarly, Dong et al.^[^ ^166^ ^]^ reported the synthesis of various pyrrolopyridines with high (>84%) D incorporation using AgOTf as catalyst and D_2_O as the deuterium source via α‐deuteration of the pyrrolopyridines,. In another study, Milcendeau et al.^[^ ^167^ ^]^ demonstrated a gold(I)‐catalyzed hydrogen isotope exchange reaction on pyrrolopyridine under mild conditions and low catalyst loadings, using CD_3_OD and D_2_O as readily available deuterium sources, to give 1*H*‐pyrrolo[2,3‐*b*]pyridine‐3‐*d*_1_ with 30% D incorporation. Fitzgerald et al.^[^ ^168^ ^]^ also reported Pd‐catalyzed deuteration to furnish a dideuterated pyrrolopyridine with 90% and 11% D incorporation at the C2 and C3 position of the pyrrole ring, respectively.

##### Thienopyridines

Thienopyridines are heteroaromatic compounds consisting of a thiophene ring fused with a pyridine ring. Several significant drugs contain thienopyridine moieties, namely Prasugrel and Clopidogrel,^[^ ^169^ ^]^ and Ticlopidine.^[^ ^170^ ^]^ Klemm et al.^[^ ^171^ ^]^ reported the only recorded synthesis of deuterated thienopyridines, namely that of 3‐deuteriothieno [2,3‐*b*] pyridine **63** and 2,3‐dideuteriothieno‐[2,3‐*b*]pyridine **64**, as follows. Thieno [2,3‐*b*]pyridine (**62**) was reacted with deuteriosulfuric acid in D_2_O to furnish **63** and **64** (Scheme 18b). Similarly, upon reaction with methyllithium and an excess of D_2_O, thieno [2,3‐*b*] pyridine was deuterated to form 2‐deuteriothieno[2,3‐*b*]‐ pyridine (**65**) (80% yield) and recovered thieno[2,3‐*b*]pyridine (**62**) as side product. Though these reports show D incorporation on the thiophene ring, there are no reports on thienopyridines with D incorporation on the 6 membered ring.

It should be noted that there is no record in the literature of any deuterated selenolopyridines, despite the relevance of selenopyridines in many medicinal applications.^[^ ^172^, ^173^, ^174^ ^]^ This is a significant research gap for future exploration.

### 2.3 Diazines

*Structure, Properties, and Reactivity of the Diazines*

**Figure 4:**
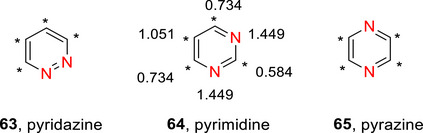
π‐Electron densities of pyrimidine, **64**. The asterisks indicate the electron‐deficient positions α‐ and γ‐ to N in **63**‐**65**.^[^ ^11^, ^71^ ^]^

The diazines (Figure 4) tend to be slightly more reactive than pyridine due to the presence of two nitrogen atoms, which results in the adjacent carbons being more electron deficient and therefore more prone to nucleophilic addition and less susceptible to electrophilic substitution.^[^ ^72^ ^]^ This is partially due to the polarization caused by the additional N that further increases the partial‐positive charge on the α/γ‐carbons. Other than the C‐5 of pyrimidine, all ring carbons in the diazines are positioned *ortho* or *para* to at least one nitrogen (Figure 4), explaining the high susceptibility to nucleophilic attack at these positions. Using pyrimidine as an example, nucleophilic substitutions would preferentially occur at C‐2 and C‐4(6), whereas electrophilic substitution requires very particular conditions and would be least disfavoured at C‐5.

Diazines are significantly less basic than pyridine due to the destabilization of the conjugate acid by inductive electron withdrawal by the second nitrogen (pyridazine **63** (p*K*_a_ ∼ 2.3), pyrimidine **64** (p*K*_a_ ∼1.3), and pyrazine **65** (p*K*_a_ ∼ 0.65) (Figure 4).^[^ ^10^ ^]^ Pyridazine has the strongest dipole of the 6‐membered azo‐containing rings, with a value of 4.22 D (this is almost double that of pyridine (μ = 2.22 D) and pyrimidine (μ = 2.33 D)).^[^ ^175^ ^]^

Deuteration of the diazines can be achieved similarly to pyridine, as described in the following subsections.

#### 2.3.1 Pyrimidines

##### Lithiation–Deuteration: Lithium–Halogen Exchange and DoM

###### Monodeuteration

Pyrimidine is more susceptible to nucleophilic attack than pyridine, especially at the C‐2 position.^[^ ^10^, ^11^, ^71^, ^74^, ^176^ ^]^ Alkyl‐ or aryllithiums readily add to carbons adjacent to the nitrogen atoms of the diazines, leading to substitution of hydrogen with an alkyl or aryl group.^[^ ^10^ ^]^ Even with halogenated diazines, addition can occur at nonhalogenated carbons.^[^ ^10^ ^]^ Lithiation of the diazines therefore may be achieved using particularly nonnucleophilic bases (*e.g*., LiTMP and LDA) or with alkyllithium compounds through lithium‐halogen exchange at very low temperatures to avoid nucleophilic addition. ^[^ ^65^ ^]^ D*o*M can also be employed with LiTMP or LDA, commonly using chloro‐ and alkoxy‐derivatives.^[^ ^10^, ^11^, ^71^, ^74^, ^176^ ^]^ Recent reviews by Quéguiner and Mongin,^[^ ^60^, ^61^, ^62^ ^]^ Gros,^[^ ^63^ ^]^ and Kolarovič^[^ ^177^ ^]^ include lithiation‐deuteration of pyrimidine up to 2014.

**Scheme 19:**
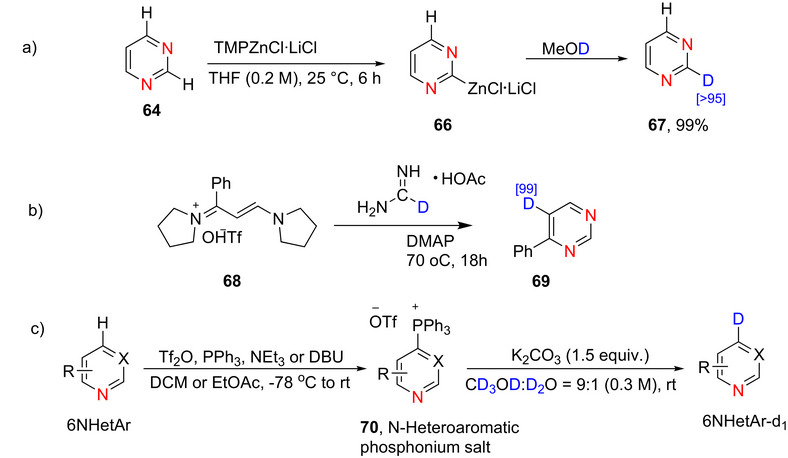
a) Zincation and deuteration of pyrimidine **64**.^[^ ^178^ ^]^ b) Synthesis of deuterated pyrimidine **69** via deconstruction–reconstruction strategy according to Uhlenbruck et al.,^[^ ^179^ ^]^ c) Method developed by Koniarczyk et al.^[^ ^96^ ^]^ involving the formation of a phosphonium salt **70** which can be transformed into a mono‐deuterated species via deuterodephosphoniation of the PPh_3_ group.

In 2022, Kremsmair et al. introduced D onto position 2 of pyrimidine (**64**) to afford **67** by using a zincation procedure mediated by TMPZnX·LiX (X = Cl, Br, see compound **66** for the chloro compound)^[^ ^178^ ^]^ (Scheme 19a).

Uhlenbruck et al.^[^ ^179^ ^]^ reported the synthesis of deuterium labelled pyrimidine via a deconstruction–reconstruction strategy. In this work, the authors deconstruct pyrimidines into vinamidinium salts before reconstructing them with deuterated, ^13^C, and ^15^N‐enriched amidines. The process involves a Tf_2_O‐mediated ring‐opening and ring‐closing sequence to form a pyrimidinium ion, followed by cleavage to the vinamidinium salt with pyrrolidine, followed by cyclization with labelled amidines under basic conditions to furnish the isotope labelled pyrimidine. This strategy was used to prepare pyrimidines deuterated at the 5‐position (Scheme 19b).

##### Deutero‐Defunctionalisation

Recent reports detailing deutero‐defunctionalisation reactions of pyrimidines are shown in Table 8 and discussed as follows.

**Table 8:** Monodeuteration of pyrimidines via defunctionalisation.

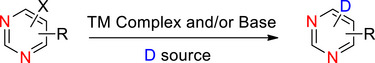					
Entry	X (D)	R	Conditions	% Yield (% D)	Refs.
1	4‐PPh_3_^+^OTf^−^	2‐Cl, 5‐Et	K_2_CO_3_, CD_3_OD, D_2_O, rt	60 (n.a)	[96]
2	5‐Br	2‐OMe	Bu_3_SnH, V‐65, D_8_‐THF, reflux	78 (92)	[180]
3	5‐Br	2‐(4‐Boc‐piperazin‐1‐yl)	Pd(dba)_2_, K_2_CO_3_, S‐Phos, D_8_‐IPA, MeCN, 100 °C	95 (≥ 95)	[100]
4	5‐Br	2‐NH_2_	Cu NWAs cathode, K_2_CO_3_, MeCN, D_2_O, rt	86 (80)	[91]
5	6‐Cl	2,4‐Me_2_	10% Pd/C, Na_2_CO_3_, CH_3_OD, D_2_, rt	87 (n.a)	[182]
6	2,5‐Cl_2_	‐	5% Pd/C, K_2_CO_3_, THF, D_2_O, D_2_, rt	66 (> 99)	[103]

###### Deuterodephosphoniation

An interesting dephosphoniation of a preprepared heterocyclic PPh_3_^+^OTf^−^ salt by Koniarczyk et al.^[^ ^96^ ^]^ provided the corresponding deuterated pyrimidine using CD_3_OD:D_2_O as the D source (Scheme 19c. For example, 4‐deuterated 2‐chloro‐5‐ethylpyrimidine could be prepared in this way, in 60% yield (no % D incorporation reported) (Table 8, Entry 1).

###### Deuterodehalogenation

**Scheme 20:**
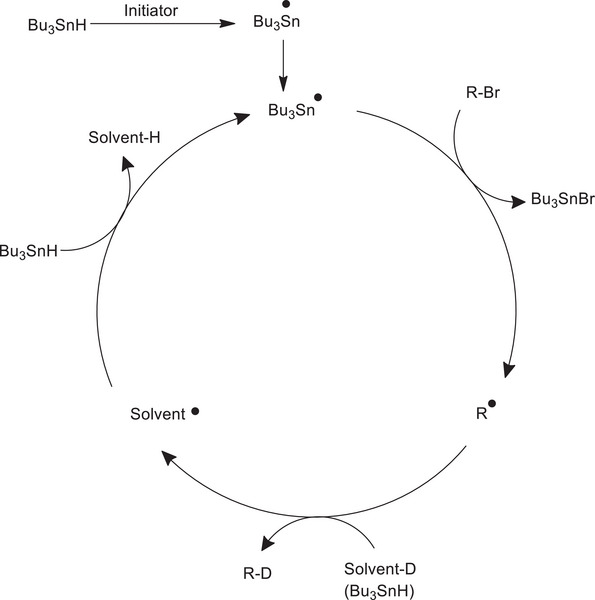
Proposed mechanism of deutero‐debromination according to Mutsumi et al.^[^ ^180^ ^]^ Adapted with permission from Elsevier.

Mutsumi et al.^[^ ^180^ ^]^ deutero‐debrominated 5‐bromo‐2‐methoxypyrimidine in good yield and with a high % D incorporation using tributyltin hydride (Bu_3_SnH) in THF‐d_8_ (Table 8, Entry 2). Importantly, nucleotides and nucleobase derivatives have been similarly deuterated using this methodology, despite its harsh reaction conditions. Mutsumi et al.^[^ ^180^ ^]^ deutero‐debrominated 5‐bromo‐2‐methoxypyrimidine in good yield and high % D incorporation using tributyltin hydride (Bu_3_SnH) in THF‐d_8_ (Table 8, Entry 2). Importantly, nucleotides and nucleobase derivatives have been similarly deuterated using this methodology. The reaction occurs via radical chemistry, as Bu_3_SnH is a radical‐generating reagent. Thus, the tributyltin radical species formed in the initiation step removes a bromine atom from the 5‐bromo‐2‐methoxypyrimidine, forming Bu_3_SnBr and an aryl radical intermediate, which removes a D radical from the carbon adjacent to the oxygen atom of the deuterated solvent to afford the corresponding 2‐methoxypyrimidine 5‐d_1_ (Scheme 20).^[^ ^180^ ^]^ However, Bu_3_SnH is not a green reagent (it is highly toxic and its side products are not easily worked up/removed) and recent studies propose the use of tributylgermanium hydride (Bu_3_GeH) as a more sustainable alternative,^[^ ^181^ ^]^ although this reagent has not been studied for these types of reactions.

**Scheme 21:**
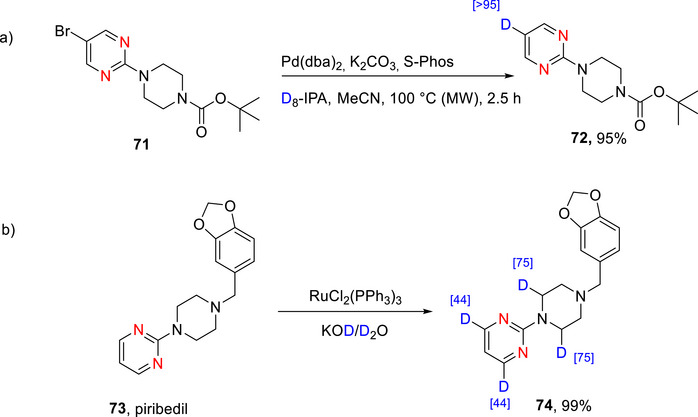
a) Synthesis of deuterated complex pyrimidine **72** by Pd(dba)_2_‐catalyzed deuterodebromination.^[^ ^100^ ^]^ b) Synthesis of piribedil‐d_4_ (**74**) via Ru catalyzed HIE.^[^ ^117^ ^]^

Donald et al.^[^ ^100^ ^]^ employed deutero‐debromination on a piperazine‐substituted pyrimidine **61** (Scheme 21a) using a Pd(dba)_2_ catalyst. This provided the corresponding deuterated heterocycle **62** in high yield and with excellent D incorporation (Table 8, Entry 3).

Zhang's^[^ ^91^ ^]^ electrocatalytic protocol ‐ based on recyclable copper nanowire arrays as cathode, resulted in the deutero‐debromination of 2‐amino‐5‐bromopyridine in high yield and D‐incorporation (Table 8, Entry 4).

Two similar reactions performed by Pavlik^[^ ^182^ ^]^ and Rayner^[^ ^103^ ^]^ utilized heterogeneous Pd catalysis (Pd/C) to achieve deuterodehalogenation of chloro‐pyrimidines under mild conditions (Table 8, Entry 5–6). Pavlik reported a good yield (87%), yet omitted to report the extent of deuteration, whereas Rayner reported a lower yield (66%) and quantitative (> 99% D) D incorporation. Regrettably, neither group attempted to recycle the catalysts in subsequent runs to demonstrate the sustainability of the process, an aspect gaining significance globally.^[^ ^120^ ^]^

##### Transition‐Metal or Base Catalyzed HIE

###### Dideuteration

Das et al.^[^ ^183^ ^]^ deuterated the 5‐ and amino positions of 2‐*t*‐butylaminopyrimidine in > 99% D incorporation using a Pd catalyst (Pd(OAc)_2_) and CD_3_COOD as D source (Table 9, Entry 1). This deuterated compound was also synthesized using a lithiation–deuteration method,^[^ ^183^ ^]^ although no % D incorporation was reported.

**Table 9:** Dideuteration of Pyrimidine via H/D Exchange.

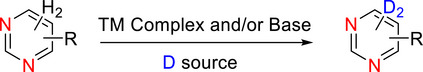					
Entry	Position(s) of H/D	R	Conditions	%Yield (% D)	Refs.
1	C5, NH	2‐NH*t*Bu	Pd(OAc)_2_, Na_2_CO_3_, Ag_2_CO_3_, CD_3_COOD, 120 °C	n.a (>99)	[183]
2	C4, C6‐	2‐(4‐tolyl)	[RuCl_2_(*p*‐cymene)]_2_, AgOAc, PPh_3_, [D_4_] acetic acid, 150 °C	89 (60)	[184]
3	C4, C6	2‐(4‐morpholine), 5‐F	Ag_2_CO_3_, S‐Phos, K_2_CO_3_, Toluene, 90 °C	93 (92)	[113]
4	C4, C6	2‐Ph, 5‐F (**65**, Scheme xx)	Ag_2_CO_3_, S‐Phos, K_2_CO_3_, Toluene, 90 °C	86 (84) (**65**‐*d*_2_)	[113]
5	C2,C5	2‐Cl 2‐Cl	5% Pd/C, K_2_CO_3_, THF, D_2_O, D_2_, rt	n.a (5)	[103]

**Scheme 22:**
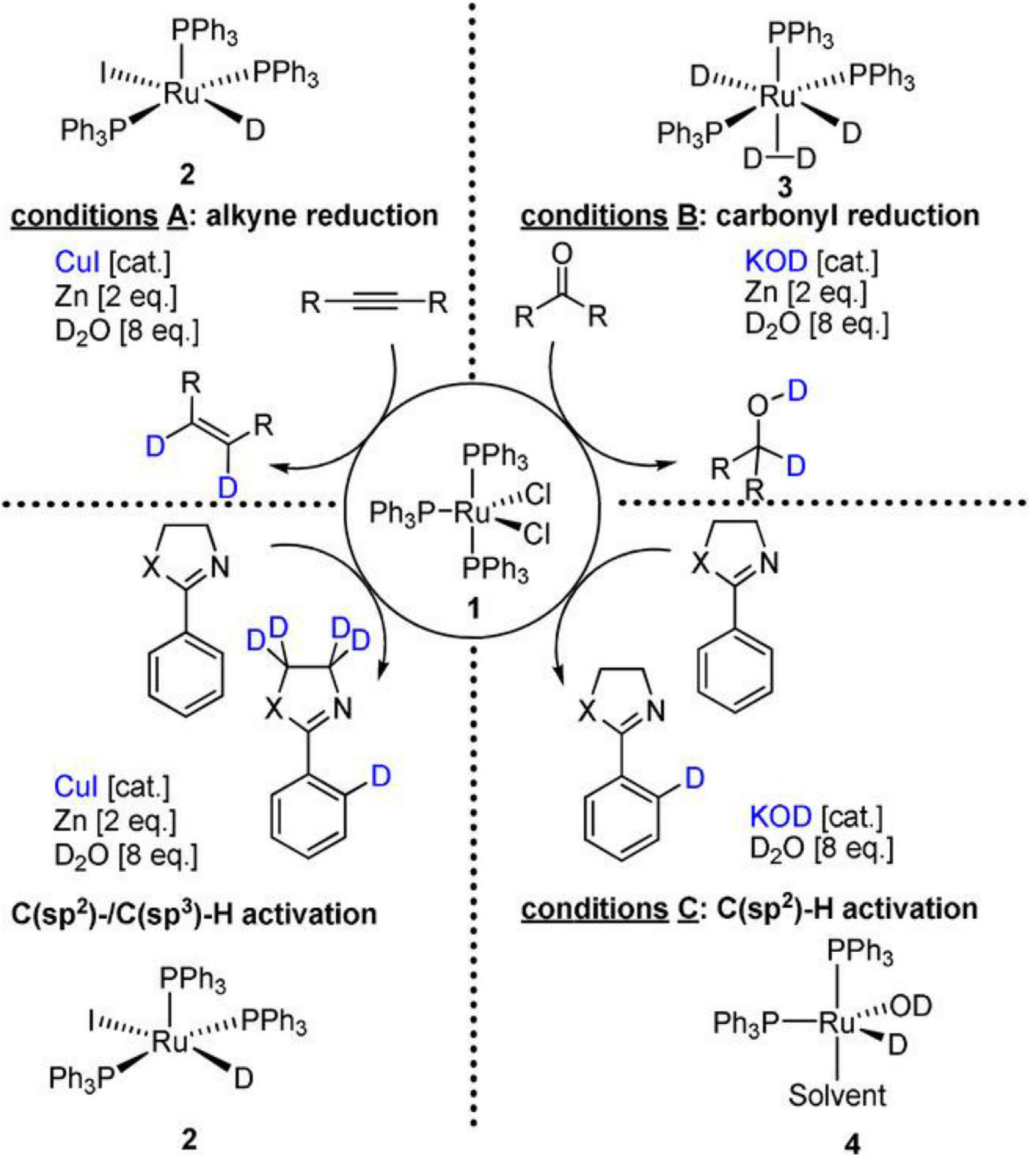
Additive‐dependent chemoselective Ru‐catalyzed deuterations according to Eisele et al.^[^ ^117^ ^]^ (Reproduced with permission under the terms of the Creative Commons CC BY license).

Eisele et al.^[^ ^117^ ^]^ reported the deuteration of the pyrimidine‐containing drug piribedil **73** (anti‐Parkinson's disease) in quantitative yield using a RuCl_2_(PPh_3_)_3_ catalyst and KOD/D_2_O (Scheme 21b, compound **74**). The procedure enabled H/D exchange at the 4,6‐positions of the pyrimidine unit in moderate D incorporation (44%) and the 2,6‐positions of the piperazine unit in better D incorporation (75%). The authors probed the mechanism of the Ru‐catalyzed deuteration and determined that the process occurs with additive‐dependent chemoselectivity:, i.e., by varying the additive between CuI and KOD, it was possible to control the type of transformation and therefore the position of deuteration (Scheme 22).

Zhao et al.^[^ ^184^ ^]^ di‐deuterated 2‐(4‐tolyl)‐substituted pyrimidine at the 4 and 6‐positions (60% D at each position) and on the tolyl group at the in 89% yield using a Ru catalyst ([RuCl_2_(p‐cymene)]_2_) and deuterated acetic acid (Table 9, Entry 2). Additional deuteration occurred in all unsubstituted ring positions of the toluene substituent.

**Scheme 23:**
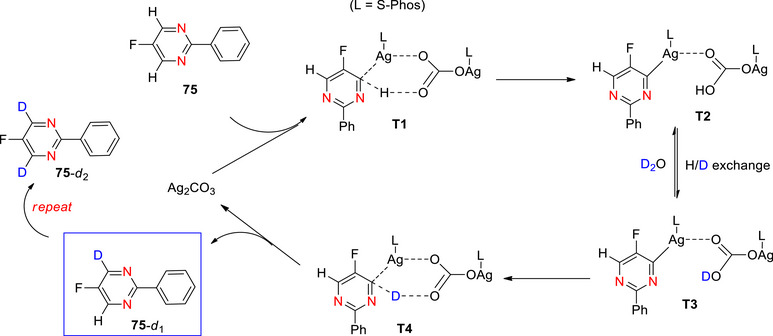
Proposed mechanism for the Ag(I)(S‐Phos‐mediated H/D exchange of fluoropyrimidine **75** to form **75**‐*d*_1_ and **75**‐*d*_2_ (adapted from Hu et al).^[^ ^113^ ^]^ Reproduced with permission from Royal Society of Chemistry.

Hu et al.^[^ ^113^ ^]^ accomplished deuteration of 5‐fluoropyrimidines with phenyl and morpholine substituents in high yield and D incorporation using Ag_2_CO_3_/S‐Phos mediated H/D exchange (Table 9, Entry 3–4). These reactions tended to select for the ring positions *ortho* to the fluorine, likely due to the greater acidity of the H next to the C─F bond.^[^ ^113^ ^]^ The proposed mechanism involves an initial deprotonation step, leading to C─H bond activation of the fluorinated (N‐hetero)arenes, e.g., pyrimidine **75** (Scheme 23). This is followed by H/D exchange between intermediate **T2** and D_2_O to form **T3**, followed by incorporation of the D (twice if the cycle is repeated) and regeneration of Ag_2_CO_3_.

Rayner et al.,^[^ ^103^ ^]^ demonstrated dideuteration of Pyrimidine via deutero‐dechlorination of 2,5‐dichloropyrimidine using K_2_CO_3_ and 5% Pd/C catalyst, with a low yield (Table 9, Entry 5).

###### Polydeuteration

One of the early reports of polydeuteration of pyrimidines was reported by Hervieu et al.^[^ ^185^ ^]^ in which deuteriopyrimidines of high isotopic purity were synthesized either by reductive dechlorination of chloropyrimidines using a Pd/CaCO_3_ catalyst (and D_2_ as D source), deuterolysis of sulfonylhydrazinopyrimidines with mild base in D_2_O, or oxidation of hydrazinopyrimidines with silver acetate.

Other recent examples of polydeuteration of pyrimidines are shown in Table 10 below.

**Table 10:** Polydeuteration of pyrimidines via HIE.

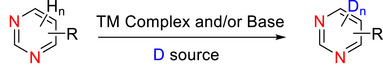					
Entry	Position(s) of H/D	R	Conditions	%Yield (% D)	Refs.
1	C4, C5, C6	2‐NH_2_	10% Pd/C, H_2_, D_2_O, 160 °C	99 (85)	[134]
2	C4, C5, C6	2‐NH_2_	10% Pd/C, H_2_, D_2_O, 160 °C	72 (74)	[186]
3	C2, C4, C5, C6	‐	CH_3_ONa, CH_3_OD, 165 °C	45 (82)	[187]
4	C2, C4, C6	‐	[Dmpe_2_FeCl_2_], NaO*t*Bu, HBPin, CD_3_OD, THF, 60 °C, blue light	90 ((36 (H2), 18 (H4, H6))	[188]
5	C2, C5	4‐Ph	[Dmpe_2_FeCl_2_], NaO*t*Bu, HBPin, CD_3_OD, THF, 60 °C, blue light	95 ((77 (H2), 31 (H5)	[188]

Tri‐deuterated pyrimidines have been reported, with tri‐deuterated 2‐aminopyrimidines prepared by Esaki^[^ ^134^ ^]^ and Mukuta^[^ ^186^ ^]^ using 10% Pd/C in the presence of H_2_ and D_2_O (Table 10, Entry 1 and 2). Using the same catalyst and reagents, Mutsumi et al.^[^ ^180^ ^]^ achieved the best yield and D incorporation.

An early example of perdeuterated pyrimidine was reported by Zoltewicz et al.^[^ ^187^ ^]^ in moderate yield and high D incorporation using CH_3_ONa and CH_3_OD under harsh conditions (Table 10, Entry 3).

**Scheme 24:**
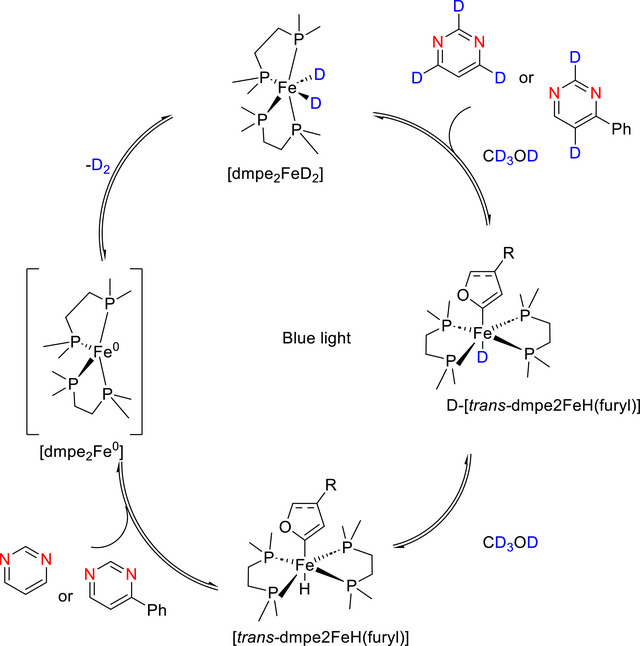
Proposed reaction mechanism for the iron‐catalyzed H/D exchange reaction of heteroarenes reported by Britton et al.^[^ ^188^ ^]^ Reproduced with permission from Royal Society of Chemistry under a Creative Commons Attribution‐NonCommercial 3.0 Unported Licence.

Deuteration of a mixed substrate scope including both arenes and heteroarenes (including pyrimidine) was achieved using an iron bisdiphosphino complex ([dmpe_2_FeH_2_] (dmpe = Me_2_PCH_2_CH_2_PMe_2_), in the presence of blue light and with CD_3_OD as the D‐source.^[^ ^188^ ^]^ A mix of deuterated pyridmidines were obtained in high yields and with moderate % D incorporation (Table 10, Entries 4–5). It was interesting to note that with 4‐phenylpyrimidine as substrate (entry 5), deuteration occurred at both C‐2 and C‐5 positions with significantly higher incorporation for than the unsubstituted analogue (entry 4). This makes sense according to the proposed reaction mechanism, shown in Scheme 24, since the iron‐hydride catalyst undergoes C─H activation at these electron‐deficient sites to give metallated intermediates that are further stabilized by conjugation with the phenyl substituent, thereby lowering the barrier for activation. In the catalytic cycle, the precatalyst [dmpe_2_FeCl_2_] is converted into the active iron‐hydride complex [dmpe_2_FeH_2_] which mediates C─H metallation of the pyrimidine to form an aryl‐iron hydride intermediate. Subsequent reversible protonation with CD_3_OD exchanges the hydride ligand for a deuteride, generating an iron‐deuteride species. Finally, reductive elimination releases the deuterated pyrimidine and regenerates the iron‐hydride catalyst, thus completing the H/D exchange cycle

##### Pre‐Deuterated Reagents

**Scheme 25:**
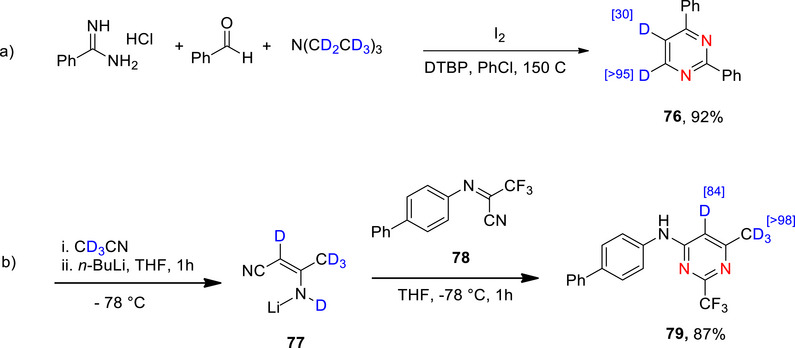
a) Preparation of deuterated pyrimidines **76** via condensation‐cyclization of Benzamidine hydrochloride, benzaldehyde.^[^ ^189^ ^]^ b) Trifluoroacetimidoylnitrile cyclization with to form deuterated pyrimidine **79**.^[^ ^190^ ^]^

Gao et al.^[^ ^189^ ^]^ produced a variety of pyrimidines from amidines using a dehydrogenative multicomponent cyclization protocol^[^ ^189^ ^]^ and D‐labelling in the resultant pyrimidines was performed to provide mechanistic insights. Benzamidine hydrochloride, benzaldehyde, and triethylamine‐*d*_15_ were reacted using I_2_, di‐tert‐butyl peroxide (DTBP), and PhCl at 150 °C to produce 5,6‐dideutero‐2,4‐diphenylpyrimidine **76** in 92% yield. D incorporations of 30% and > 95% were observed at the 5‐ and 6‐positions, respectively (**76**, Scheme 25a). These harsh conditions (with low % D incorporation at C‐5) are unlikely to be favourable for future synthesis of deuterated pyrimidines.

Another cyclization reaction performed by Li et al.^[^ ^190^ ^]^ produced 6‐methyl‐2‐trifluoromethylpyrimidines with various substituents at the 4‐position. Mechanistic experiments were performed with MeCN‐d_3_, which was reacted with *n*‐BuLi to produce the lithium intermediate **77** and then a biphenyl trifluoroacetimidoylnitrile **78** to produce 4‐​pyrimidinamine, N‐​[1,​1′‐​biphenyl]​‐​4‐​yl‐​6‐​methyl‐​2‐​(trifluoromethyl)​‐ **79** in 87% yield. Tri‐deuteration was achieved in > 98% on the methyl substituent at the 2‐position, and 84% D at the 5‐position of the pyrimidine ring (Scheme 25b).

#### 2.3.2 Fused Pyrimidines

Fused pyrimidines are heterocyclic compounds in which a pyrimidine ring is fused with another aromatic or nonaromatic ring. This fusion expands the structural diversity and electronic properties of the pyrimidine core, often enhancing biological activity and stability. Many fused pyrimidines occur naturally or are synthetically designed, and they form the backbone of several pharmacologically active molecules, including antiviral, anticancer, and antimicrobial agents.^[^ ^191^ ^]^ Notable examples include quinazoline (pyrimidine fused with benzene), which is widely studied as an anticancer and antimicrobial scaffold; furo‐ and thienopyrimidines (fusion with furan or thiophene), which exhibit strong antiviral, anti‐inflammatory, and kinase inhibitory activities; pyrrolopyrimidines (fusion with pyrrole), important in nucleoside analogs and kinase inhibitor drugs; and imidazopyrimidines (fusion with imidazole), known for broad pharmacological potential including antimicrobial and CNS activity. These fused systems are considered privileged scaffolds in medicinal chemistry, making them valuable in drug design and development. The are several reports on the synthesis of deuterated fused pyrimidine, especially quinazoline, imidazopyrimidines, and thienopyrimidines, discussed in this section.

##### Quinazoline

**Scheme 26:**
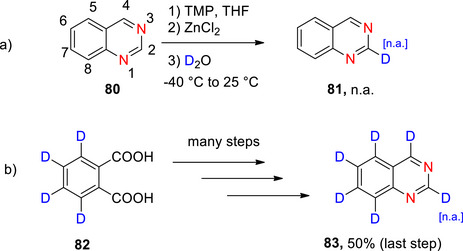
a) Chemical structure showing numbering of quinazoline **80** and preparation of deuterated quinazoline **81** according to Balkenhohl et al.^[^ ^193^ ^]^ b) Preparation of perdeuterated quinazoline **83** according to Verbeeck et al.^[^ ^194^ ^]^

Quinazoline (**80,** Scheme 26a) is an aromatic heterocycle with a bicyclic structure consisting of a pyrimidine and benzene ring fused together. Nitration is the only known electrophilic substitution reaction of quinazoline. Considering the aromatic electrophilic substitution, the expected order of reactivity is at positions 8 > 6 > 5 > 7 > 4 > 2.^[^ ^192^ ^]^

###### Monodeuteration

Balkenhohl et al.^[^ ^193^ ^]^ reported the monodeuteration of quinazoline at C2 (see compound **80**, Scheme 26a) using in situ‐generated LiTMP in the presence of Zinc chloride, with D_2_O as deuterating agent. The authors didn't report the %D incorporation.

###### Polydeuteration

Verbeeck et al.^[^ ^194^ ^]^ described the perdeuteration (see **83**) of quinazoline starting from phthalic acid‐d_4_ **82** (Scheme 26b). The final step employed Pd/C and D_2_O under an H_2_ atmosphere to afford the perdeuterated quinazoline in 50% yield. No substituted quinazolines were deuterated using this approach.

##### Imidazopyrimidines

**Scheme 27:**
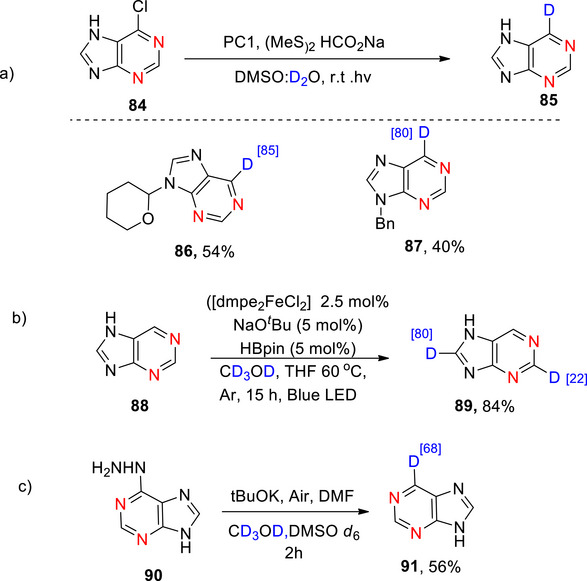
Synthesis of imidazopyrimidines **85**–**87** via deuterodehalogenation according to Li et al.^[^ ^108^ ^]^ b) Photocatalytic synthesis of deuterated imidazopyrimidines **89** reported by Britton et al.^[^ ^188^ ^]^ and c) Synthesis of deuterated imidazopyrimidines **91** via deuterodehydrazination according to Zhang et al.^[^ ^196^ ^]^

Imidazopyrimidines or purines are a very important class of organic compound known for their biological and pharmaceutical importance, there are few selected reports on the synthesis of deuterated imidazopyrimidines. An early report concerned with the deuteration of imidazopyrimidines was reported by Elvidge et al.,^[^^195]^ wherein HIE reactions were carried out using D_2_O to form imidazopyrimidine‐d_1_ with H/D exchange happening on the imidazole ring. In 2021 Li et al.^[^^108]^ reported the synthesis of deuterated imidazopyrimidines derivatives with organic catalysts (described in section 2.1.1) via deuterodehalogenation. This method gave deuterated imidazopyrimidines with D incorporation on the pyrimidine ring (Scheme 27a). Similarly, Britton et al.,^[^^188]^ reported a photocatalytic synthesis of deuterated imidazopyrimidines using an iron bisdiphosphino complex ([dmpe_2_FeH_2_] (dmpe = Me_2_PCH_2_CH_2_PMe_2_)‐catalyzed H/D exchange with CD_3_OD as the deuterium source, this method formed deuterated imidazopyrimidines with 22% D incorporation on the pyrimidine ring and 80% D incorporation on the imidazole ring the most acidic C(sp^2^)─H site (Scheme 27b). In another report, Zhang et al.^[^^196]^ reported the synthesis of mono deuterated imidazopyrimidines with D incorporation on the 6 membered ring, via KO^*t*^Bu‐promoted aerobic deuterodehydrazination of arylhydrazines in the presence of CD_3_OD/DMSO‐d_6_ as D‐source. The reaction involved deutrodehydrazination of 6‐hydrazinyl‐9*H*‐purine (**90**) to give 9*H*‐purine‐6‐d_1_ (**91**) (Scheme 27c).

##### Thienopyrimidines

**Scheme 28:**
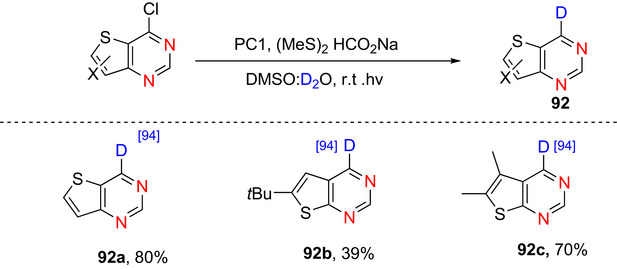
Synthesis of thienopyridines **92a–92c** according to Li et al.^[^ ^108^ ^]^

Li et al.^[^^108]^ reported the synthesis of thienopyridines using an organic photocatalyst ‘PC1’, previously mentioned in section 2.1.2. Thieno[3,2‐*d*]pyrimidine and thieno[2,3‐*d*]pyrimidines (**92a‐c**) (Scheme 28) were prepared by deuterodehalogenation of the corresponding thienopyrimidine chlorides.

#### 2.3.3 Pyrazines

**Scheme 29:**
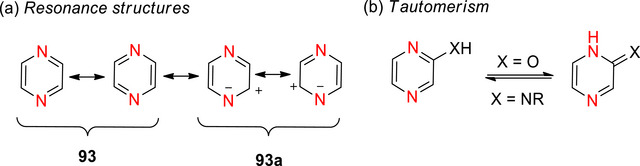
The resonance structures of pyrazine, showing the positions of reactivity on the ring.^[^ ^197^ ^]^

Pyrazine is a planar six‐membered N‐heterocyclic aromatic compound possessing a ring structure containing four C atoms and two N atoms positioned directly across from each other. Pyrazine has two resonance structures: **93**, ne in which each N atom has a lone pair of electrons, and **93a**, a dipolar resonance structure (Scheme 29a).

Pyrazine is a weaker base than pyridine and the weakest base of all the diazines. Thus, it is difficult to displace a hydrogen atom from a pyrazine ring. Electrophilic substitution requires the presence of activating groups on the pyrazine ring (or the use of N‐oxides). Nucleophilic reactions with substituted pyrazines are easier and halopyrazines are quite reactive (more reactive than the corresponding pyridines). Finally, substituted pyrazines demonstrate tautomerism (Scheme 29b), which accounts for the lack of reactivity of, e.g., pyrazin‐2‐ol (2(1*H*)‐pyrazinone) in forming *O*‐heteroaryl carbamates or *S*‐heteroaryl thiocarbamates.^[^ ^197^ ^]^

##### Lithiation–Deuteration: DoM and Lithium–Halogen Exchange

An early procedure reported by Rizzi^[^^198]^ involved the lithiation‐deuteration of 2,3‐dialkylpyrazines. In this case, 2,3‐dimethylpyrazine was reacted with MeLi and D_2_O in Et_2_O, providing 83%, 12%, and 1.3% of the mono‐, di‐, and tri‐deuterated products, respectively (Table 11, Entry 1). 2‐Ethyl‐3‐methylpyrazine was then subject to the same reagents, under different conditions, to provide 15% D on both empty ring positions, 50% D on the 3‐CH_3_ ring position, and 31% D on the CH_2_ of the ethyl group (Table 11, Entry 2). These were the only reported deuterated pyrazine derivatives to the best of our knowledge.

**Table 11:** Deuteration of pyrazines via lithiation–deuteration chemistry.

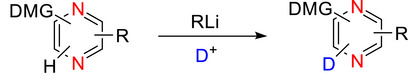						
Entry	DMG	Position(s) of H/D	R	Conditions	%Yield (% D)	Refs.
1	1,4‐N_2_	mix	2,3‐Me_2_	MeLi, Et_2_O, D_2_O, 0 °C	mixture	[198]
2	1,4‐N_2_	mix	2‐Et, 3‐Me	MeLi, Et_2_O, D_2_O, 0 °C	mixture	[198]
3	‐	C6	2‐Cl, 3‐(1,3‐dithian‐2‐yl)	*n*‐BuLi, 1,3‐dithiane, D_2_O, ‐70 °C	n.a (90)	[199]
4	‐	C3	2‐Cl, 6‐(1,3‐dithian‐2‐yl)	*n*‐BuLi, 1,3‐dithiane, D_2_O, −70 °C	n.a (70)	[199]
5	6,6′‐Cl_2_	C5, C5’	6‐Cl, 2‐(2′‐6′‐Cl‐pyrazine)	LiTMP, EtOD, −100 °C	70 (n.a)	[200]

* Primed positions refer to substituent pyridine.

Torr et al.^[^^199]^ reacted 2,3‐ and 2,6‐dichloropyrazine with 2‐lithio‐1,3‐dithiane (prepared from *n*‐BuLi and 1,3‐dithiane) and D_2_O to provide 2‐chloro‐3‐(1,3‐dithian‐2‐yl)‐6‐deuteropyrazine and 2‐chloro‐3‐deutero‐6‐(1,3‐dithian‐2‐yl)‐pyrazine in 90% and 70% D incorporation, respectively (Table 11, Entries 3–4). The reaction proceeds via nucleophilic tele‐substitution with abstraction of D from D_2_O and subsequent elimination of HCl.

Hebbar et al.^[^^200]^ performed a di‐lithiation–deuteration via D*o*M on 6,6′‐dichloro‐2,2′‐bipyrazine using LiTMP and EtOD at −100 °C. 5,5′‐Dideutero‐6,6′‐dichloro‐2,2′‐bipyrazine was produced in 70% yield, without disclosure of the extent of D incorporation (Table 11, Entry 5).

##### Deutero‐Defunctionalisation

###### Dephosphoniation

Koniarczyk et al.^[^ ^96^ ^]^ utilized K_2_CO_3_ and a D source on phosphonium salt intermediates to achieve deutero‐dephosphoniation at the 2‐position of a 5,6‐dimethylpyrazine (Table 12, Entry 1), as described previously (see section 2.1.2). Drugs and other biological compounds were similarly deuterated using this method.

**Table 12:** Monodeuteration of pyrazine via deuterodefunctionalisation.

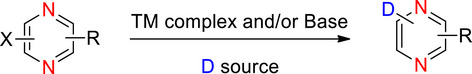					
Entry	X	R	Conditions	%Yield (% D)	Refs.
1	2‐PPh_3_^+^OTf^−^	5,6‐Me_2_	K_2_CO_3_, CD_3_OD, D_2_O, rt	80 (n/a)	[96]
2	5‐Cl	2‐N(CH_2_CH_2_)_2_NBoc	Pd(dba)_2_, K_2_CO_3_, S‐Phos, D_8_‐IPA, MeCN, 100 °C	30 (≥95)	[100]
3	2,5‐Cl_2_	‐	5% Pd/C, K_2_CO_3_, THF, D_2_O, D_2_, rt	10 (n/a)	[103]
4	3‐Cl	2‐CO_2_Me	5% Pd/C, K_2_CO_3_, THF, D_2_O, D_2_, rt	33 (n/a)	[201]

###### Dehalogenation

Donald et al.^[^ ^100^ ^]^ reported the deutero‐dechlorination of a 2‐piperazine substituted pyrazine (as the only pyrazine in the study) using a Pd(dba)_2_ catalyst, S‐Phos ligand, microwave irradiation (100 °C), with *^i^* PrOH‐d_8_ as the D source. The resulting low yields were offset by nearly quantitative D incorporation (Table 12, Entry 2).

Di‐deuteration of pyrazine via deutero‐dechlorination of dichloropyrazine was reported by Rayner et al.^[^ ^103^ ^]^ using K_2_CO_3_ and 5% Pd/C catalyst, with a low yield (Table 12, Entry 3). Unfortunately, the authors did not report the % D incorporation. It could be speculated that repeating this with the dibromo (or diiodo) ‐analogue might provide the dideuterated pyrazine in a higher yield and with greater % D incorporation.

Norcott et al.^[^ ^201^ ^]^ then synthesized a series of deuterated analogues of pyrazinamide and methyl ester pyrazine via deutero‐dechlorination using the same method, followed by transesterification or amidation using CH_3_OD  CD_3_OD (Table 12, Entry 4).

##### Transition‐Metal or Base‐Catalyzed H/D Exchange

In 1986, Breuker and colleagues^[^ ^202^ ^]^ reported the synthesis of 2,6‐dideutero‐3‐phenylpyrazine by reacting 3‐phenylpyrazine‐1‐oxide (formed by reacting phenylpyrazine with H_2_O_2_) with NaOH and 1:1 D_2_O/CD_3_OD at 40 °C (Table 13, Entry 1). The product was isolated in 80% yield and 95% D. This was the only deuterated pyrazine reported in the paper.

**Table 13:** Polydeuteration of pyrazines via HIE.

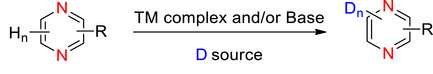					
Entry	Position(s) of H/D	R	Conditions	%Yield (% D)	Refs.
1	C2, C6	1 C = O, 3‐Ph	NaOH, 1:1 D_2_O/CD_3_OD, 40 °C	80 (95)	[202]
2	C5, C6, C3‘, C4’, C5‘, C6’, C3‘’, C4‘’, C5‘’, C6‘’	2,3‐di‐(pyrid‐2‐yl)	NaOD/D_2_O, 200 °C	90 (>98)	[133]
3	C6, C2’, C3’, C5’, C6’	2‐(4‐MeO C_6_H_4_)	[RuCl_2_(*p*‐cymene)]_2_, AgOAc, PPh3, acetic acid‐d_4_, 150 °C	89 (36 and 94)	[184]
4	C5, C6, C2,3‐(H_3_)_2_	2,3‐(CH_3_)_2_	(^Mes^CNC)Fe(CH_2_SiMe_3_)_2_(N_2_), H_2_, C_6_D_6_, 80 °C	n/a (98/98/14/14)	[138]
5	C2, C3, C5, C6‐	‐	[Ir(COD)(OMe)]_2_, THF, D_2_, 55 °C	n/a (65)	[13]

Browne et al.^[^ ^133^ ^]^ prepared perdeuterated 2,3‐di‐(pyrid‐2‐yl)‐pyrazine in 90% yield with > 98% D at all positions by H/D exchange of the nondeuterated substrate (Table 13, Entry 2). This successful deuteration was achieved using harsh conditions: the substrate was heated to 200 °C in the presence of 1 M NaOD in D_2_O.

Zhao et al.^[^ ^184^ ^]^ produced 2‐(4‐methoxyphenyl)‐6‐deuteropyrazine in 89% yield via HIE using a Ru catalyst and deuterated acetic acid (Table 13, Entry 3). At anisole ring all the positions were deuterated with 94% D incorporation and at the pyrazine ring 36% D incorporation was observed on position 6.

Corpas et al.^[^ ^138^ ^]^ reported the deuteration of aromatics and N‐heteroaromatics with excellent % deuteration through a novel pyridine dicarbene iron dialkyl catalyst (^Mes^CNC)Fe(CH_2_SiMe_3_)_2_(N_2_) and benzene‐d_6_ (D source). This bulky catalyst is thought to convert from a Fe(II) dihydride to a Fe(II) dideuteride via exchange with benzene‐d_6_, then through a classic TM catalysis mechanism with the substrate for H/D exchange (Scheme 7). 2,3‐Dimethylpyrazine‐d_4_ was produced with the 5,6‐positions almost quantitatively deuterated whereas the methyl groups were only deuterated in 14% (Table 13, Entry 4). Other electron‐poor and ‐rich aromatics and heteroaromatics were similarly deuterated. In another study, Daniel─Bertrand et al.^[^ ^13^ ^]^ synthesized perdeuterated pyrazine with 65% D incorporation using Ir catalysis and D_2_ gas (Table 13, Entry 5).

##### Deuterated Reagents

**Scheme 30:**
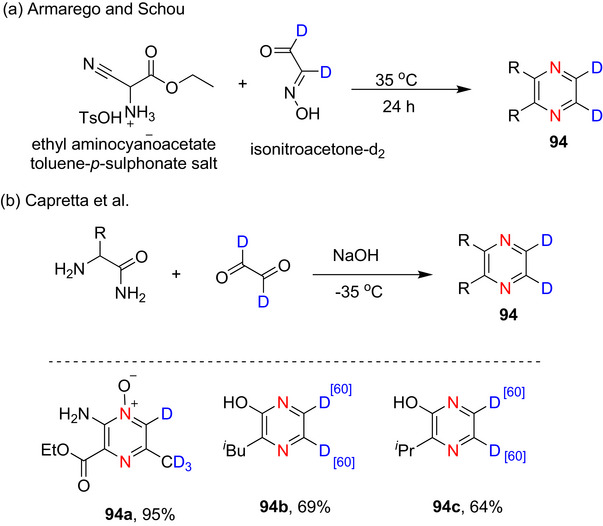
Deuterated pyrazines (**94a–94c)** synthesized by cyclization reactions of pre‐deuterated reagents (reproduced from^[^ ^204^ ^]^).

Armarego and Schou^[^ ^203^ ^]^ reported the synthesis of amino‐, cyano‐, methyl‐, and carbamoyl‐substituted pyrazines from deuterated reactants. For example, when deuterated isonitroacetone was reacted with ethyl aminocyanoacetate toluene‐*p*‐sulphonate salt at 35 °C for 24 hours, 2‐amino‐3‐(ethoxycarbonyl)‐5‐(methyl‐d_3_)pyrazine 1‐oxide‐6‐d_1_ **94a** was provided through a condensation‐cyclization reaction (Scheme 30). Similarly, 3‐isobutylpyrazin‐5,6‐d_2_‐2‐ol **94b** and 3‐isopropylpyrazin‐5,6‐d_2_‐2‐ol **94c** were prepared in 69% and 64% yield by condensation─cyclization reactions performed by Capretta et al. (Scheme 30):^[^ ^204^ ^]^ these involved the reaction of commercially available glyoxal‐d_2_ and leucinamide HCl for the isobutyl derivative, and glyoxal‐d_2_ with valinamide HCl for the isopropyl derivative.

#### 2.3.4 Fused Pyrazines

Fused pyrazines are heterocyclic compounds in which a pyrazine ring is fused with another aromatic or heteroaromatic system such as benzene, e.g., quinoxaline, **95**, pyridine, e.g., phenazine, **98**, or other heterocycles, resulting in extended conjugated structures with unique chemical and biological properties with applications e.g. in materials science^[^ ^205^, ^206^, ^207^ ^]^ and medicinal chemistry.^[^ ^208^, ^209^ ^]^

##### Quinoxaline

**Figure 5:**
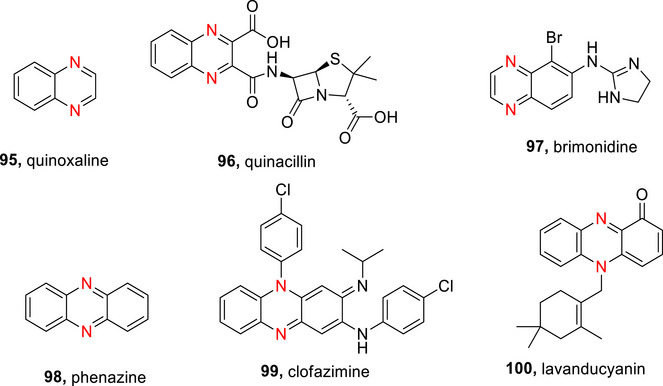
Chemical structures of quinoxaline **95** and phenazine **98** along with some examples of fused pyrazine pharma compounds quinacillin **96**, brimonidine **97**, clofazimine **99**, and lavanducyanin **100**.

Most quinoxaline (**95**, Figure 5) derivatives are synthetic and natural quinoxaline derivatives are rare.^[^ ^210^ ^]^ Quinoxalines have received a significant amount of attention due to their potential use in fighting various pathophysiological conditions like epilepsy, Parkinson's, and Alzheimer's diseases.^[^ ^211^ ^]^ They are also used in the treatment of bacterial, cancer, and HIV infections.^[^ ^212^ ^]^ Structurally, the 1,4 disposition of the two imine nitrogen atoms in one of the rings of the quinoxaline bestows it with electron‐withdrawing character.^[^ ^213^ ^]^

Quinoxaline and phthalazines are isosteric to quinolines since they contain one more nitrogen atom. Quinacillin (**96**, Figure 5) is a quinoxaline‐containing semisynthetic penicillase‐resistant penicillin with antibacterial activity;^[^ ^214^ ^]^ it binds to and inactivates penicillin‐binding proteins (PBPs) located on the inner membrane of the bacterial cell wall.

**Scheme 31:**
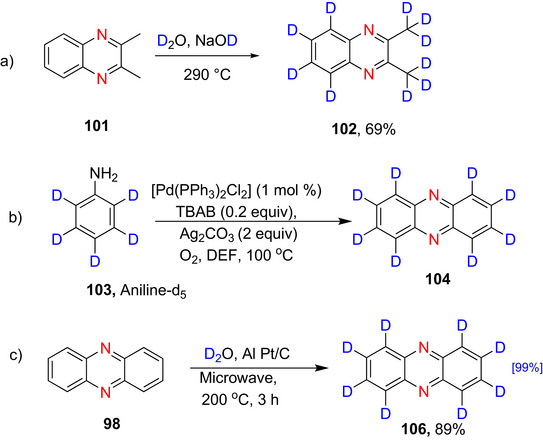
a) Perdeuteration of 2,3‐dimethylquinoxaline **101**.^[^ ^215^ ^]^ b) Synthesis of perdeuterated phenazine **104** from aniline‐d*_5_* **103**.^[^ ^216^ ^]^ c) Microwave assisted synthesis of perdeuterated phenazine **104**.^[^ ^217^ ^]^

The synthesis of perdeuterated 2,3‐dimethylquinoxaline (Scheme 31a) was reported in 1997 by Junk et al.^[^ ^215^ ^]^ in a moderate yield (69%). The % D was not reported for **102**.

There are no published reports on the selective deuteration of fused pyrazines to the best of our knowledge.

##### Phenazine

Phenazines (**98**, Figure 5) are heterocyclic, nitrogenous compounds (essentially, dibenzo annulated pyrazines). They are natural products typically obtained from bacteria,^[^ ^218^ ^]^ but due to an expanding repertoire of applications, have attracted attention in the development of their chemical synthesis.^[^ ^219^ ^]^ Examples include Clofazimine (**99**, Figure 5) which has been used to treat dapsone‐resistant leprosy, psoriasis, pyoderma gangrenosum, discoid lupus, *Mycobacterium avium*‐complex infections in AIDS patients^[^ ^220^ ^]^ and functional materials, e.g., biosensors^[^ ^213^ ^]^ and fluorescent OLED emitters.^[^ ^214^ ^]^ Deuterated phenazines have also been reported. Seth et al.^[^ ^216^ ^]^ demonstrated the synthesis of perdeuterated phenazine from aniline‐d*_5_*, **83** using [PdCl_2_(PPh_3_)_2_] as the catalyst (Scheme 31b). In another report, Shimodaira et al.^[^ ^217^ ^]^ demonstrated the microwave‐assisted perdeuteration of phenazine using Pt‐activated C (5%), aluminum powder, and D_2_O as the deuterium source (Scheme 31c).

#### 2.3.5 Pyridazine

**Scheme 32:**
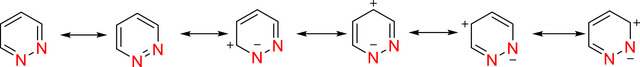
The resonance structures of pyridazine.^[^ ^69^ ^]^

Pyridazine is a six‐membered 1,2‐diazine system with two adjacent nitrogen atoms (Scheme 32). The basicity of the parent compound (p*K*_a_ = 2.3) is considerably lower than that of pyridine (p*K*_a_ = 5.2) but higher compared to pyrimidine (p*K*_a_ = 1.3) and pyrazine (p*K*_a_ = 0.6). The reactivity of the pyridazine can be understood from the two neutral resonances structures shown in Scheme 32.^[^ ^69^ ^]^

It is evident that, unlike pyridine or pyrimidine, the pyridazine molecule does not have any “unactivated” carbon on the aromatic ring with respect to nucleophilic attack. Therefore, nucleophilic substitution reactions play a prominent role within the synthetic methods used for modification of pyridazines. Furthermore, the preferred site of nucleophilic attack at the unsubstituted pyridazine ring is at C4/C5 rather than the carbon next to a ring nitrogen. This can be observed in Chichibabin type aminations substitution reactions with nucleophilic carbon‐centered radicals, or with addition reaction of Grignard reagents.^[^ ^223^ ^]^ On the other hand, alkyl‐ and aryllithium reagents preferentially add across C3/N2. The electron‐deficient character of the pyridazine ring also makes it susceptible toward (partial) reduction, e.g., with LiAlH_4_, in which case electron‐withdrawing substituents further decrease the electron density. In general, the reaction behavior strongly depends on the presence and nature of substituents, e.g. electrophilic substitution reactions can be performed with pyridazines bearing (preferably at least two) electron‐donating groups or with various pyridazine N‐oxide derivatives.

##### Lithiation/Zincation–Deuteration: Metalation

In 2019, Balkenhohl et al.^[^ ^224^ ^]^ employed D*o*M and directed *meta*‐metalation (D*m*M) in the lithiation‐deuteration of 3‐phenylpyridazine and pyridazine (Table 14, Entries 1–3). In this report, D*m*M occurs strictly due to steric effects of a bidentate Lewis acid adduct. For example, the deuteration of 3‐phenylpyridazine was achieved using LiTMP and D_2_O under classic D*o*M conditions that facilitate coordination of the less sterically hindered ring nitrogen by the lithium amide. These conditions resulted in an excellent yield and D incorporation (Table 14, Entry 1). Although technically a directed *ortho*‐zincation rather than a lithiation, Balkenhohl et al. then employed BF_3_·OEt_2_ to coordinate with one of the N atoms on pyridazine, acting as a DMG and increasing the acidity of the H in the 3‐position. TMP‐ZnCl·LiCl is then used to deprotonate‐metalate at the 3‐position with a D_2_O quench for 33% D incorporation (Table 14, Entry 2). In another reaction, the group then used the Lewis acid 5,10‐dichloro‐5,10‐dihydroboranthrene to coordinate the B atom to each ring nitrogen on pyridazine and increase the acidity of the H atoms on pyridazine. In this scenario, this sterically hindered bidentate adduct allows for D*m*M at the 4‐position with 45% D incorporation after D_2_O quench (Table 14, Entry 3).

**Table 14:** Monodeuteration of pyridazine via D*o*M and D*m*M.

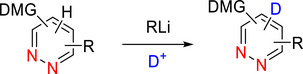						
Entry	DMG	Position(s) of H/D	R	Conditions	%Yield (% D)	Refs.
1	N1	C6	3‐Ph	LiTMP, THF, D_2_O, −78 °C	93 (85)	[224]
2	2‐BF_3_·OEt_2_	C3	‐	BF_3_·OEt_2_, THF, 0 °C, TMPZnCl·LiCl, 25 °C, D_2_O	n/a (33)	[224]
3	1,2‐bidentate group	C4	‐	5,10‐Dichloro‐5,10‐dihydroboranthrene, THF, TMPZnCl·LiCl, −78 °C, D_2_O	n/a (45)	[224]

##### Deutero defunctionalisation

Deuteration of pyridazines has only been accomplished via deuterodehalogenation, described as follows.

###### Dehalogenation

Leprêtre et al.^[^ ^225^ ^]^ reported the deutero‐dehalogenation of 4‐iodo‐3,6‐dimethoxypyridazine and 4,5‐dibromo‐3,6‐dimethoxypyridazine through the formation of Grignard reagents and subsequent quenching with DCl/EtOD at ambient temperatures (Table 15, Entry 1–2). Good yields were achieved with a range of Grignard reagents, including *n*‐Bu_2_Mg and *i*‐PrMgCl, although no % D was reported.

**Table 15:** Monodeuteration of pyridazines via deutero‐dehalogenation.

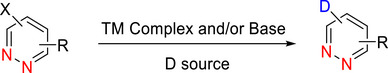					
Entry	X (D)	R	Conditions	%Yield (% D)	Refs.
1	4‐I	3,6‐OMe_2_	*n*‐Bu_2_Mg, THF, DCl, EtOD, 20 °C	72 (n/a)	[225]
2	5‐Br	4‐Br, 3,6‐OMe_2_	*i*‐PrMgCl, EtOD, 20 °C	80 (n/a)	[225]
3	3‐Cl	6‐Me	5% Pd/C, K_2_CO_3_, EtOD, D_2_, 20 °C	80 (n/a)	[226]
4	3‐Cl	6‐CD_3_	5% Pd/C, K_2_CO_3_, EtOD, D_2_, 20 °C	74 (n/a)	[226]
5	3,5‐Cl_2_	‐	5% Pd/C, K_2_CO_3_, THF, D_2_O, D_2_, 20 °C	44 (n/a)	[103]

Using a different strategy, Roy et al.^[^ ^226^ ^]^ employed heterogeneous Pd‐catalyzed deutero‐dechlorination on a range of pyridazines, including 3‐chloro‐6‐methylpyridazine and 3‐chloro‐6‐ methylpyridazine‐d_3_. The deuterated products 3‐methylpyridazine‐d_1_ and methylpyridazine‐d_4_ were produced in good yield, again without any mention of the percent D incorporation (Table 15, Entry 3–4). Rayner^[^ ^103^ ^]^ then reported the same procedure on 3,5‐dichloropyridazine, producing 3,5‐pyridazine‐d_2_ in moderate yield, using D_2_O as the D source (Table 15, Entry 5).

##### Transition‐Metal or Base‐catalyzed HIE

In 1969, Zoltewicz et al.^[^ ^187^ ^]^ reported the preparation of perdeuterated pyridazine in moderate yield and high (81%) D incorporation by reacting pyridazine with CH_3_ONa and CH_3_OD under high temperatures (165 °C) (Table 16, Entry 1). Following this, Crossland et al.^[^^227]^ found that heating 3,6‐dimethoxypyridazine with NaOMe and CH_3_OD at 100 °C formed the dideuterated analogue with excellent (92%) D incorporation but in unknown yield (Table 16, Entry 2).

**Table 16:** Polydeuteration of pyridazines via HIE.

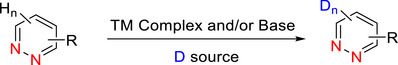					
Entry	H	R	Conditions	%Yield (% D)	Refs.
1	C3, C4, C5, C6	‐	CH_3_ONa, CH_3_OD, 165 °C	43 (81)	[187]
2	C4, C5	3,6‐(OMe)_2_	NaOMe, CH_3_OD, 100 °C	n/a (92)	[227]
3	C4	‐	CuI/Phenanthroline, *t*‐BuOD, *t*‐BuOLi, DMPU, 125 °C	n/a (35)	[228]

In 2008, Do et al.^[228]^ formed a mixture of 4‐deuteropyridazine and 4,5‐dideuteropyridazine via HIE using a CuI/phenanthroline catalyst, N,N’‐dimethylpropyleneurea (DMPU) solvent, *t*‐BuOD as D source, and lithium tert‐butoxide (*t‐*BuOLi) as base (Table 16, Entry 3). The yield of the deuterated product was unknown while the % D incorporation was moderate (35%). Using weaker t‐BuOLi as base (cf. KOtBu) ensured arylation regioselectivity by curbing the benzyne pathway. Furthermore, the researchers found that using a phenanthroline ligand furthermore allows base *t*‐BuOLi to be used for a less acidic heterocycle reaction (such as with pyridazines).

Esselman et al.^[^ ^229^ ^]^ employed the same procedure by Do et al.^[^ ^228^ ^]^ to deuterate pyridazine, resulting in a mixture of nine deuterated pyridazine species: pyridazine‐3‐d_1_, pyridazine‐4‐d_1_, pyridazine‐3,4‐d_2_, pyridazine‐3,5‐d_2_, pyridazine‐3,6‐d_2_, pyridazine‐4,5‐d_2_, pyridazine‐3,4,5‐d_3_, pyridazine‐3,4,6‐d_3_, pyridazine‐3,4,5,6‐d_4_. However, neither the yields nor the % D incorporation percent of any of the species was reported.

Finally, Pfeifer et al.^[^ ^230^ ^]^ observed non directed deuteration on 6‐methylpyridazin‐3‐ol using Pd NPs with D_2_ gas. The percentage of D incorporation was only 35% at the substituted methyl group and an unexpected deuteration was observed on the two aromatic positions of the 6‐membered heterocyclic ring.he efficiency and the obtained isotopic enrichment with the palladium catalyst goes hand in hand with the strength of the benzylic character of a targeted position. If the aromatic scaffold is different to benzene, the benzylic character of adjacent C(sp^[^ ^3^ ^]^)‐atoms is affected. Thus, the C─H bonds of the latter can be less easily activated by palladium.

#### 2.3.6 Fused Pyridazines

##### Phthalazine

**Scheme 33:**
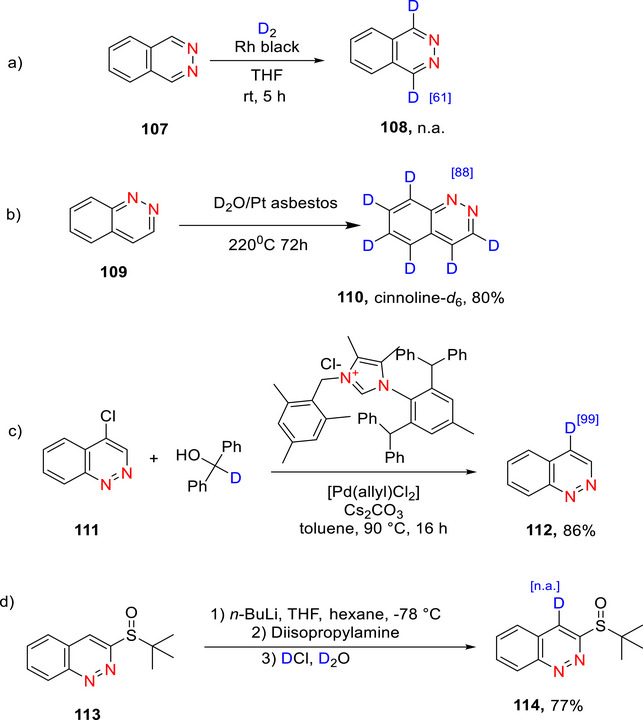
a) Deuteration of phthalazine **107** by Alexakis et al.^[^ ^119^ ^]^ b) Deuteration of cinnoline **106** by Gad Fischer^[^ ^130^ ^]^ c) Deuteration of 4‐chlorocinnoline **111** by Kuriyama et al.^[^ ^236^ ^]^ d) Deuteration of 3‐(tert‐butylsulfinyl)cinnoline **113 by** Le Fur et al.^[^ ^237^ ^]^

Phthalazine (**107**, Scheme 33a), also called benzo‐*ortho*diazine or benzopyridazine, is isomeric with naphthyridines such as quinoxaline and quinazoline. Phthalazine derivatives, like the other members of the isomeric benzodiazine series, have been investigated as therapeutic agents due to their antimicrobial, antiepileptic, cardiotonic, vasorelaxant, anti‐inflammatory and analgesic properties^[^ ^231^, ^232^ ^]^ and in OLEDs.^[^ ^233^, ^234^ ^]^

The only reported example of deuteration of phthalazines was reported by Alexakis and collaborators,^[^ ^119^ ^]^ in which they accomplished selective deuteration at positions 1 and 4 of phthalazine using D_2_ gas and a heterogeneous rhodium catalyst with high % incorporation D (compound **78,** Scheme 21).

##### Cinnoline

Cinnoline (**109**, Scheme 33b) is one of the two benzo derivatives of pyridazines (phthalazine is the other). Cinnolines are pharmaceutically and biologically important structures with anticancer, antimicrobial, anti‐inflammatory, antiparasitic, and foliar herbicide activities.^[^ ^235^ ^]^

In 1973, Gad, Fischer and Puza reported the synthesis of D‐cinnoline **110** by simply heating cinnoline **109** with D_2_O (27 equiv.) over platinized asbestos in a sealed tube at 170 ⁰C for 24 h and 220 ⁰C for 88 h (Scheme 33b).^[^ ^130^ ^]^

Kuriyama et al.^[^ ^236^ ^]^ described a deuterodechlorination of cinnoline at position C4 (see compound (**112**) catalyzed by a Pd/unsymmetrical NHC system starting from the corresponding chloride (**111**, Scheme 33c), using α‐deuterioalcohols as deuterating agents, which was synthesized by deuterating benzophenone/acetophenone using lithium aluminum deuteride in THF. It was found that diphenylmethane‐ol‐d_1_ was the most efficient in terms of % D incorporation (99%).

Fur et al.^[^ ^237^ ^]^ reported on the metalation of 3‐tert‐butylsulfinylcinnoline and subsequent deuteration at position 4 (**114**). It was demonstrated that the sulfoxide group was a powerful *ortho*‐directing group (see **113**, Scheme 33d). The electrophilic D source was DCl in D_2_O (D_2_O acted as a solvent). Interestingly, the reaction of 2‐[(tert‐butyl)sulfinyl]quinoxaline did not yield the desired products. Regrettably, the % D incorporation was not reported.

### 2.4 Triazines

**Scheme 34:**
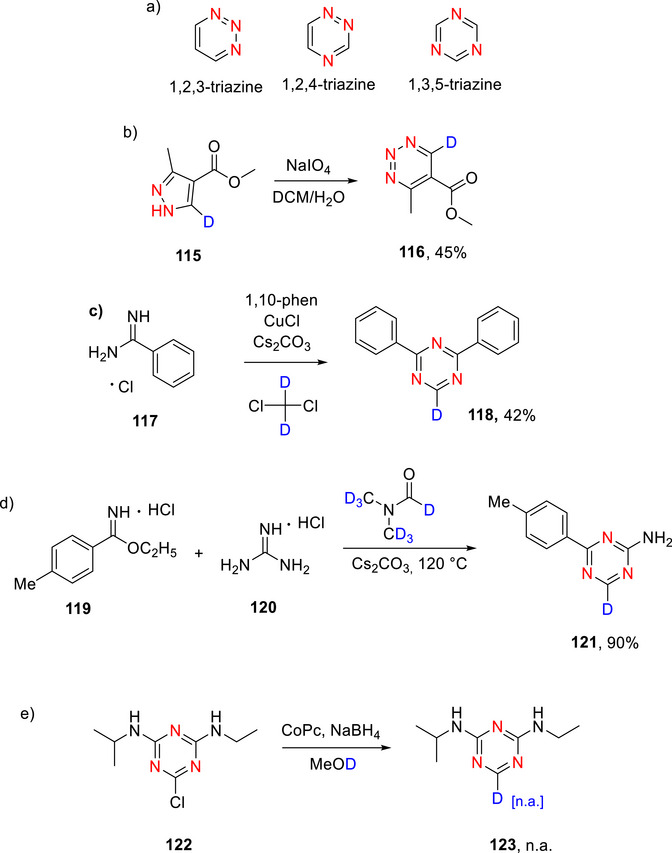
a) The three isomeric forms of triazines. b) Synthesis of deuterated triazine **116** by oxidative ring expansion.^[^ ^243^ ^]^ c) Synthesis of monodeuterated triazine **118**.^[^ ^245^ ^]^ d) Base‐mediated three‐component Reaction to obtain deuterated 1,3,5‐Triazin‐2‐amines **121**.^[^ ^246^ ^]^ e) Synthesis of a deuterated 1,3,5‐triazine‐derivative **123** via deuterodehalogenation.^[^ ^247^ ^]^

Triazine isomers and their derivatives are known to play important roles in medicinal and agricultural fields^[^ ^238^ ^]^ as well as in organic electronic devices.^[^^239–241]^Triazines exhibit similar reactivity to the diazines. Triazine is a six‐membered heterocyclic ring containing three Ns, replacing three of the C─H bonds in the benzene ring. Based on the N position present in the ring system, it is categorized in three isomeric forms (see Scheme 34a), i.e., 1,2,3‐triazine (vicinal triazine), 1,2,4‐triazine (asymmetrical triazine or isotriazine) and 1,3,5‐triazine (symmetrical or s‐triazine or cyanidine). Additionally, triazines are weak bases. The inductive effects of the Ns means that the triazines (in comparison to the diazines) are more susceptible to nucleophilic substitution than electrophilic substitution.^[^ ^242^ ^]^ Deuterated triazines have reportedly been prepared either from deuterated substrates / reagents / solvents or via deuterodehalogenation.

#### 2.4.1 Preparation From Deuterated Substrates/Reagents

The preparation of deuterated 1, 2, 3‐triazines was reported by Quiñones et al.^[^ ^243^, ^244^ ^]^ in the form of methyl 4‐methyl‐1,2,3‐triazine‐5‐carboxylate‐6‐d_1_ (116) (Scheme 34b). This compound was, prepared from methyl 3‐methyl‐1*H*‐pyrazole‐4‐carboxylate‐5‐d (115) by the oxidative ring expansion of *N*‐aminopyrazoles. The reaction was initiated by NaIO_4_ in a biphasic reaction solution (yield: 58%) with 45% D incorporation of the product 116.

The formation of deuterated 1,3,5‐triazine (compound **118**) starting from amidine **117** was accomplished by Yan et al.^[^ ^245^ ^]^ by using 1,10‐phenanthroline as ligand, cuprous chloride as catalyst and Cs_2_CO_3_ as a base. Deuterated DCM was used as source of D (yield: 42%, Scheme 34c). The % D incorporation of the product 97 was not reported.

A base‐mediated three‐component reaction of imidate **119**, guanidine **120**, and deuterated DMF was reported to provide unsymmetrical 1,3,5‐triazin‐2‐amine‐d_1_ (121, Scheme 34d)^[^ ^246^ ^]^ in an excellent 90% yield. Although this was prepared to probe the mechanism of the reaction, it is nevertheless a useful approach to form a deuterated triazine despite the poor atom economy with respect to the D atoms.

#### 2.4.2 Deuterodehalogenation

A reductive deuterodechlorination of atrazine (**122**), an herbicide possessing the core 1, 3, 5‐triazine), has been reported by Poonam and collaborators^[^ ^247^ ^]^ (Scheme 34e). They used cobalt phthalocyanine (CoPc) as catalyst and MeOD as source of D in the presence of NaBH_4_. Regrettably, neither the yield nor the % D incorporation of **123** were reported (the researchers were trying to understand the mechanism, also reporting that the reaction of atrazine with sodium borodeuteride (NaBD_4_) in methanol (CH_3_OH) in the presence of CoPc only gave the protonated analogue of **123**).

### 2.5 Tetrazines

**Figure 6:**
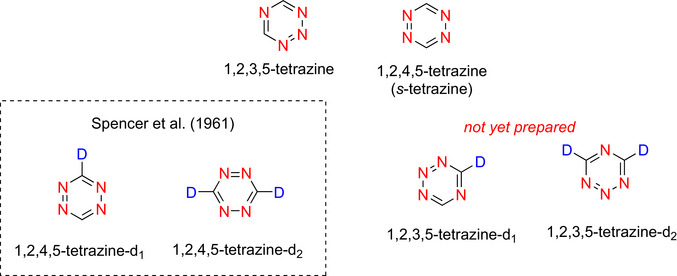
Tetrazines and their reported deuterated derivatives.

Tetrazines (1,2,3,5, as well as the more common 1,2,4,5‑tetrazines or *s*‐tetrazines) are six‑membered, nitrogen‑rich aromatic heterocycles known for being strongly electron‑poor dienes (Figure 6). They are well known for their applications such as biomedical applications,^[^^248]^ biorthogonal chemistry,^[^^249]^ and functional materials.^[^^250]^ There are only two known deuterated tetrazines. In 1961, Spencer et al.^[^^251^^]^ reported the synthesis of deuterated tetrazines (*s*‐tetrazine‐d_1_ and ‐d_2_) (Figure 6). The synthesis was carried out through the pyrolytic decarboxylation of 3,6‐disubstituted *s*‐tetrazine dicarboxylic acid following partial H/D exchange in D_2_O. However, this exchange process was notably inefficient due to its heterogeneous nature and the intrinsic instability of the hydrated diacid, which is prone to spontaneous decarboxylation, rearrangement, or decomposition. The highest yield reported for *s*‐tetrazine‐d_1_ using this method was 52%, accompanied by 40% s‐tetrazine and 8% s‐tetrazine‐d_2_. Efforts to obtain pure *s*‐tetrazine‐d_1_ via the synthesis of a monocarboxylic acid intermediate proved unsuccessful. Thus, the selective preparation of isotopically labeled s‐tetrazines remains a synthetic challenge. There are also reports of deuterated tetrazines that are used for analytical/physical studies, without any indication of how they were synthesized.^[^^252,253]^ 1,2,3,5‐tetrazine‐d_1_ and d_2_ have not yet been reported.

### 2.6 Fused Triazines and Tetrazines

At the time of publication of this review, there were no reports on deuterated fused triazines or tetrazines in the literature. This presents a literature gap worth exploring, given the applications of these materials.

## 3 Deuteration of compounds containing more than one type of 6NHetAr covalently connected

**Figure 7:**
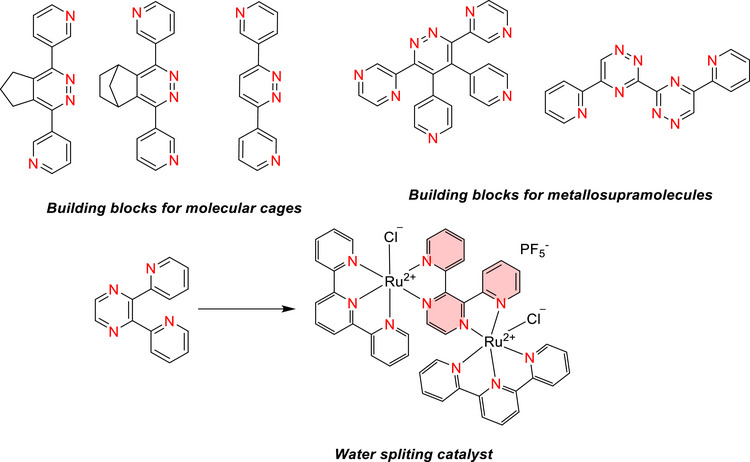
Examples of pharma compounds and functional materials containing more than one type of 6NHetAr covalently connected.^[^ ^254^, ^255^, ^256^, ^257^ ^]^

The combinatorial possibilities of connecting 6NHetArs with other 6NHetArs opens possibilities for the modular synthesis of a range of compounds, and, indeed, there are many examples of medicinal and functional materials with more than one 6NHetAr connected via covalent bonds. Some of these^[^ ^254^, ^255^, ^256^, ^257^ ^]^ are shown in Figure 7 below.

When preparing the deuterated variants of these compounds, it is therefore necessary to decide whether to use pre‐deuterated substrates and build up the combined NHetAr compounds or to attempt to deuterate the overall compound via late‐stage deuteration. This also depends on whether perdeuteration or selective mono‐ or poly‐deuteration (and the position of the deuteration) is desired.

**Scheme 35:**
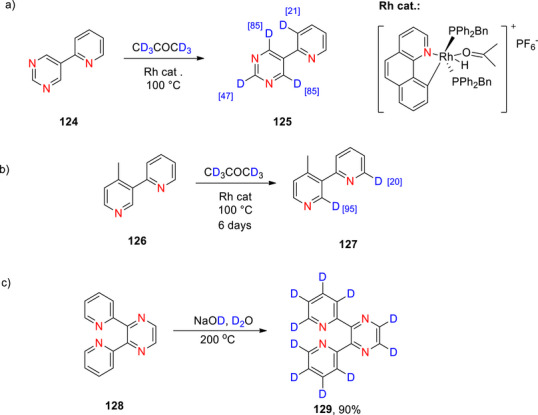
a) Deuteration of 5‐(pyridine‐2‐yl)pyrimidine **124** via rhodium catalyst.^[^ ^258^ ^]^ b) Deuteration of 4′‐methyl‐2,3′‐bipyridine **126**.^[^ ^258^ ^]^ c) Perdeuteration of 2,3‐bis(pyrid‐2′‐yl)‐pyrazine **129**.^[^ ^133^ ^]^

For example, three C─H bonds were exchanged for C─D bonds in 5‐(pyridine‐2‐yl)pyrimidine 124 ^[^^258]^ (Scheme 35a). In this case, late‐stage deuteration of the molecule was demonstrated with three positions on the pyrimidine ring (C‐2, C‐4 and C‐6) as well as the *ortho* position to the nitrogen of pyridine, but with lower % D incorporation (47%). To achieve this, 2‐propanone‐1,1,1,3,3,3‐d_6_ was used as the source of D and rhodium as the catalyst (Rh cat, Scheme 35). The substrate, catalyst, and D source were stirred 100 °C for 9 days to afford 5‐(pyridine‐2‐yl‐3‐d) pyrimidine‐2,4,6‐d_3_ (125, Scheme 35a). 4′‐methyl‐2,3′‐bipyridine **126** was also screened as a substrate; deuteration afforded 4′‐methyl‐2,3′‐bipyridine‐2′,6‐d_2_ (127, Scheme 35b). % D incorporation on the two positions adjacent to the N (2′ and 6) was 13% and 67% after 2 days, whereas after 6 days it increased to 20% and 95%.

In a third example, Browne et al.^[^^133]^ reported the perdeuteration of 2,3‐bis(pyrid‐2′‐yl)‐pyrazine **128** (Scheme 35c). The substrate **128** was treated with 1 M NaOD/D_2_O at 200 °C to obtain 2,3‐di‐(pyrid‐2yl)‐pyrazine‐d_10_ (**129**) with 98% D incorporation and 90% yield.

This short section is not comprehensive but rather meant to give a snapshot into the possibilities of preparing deuterated analogues of compounds containing more than one type of 6NHetAr covalently connected (deuterated at the 6NHetAr).

## 4 Deuteration of a wide range of N‐heteroarenes With a single protocol

In this section, we highlight several studies in which a singular methodology or scientific protocol can be used for the deuteration of a wide range of N‐heteroaromatic substrates. This approach is useful when it is possible to employ one method to deuterate a range of different substrates (thus, simplifying the reagent requirement if a range of separate substrates needs to be deuterated). However, it cannot be used in instances where selective deuteration is required within a more complex compound consisting of several NHetAr moieties, posing the risk of over‐deuteration (see Section 3).

**Scheme 36:**
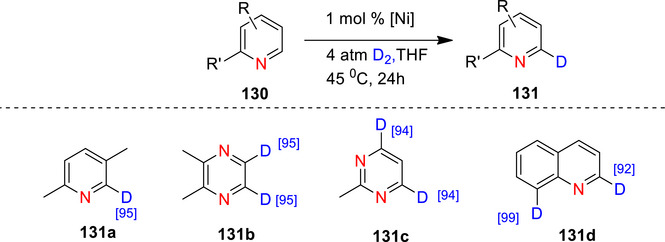
Ni‐Catalyzed Deuteration of various 6NHetAr (**131a–131d)** (from Yang et al.^[^ ^259^ ^]^).

In the first of a few examples to demonstrate this, Yang et al.^[^^259]^ demonstrated nickel‐catalyzed site selective deuteration of 6NHetAr. The Ni catalyst was either an α‐diimine nickel hydride complex (a single component precatalyst) or it was generated in situ from readily available and air‐stable metal and ligand precursors (iPrDI,[(NEt_3_)Ni(OPiv)_2_]_2_ (Piv = pivaloyl) and (EtO)_3_SiH). The H/D exchange process involved 1 mol% of Ni complex (utilizing either isolated or in situ generated precatalyst), with D_2_ gas the D‐source. The isolated form of Ni resulted in good deuteration compared to the in situ‐generated complex (Scheme 36).

**Scheme 37:**
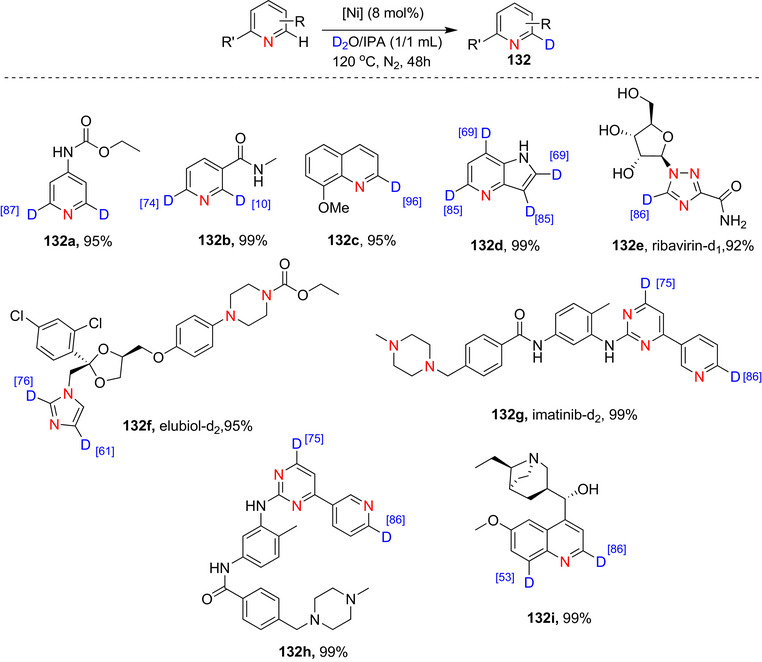
Deuteration of *N*‐heterocycle (**132a–132d**) and drugs molecules (**132e–132i**), demonstrated by Qiu et al.^[^ ^260^ ^]^

Qiu et al.^[^^260]^ reported a scalable and selective hydrogen isotope exchange on 6NHetAr using nickel NPs, Ni‐bpy@γ‐Al_2_O_3_ (bpy = bipyridine) supported on γ‐Al_2_O_3_ as the catalyst, with D_2_O as the deuterium source. This system was successful in deuterating several 6NHetAr and pharmaceuticals containing multiple NHetAr in one molecule with reasonably % D (Scheme 37). Notably, the process could be used to deuterate complex molecules both on a small scale, e.g., **132f** and **132g**, as well as on a 10 g scale, e.g., **132h** and **132i**.

**Scheme 38:**
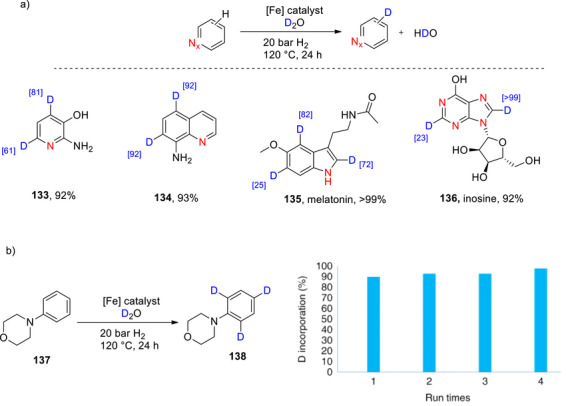
a) Deuteration of various 6NHetAr **135**–**136**, and b) catalyst recyclability study involving the deuteration of 4‐phenylmorpholine **137** on a gram scale using the strategy of Li et al.^[^ ^165^ ^]^ Reproduced with permission under the terms of the Creative Commons CC BY license.

Li et al. ^[^^165]^ demonstrated that a nanostructured iron catalyst, prepared by reacting cellulose with iron salts, which facilitated the selective deuteration of several (hetero)arenes (Scheme 38) using D_2_O as source of D. The authors proposed a concerted mechanism in which D_2_O is split homogeneously by the iron catalyst, yet the resulting radicals are not liberated into the solution but remain adsorbed on the catalyst surface as activated D* and *OD species. The N‐heteroaromatics that were deuterated using this methodology include e.g., pyridines e.g., 133 and quinolines e.g., **134**   (Scheme 38).

This study also demonstrated that the iron catalyst was recycled up to five times, as shown with 4‐phenylmorpholine **137** (also performed on a large gram scale). The authors also reported the preparation of deuterated bioactive molecules: most of the deuterated scaffolds contained pyridine, indole or purine cores. Notably position C8 in purines was efficiently deuterated; melatonin‐d_3_ (**135**) was formed, and inosine was converted to its corresponding deuterated counterpart **136** with an excellent % D (99%).

**Scheme 39:**
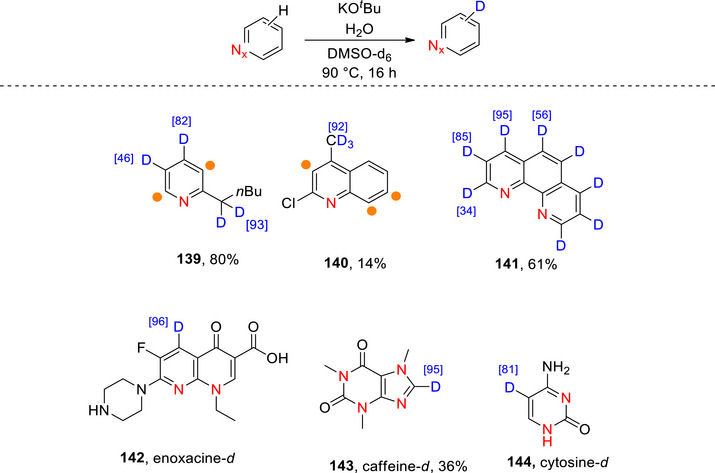
Deuteration of 6NHetAr **139**–**141** and *N*‐containing drugs/bioactive molecules (**142**–**144**) using the strategy of Kopf et al.^[^ ^145^ ^]^ Orange dot indicates sites of minor deuteration (% of D < 25).

Kopf et al. ^[^^145]^ described the remote deuteration of pyridines and other azines (see compounds **139–141**, Scheme 39). The chemistry involves a base‐mediated deuteration process using a mix of KO*^t^* Bu and DMSO‐d_6_ (Scheme 39).

The euthors pointed out that halogenated substrates afforded a range of side products and therefore lower yields, presumably resulting from competing halogen transfer reactions under the basic reaction conditions.

The production of deuterated bioactive compounds (**142–144**) was reported by the same authors (Scheme 39). In detail, the *para* position on the pyridine was deuterated with moderate % D (25%). On the other hand, position C8 in caffeine **143** was efficiently deuterated (95% D incorporation).

**Figure 8:**
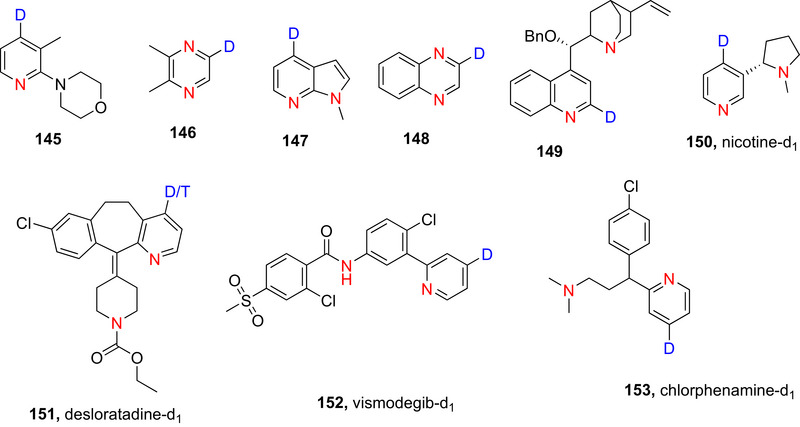
Pyridine **145**, fused pyridine **149**, pyrazine **146** (and fused pyrazine **148**), and a few representative *N*‐containing drugs/bioactive molecules (**150**–**153**) that were deuterated using the methodology by Koniarczyk et al.^[^ ^96^ ^]^

As shown in the previous sections (see Scheme 4), Koniarczyk et al.^[96]^ deuterium isotopes are incorporated in high yields and, in general, with exclusive regioselectivity. Deuterated pyridines are shown in Figure 8. A range of monosubstituted as well as 2,3‐ and 2,5‐disubstituted pyridines containing a diverse array of functional groups are effective.

Other heterocycles such as pyrimidines, pyrazines and azaindoles, performed well in this protocol. In Figure 8, the deuterated products are shown.

The phosphonium salt formation−deuteration strategy is also effective for aza arene‐containing drugs. Some examples are given in Figure 8. Pyridine‐containing drugs e.g., nicotine **150**, desloratadine **151**, vismodegib **152**, and chlorphenamine **153**, were effectively deuterated at the C‐4 of the pyridine ring.

**Scheme 40:**
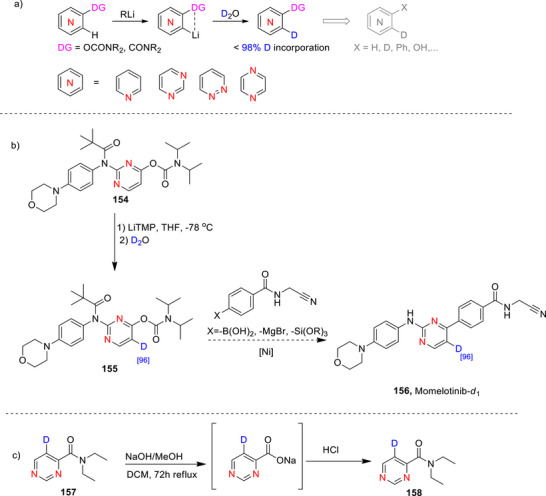
a) TM‐free *ortho*‐deuteration of electron‐deficient N‐heteroarenes, as demonstrated by Oražem et al.^[^ ^261^ ^]^ b) Synthesis of the mono‐deuterated analogue of the precursor to anticancer drug momelotinib (**156**) using the above strategy. c) Hydrolysis of deuterated *N*,*N*‐diethylpyrimidine‐4‐carboxamide (**158**) reported by Oražem et al.^[^ ^261^ ^]^

Oražem et al.^[^ ^261^ ^]^ recently demonstrated the use of TM‐free direct for the selective deuteration of a range of electron‐deficient N‐heteroarenes. O‐carbamate and carboxamide directing groups (DGs) were used to direct the lithiation, using LiTMP was the preferred base and D_2_O as a green source of electrophilic D (D^+^) (Scheme 40a). Mechanistically, the process is influenced not only by the coordination of the DG to lithium but also by the intrinsic electronic effects of the nitrogen atoms in the heteroarene, which control the relative acidity of different C─H bonds. This method provides high isotopic incorporation (up to 98%) with excellent site‐selectivity but low yields for some of the DG‐functionalized 6NHetAr due to decomposition and formation of side products. Furthermore, the DGs themselves are synthetically versatile: following D installation, they can be transformed into other functional groups or used in cross‐coupling reactions, making the method modular and applicable to the late‐stage preparation of complex molecules, as exemplified by the synthesis of precursor **154** to momeletinib‐d_1_ (**156**) (Scheme 40b). As a proof of concept to show that deuterated amides could be hydrolysed to the corresponding deuterated carboxylic acids (a more versatile functional group in further transformations, the hydrolysis of deuterated N,N‐diethylpyrimidine‐4‐carboxamide (**157**) to deuteratedpyrimidine‐4‐carboxylic acid (**158**) was demonstrated Scheme 40c.

A final example involves the use of continuous flow chemistry for the perdeuteration of a range of N‐heteroaromatic compounds.^[^ ^141^ ^]^ The method used D_2_O as source of D and the reaction was catalyzed by a heterogeneous catalyst consisting of defective porous carbon material generated by pyrolysis of cellulose, a biopolymer. Pyridines, bipyridines, phenylpyridines, quinolines, indoles, and a 9*H*‐pyrido[3,4‐*b*]indole were perdeuterated with varying degrees of deuteration at each C─H position.

## 5 Applications of deuterated N‐Containing heteroaromatics

### 5.1 Pharmaceutical Compounds: Therapeutics and Internal Standards

Deuterated N‐containing heteroaromatic compounds have gained prominence in pharmaceutical sciences due to their enhanced metabolic stability, prolonged half‐life, and improved safety profiles. These isotopologues are used not only as active pharmaceutical ingredients but also as internal standards in bioanalytical assays due to their chemical similarity and distinguishability via mass spectrometry. Table 17 highlights notable examples used as therapeutics.

**Table 17:** Notable examples of deuterated therapeutics.

Entry	Deuterated drug	Commercially approved	Use	Refs.
1	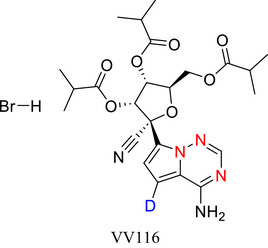	Approved	Antiviral Drug	[262]
2	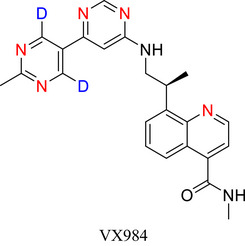	Phase I completed	Advanced solid tumors	[263]
3	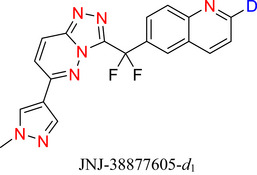	Not Approved	Cancer	[41]

### 5.2 Functional Materials Containing Deuterated N‐Heteroarenes

Besides pharmaceuticals, deuterated 6NHetAr compounds play an important role in enhancing the properties of functional materials used in a range of applications such as organic electronic materials, imaging dyes, and catalysts. The deuterated analogues of these materials are also useful as contrast agents for neutron experiments, enabling the elucidation of the morphology and structural conformations of materials. We now highlight select cases of each of these.

#### 5.2.1 Luminescent Materials Containing D‐HetAr

Materials that emit light in the form of fluorescence (emission from an excited molecule with retention of spin multiplicity) or phosphorescence (emission accompanied by a change in spin identity) are useful in a range of applications such as medical and diagnostic imaging, detection and sensing, photocatalysis, and in light displays.^[^ ^264^, ^265^ ^]^ Many of these materials are *N*‐heteroaromatic, occurring as ligands of metal complexes and electron or hole transport materials in OLEDs. For example, pyridines, pyrimidines, triazines, and phenanthrolines are typically used as electron transport materials (ETMs) as they can provide high electron mobilities and bandgap tunability.^[^ ^266^ ^]^

##### Luminescent Metal Complexes

**Scheme 41:**
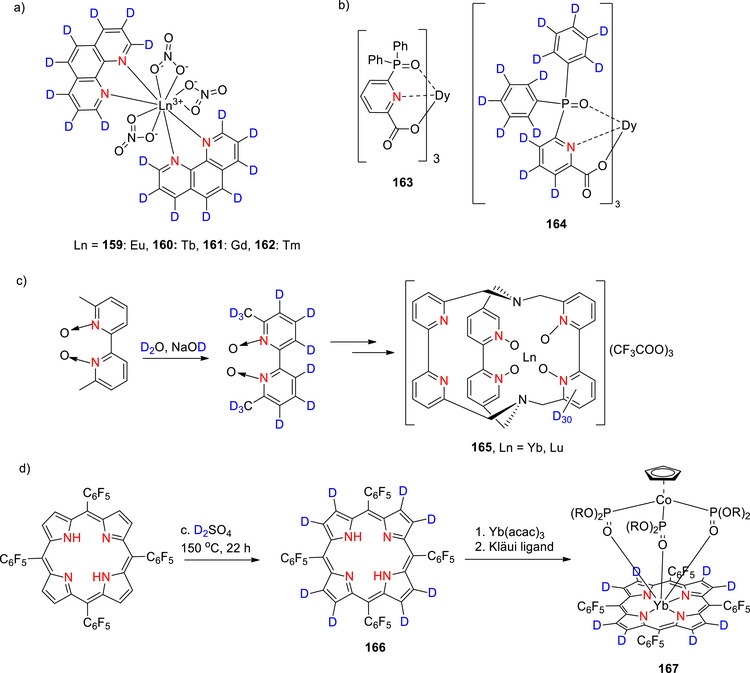
a) Ln complexes with perdeuterated phenanthroline (**159**–**162**).^[^ ^244^ ^]^ b) Chemical structures of Dy(III) complexes **163** and **164**. c) Synthesis of perdeuterated Yb cryptate **165**.^[^ ^274^ ^]^ d) Synthetic scheme to NIR‐emissive Ln complex **167**.^[^ ^275^, ^276^ ^]^

Luminescent lanthanide (Ln) complexes enable stable light‐emission and are therefore valuable in biomedical applications^[^ ^267^, ^268^ ^]^ and as sensors.^[^ ^269^, ^270^ ^]^ There are numerous reports involving the replacement of C─H with ‐D bonds in *N*‐heteroaromatic ligands either for enhancement of properties of these luminescent complexes or for simple curiosity studies. For example, the group of Hasegawa^[^ ^271^ ^]^ prepared phenanthroline (phen) and deuterated phenanthroline (phen‐d_8_) complexes of Eu, Tb, Gd and Tm (**159**–**162)**, respectively, Scheme 41a, and evaluated their structural and optoelectronic properties.

It was interesting to note that incorporation of the phen‐d_8_ influenced the various metal complexes differently. For example, luminescence was intensified for the Eu complex (**159**) compared with the nondeuterated analogue, while luminescence was the same for both the deuterated and nondeuterated versions of Tb and Tm complexes, **160** and **162**. The authors extended this study to a solvent effect to understand better the subtle differences between the complexes.^[^ ^272^ ^]^ Cai et al.^[^ ^273^ ^]^ reported enhanced luminescence (PLQY almost 5 times higher) of a perdeuterated Dy(III) complex **164** compared with its nondeuterated analogue, **163** (Scheme 41b) due to reduced quenching of luminescence of the Dy(III) complex by high‐energy O─H and C─H vibrations.

Seitz et al. has done much work to improve the brightness of NIR‐emissive Ln complex luminophores using perdeuteration.^[^ ^277^, ^278^, ^279^, ^280^, ^281^ ^]^ This strategy serves to suppresses multiphonon nonradiative deactivation pathways by removing high‐energy oscillators coupled with rigidification of the ligand architecture of the complexes.High photoluminescence quantum yields (PLQYs) could also be accomplished via dissolution of the complexes in deuterated solvents.

Most recently, Seik's group prepared a perdeuterated ytterbium cuprate complex **165** (Scheme 41c) capable of achieving the highest overall quantum yield reported for ytterbium complexes (12%).^[^ ^274^ ^]^ In this case, the deuteration served to remove the high‐energy oscillators (O─H and C─H stretching modes), therefore reducing nonradiative relaxation. Preparation of **165** commences with synthesis of perdeuterated bipyridyl ligands (bis(amine) and benzylic dibromide which both require bipyridyl b as the starting point (itself formed by H/D exchange, carried out with D_2_O and NaOD). Similarly, Hu et al.^[^ ^276^ ^]^ subsequently synthesized NIR‐emissive Ln complexes (**167**) consisting of Yb sandwiched between a perdeuterated porphyrinate antenna ligand and a deuterated Kläui ligand (Scheme 41d), commencing with the perdeuterated porphyrin **166** (itself prepared by heating the protonated substrate with sulfuric acid‐d_2_).^[^ ^275^ ^]^

**Scheme 42:**
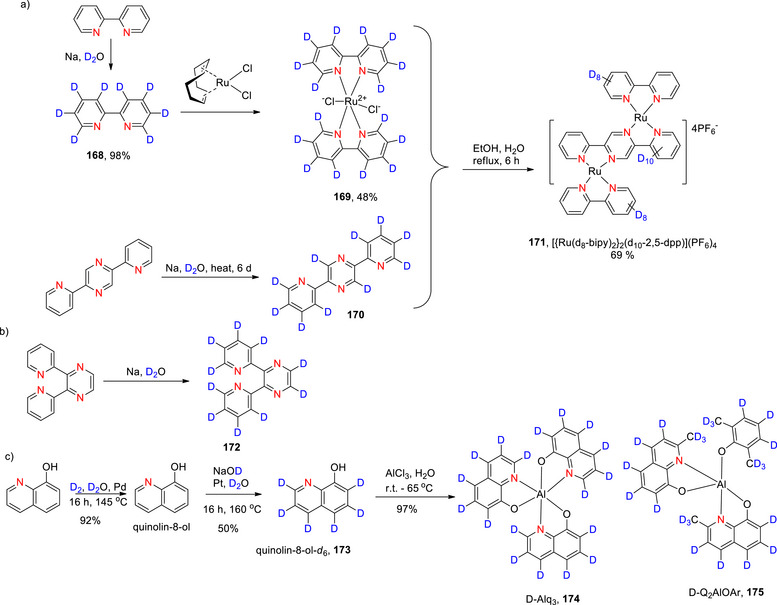
a). Synthesis of Ru‐complex containing deuterated ligands **171 (**from^[^ ^282^ ^]^) b) Synthesis of 5,6‐di(2‐pyridinyl‐3,4,5,6‐d_4_)‐pyrazine‐2,3‐d_2_ (**172**) (from^[^ ^133^ ^]^) c) Synthesis and chemical structures of Alq_3_‐d_18_ **174** and Q_2_AlOAr‐d_25_ **175**.^[^ ^283^ ^]^

Schulz et al.^[^ ^282^ ^]^ used selective deuteration and Raman spectroscopy to probe the ground and excited‐state electronic structures of homo‐ and heterobimetallic 2,5‐di(pyridin‐2‐yl) pyrazine ruthenium complexes **171** (Scheme 42a).

Scheme 42b shows the synthesis of bipyridyl‐*d*_8_, **172**, which can be prepared from its nonlabelled analogue by heating with Na metal and D_2_O,^[^ ^133^ ^]^ **172** was complexed with ruthenium and its photophysical properties were analyzed.

In a similar study, Wagner et al.^[^ ^284^ ^]^ used selective ligand deuteration to tune the photophysical properties of Ru(II) monometallic (**169)** Synthesis of these complexes requires prior synthesis or purchase of bipyridyl‐d_8_ and 5,6‐di(2‐pyridinyl‐3,4,5,6‐*d*_4_)‐pyrazine‐2,3‐d_2._^133]^

Similar complexes containing perdeuterated (and, in some cases, functionalized with other FGs) bipyridyl, phenanthroline, 2‐(3‐phenyl‐1*H*‐1,2,4‐triazol‐5‐yl)pyridine, and 4,7‐di(phenyl‐2,3,4,5,6‐d_5_)‐1,10‐phenanthroline‐d_x_ ligands (ligands deuterated according to Browne et al.^[^ ^133^ ^]^ have been prepared and their photophysical properties analyzed^[^ ^42^, ^285^, ^286^ ^]^).

##### Fluorescent OLED materials

Since the 1960s, it has been recognized that the fluorescence quantum yield (Φ_f_) (i.e., the ratio of photons absorbed to photons emitted through fluorescence) of the emission of deuterated aromatic chromophores is typically lower than (or the same as) their nonlabelled counterparts.^[^ ^287^ *^–^* ^290^ ^]^ However, when attached to the aliphatic portion, e.g., the *N*‐methyl groups of *N*‐alkyl auxochromes, the Φ_f_ is increased, mainly due to the inhibition of photochemically induced spectral shifts.^[^ ^291^, ^292^ ^]^ However, for electroluminescence (the emission of light caused by the interaction of a solid material with an electric field), it has been found that deuteration of the aromatic portion of select fluorophores can cause enhancement of light‐emitting efficiency and high‐voltage stability. For example, in a landmark paper published in 2007, Tong and Hwang^[^ ^283^ ^]^ demonstrated that electroluminescent devices fabricated from deuterated aluminum 8‐hydroxyquinoline (Alq_3_‐d_18_, **174**) and bis(2‐methyl‐8‐quinolinolato)(2,6‐dimethylphenolato)aluminum(III) (Q_2_AlOAr‐d_25_, **175**) (Scheme 42c) resulted in a 2.8‐fold increase in the external quantum efficiency (EQE) at 500 mA/cm^2^ in the case of **149** and 2.7‐fold increase at 150 mA/cm^2^ in the case of **150** in OLED devices, respectively, compared with nondeuterated devices.

**174** and **175** were prepared according to a procedure published by Keyes et al. for slightly different substrates^[^ ^293^ ^],^ i.e., the substrates were heated at 200 °C for 3 days in D_2_O (deuteration 99.9%) in the presence of Pd/C (10% Pd, Aldrich) (0.5 g) in a Teflon‐coated steel high‐pressure reactor. However, **175** can also be prepared by milder conditions, e.g., by reacting quinolin‐8‐ol‐d_6_ (prepared directly from quinolin‐8‐ol by one of two approaches in either 50%^[^ ^294^ ^]^ or 92%^[^ ^295^ ^]^ yield) with AlCl_3_ in water with heating.^[^ ^294^ ^]^ The synthesis of **173** has not been published, but conceivably commences with 2‐methyl‐quinolin‐8‐ol (CAS No. 826–81–3).

##### Phosphorescent Heavy‐Metal Complexes

Since the intersystem crossing (ISC) transition between states of different electronic spin multiplicity is nonradiative, only the transition of singlet excitons to the singlet ground state (S1→S0) is theoretically allowed for fluorescence. Therefore, only about 25% of singlet excitons can be harvested for luminescence and the upper limit of the EQE of OLEDs using conventional fluorescent emitters is 5%. To get access to the remaining 75% of excitons in triplet states, phosphorescent heavy‐metal complexes have been developed as emitters for the second generation of OLEDs. Indeed, replacement of H by D in aromatic compounds can result in increased phosphorescence lifetimes, due to a decreased rate for nonradiative deactivation of the lowest triplet state.^[^ ^289^, ^296^ *^–^* ^298^ ^]^

**Scheme 43:**
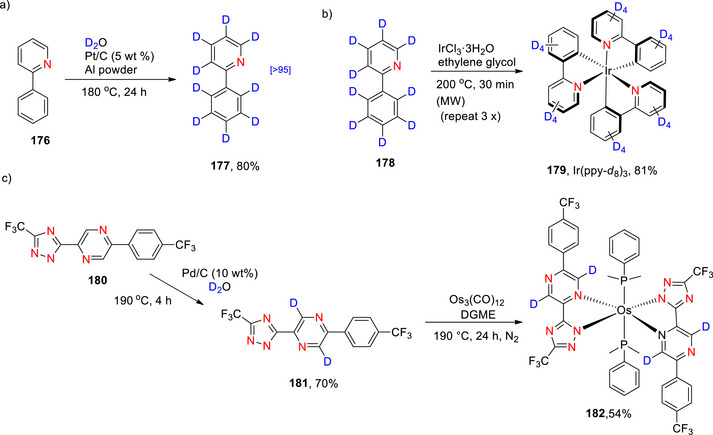
a) Synthesis of perdeuterated 2‐phenylpyridine, **177** b) Synthesis of *fac*‐tris(2‐phenylpyridinato) iridium (III) complex **179** ^[^ ^299^ ^]^ c) Synthesis of partially deuterated Os(II) complex **182** from ligand **180** (via dideuterated ligand **181**).

Abe et al.^[^ ^299^ ^]^ were the first to report the deuteration isotope effect on nonradiative transitions of *fac*‐tris(2‐phenylpyridinato) iridium (III) complexes (**179**) in solution (Scheme 43a), prepared using 2‐phenylpyridine **(176**) and perdeuterated 2‐phenylpyridine (**177**) in varying (and controlled) ratios.

Perdeuterated 2‐phenylpyridine **178** was prepared by heating 2‐phenylpyridine (**176**) with D_2_O in the presence of Pt/C and aluminium powder in a stainless reactor, enabling a high yield with an average > 95% D‐incorporation over the molecule (Scheme 43b). **178** could then be repeatedly heated with IrCl_3_.3H_2_O in ethylene glycol in a microwave to form complex **179**. In general, it was found that ligand deuteration increases the phosphorescence quantum yields (*Φ*_p_) and luminescence lifetimes (τ), e.g., Ir(ppy‐d_8_)_3_ has a Φ_p_ = 0.80 and τ = 2.1 µs (in MeCN) compared with normal complex Ir(ppy‐h_8_)_3_: Φ_p_ = 0.74 and τ = 1.8 µs.

In recent years, slightly different cyclometalated Ir(III) complexes have shown promise as photosensitizers in multicomponent systems for photocatalytic H_2_ generation from water.^[^ ^300^ ^]^ Of these complexes, Soman et al.^[^ ^301^ ^]^ prepared and evaluated deuterated [Ir(polypyridyl)_2_Cl_2_]PF_6_ complexes for isotope effects on their excited state properties. In addition to Ir complexes, NIR OLEDs have been fabricated using other deuterated heavy metal complexes. For example, Peng et al.^[^ ^302^ ^]^ formed the partially deuterated pyrazolyl azolate Os(II) complex **182** from ligand **181** which itself was prepared via late‐stage deuteration of nonlabelled ligand (**180**) (Scheme 43c).

**Scheme 44:**
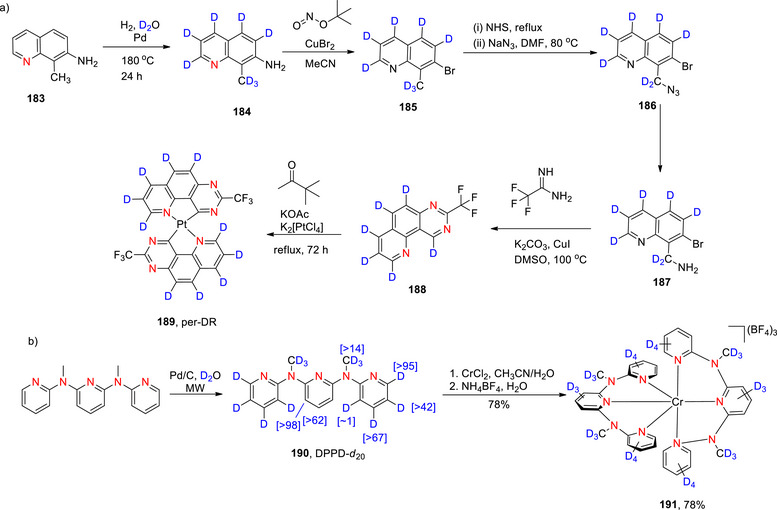
a) Synthesis of **189** from ligand **188** (commencing from **183** and via intermediate **184**–**187**) (from^[^ ^303^ ^]^) b) Synthesis of Cr‐complex **191** via perdeuterated ligand **190**.

Wang et al.^[^ ^303^ ^]^ prepared deuterated Pt(II) complexes as NIR emitters with emission quantum yields > 20%, enabling efficient NIR OLEDs. Synthesis of the complexes required initial preparation of the deuterated ligands which commenced with substrate **183**, transformed to intermediates (**184**–**189**) (Scheme 44a).

The researchers found that the deuterated complexes had higher PLQYs compared to their nondeuterated counterparts due to reduced reorganization energies and suppressed exciton–vibration coupling (and hence radiation‐less decay rates). In a subsequent study,^[^ ^304^ ^]^ the same authors prepared an additional Pt(II) complex **189**, commencing with preparation of perdeuterated quinoline substrate **183** and subsequent ligand **188**, (Scheme 44a), and found that its PLQY was 67% (EQE = 16.6%, radiance = compared with 50% for the nondeuterated analogue (λ_EL_ = 796 nm).

The use of heavy‐metal atoms such as Ir, Pt, and Os limits their further market expansion, considering current environmental concerns and cost fluctuations.^[^ ^305^ ^]^ One option is to use earth abundant metals such as Cr and Cu. An example of a deuterated phosphorescent Cr complex (**191**) (prepared from deuterated pyridine‐containing ligand **190** as shown in Scheme 44b was demonstrated by Wang et al.^[^ ^306^ ^]^ who prepared perdeuterated “molecular ruby” (octahedral Cr(III) complex) with PLQY = 30% for a 3d^n^ metal complex (n < 10) in solution at room temperature and a 2.3 ms lifetime (cf. the non deuterated equivalent which demonstrated a lifetime = 900 µs.^[^ ^307^ ^]^

#### 5.2.2 D‐HetAr in Understanding the Isotope Effect and Device Morphology in Organic Electronic Devices

##### Magneto‐Optoelectronic Responses

**Scheme 45:**
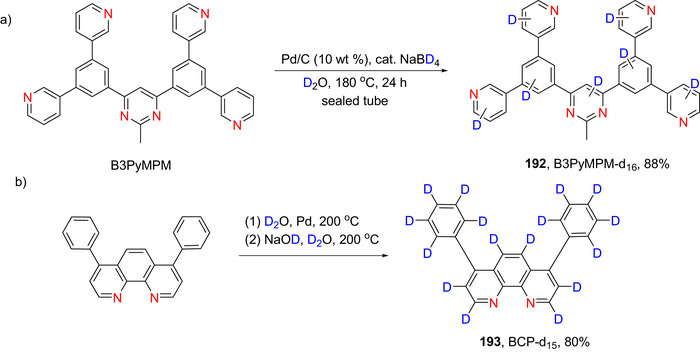
Synthesis of B3PyMPM‐*d*_16_ (**192**)^[^ ^310^ ^]^ and BCP‐*d*_15_ (**193**).^[^ ^311^ ^]^

The magneto‐optoelectronic responses of organic electronic devices, i.e., the change in EL intensity under the influence of an externally applied magnetic field is an ideal tool for studying certain processes, since it can be used to directly probe spin‐mixing mechanisms.^[^ ^308^, ^309^ ^]^ This can also be used to study the isotope effect in optoelectronics since protiums have a nuclear spin: I = ½ while D has spin I = 1). For example, Liu et al.^[^ ^310^ ^]^ investigated the magneto‐electroluminescence (MEL) response in TADF‐OLEDs as a function of the hyperfine field of a blend of perdeuterated donor mCP‐d_20_ and acceptor 4,6‐bis(3,5‐di‐3‐pyridylphenyl)‐2‐methylpyrimidine‐d_16_ (B3PyMPM‐d_16_) (**192**) (deuteration of B3PyMPM shown in Scheme 45a).

Although 26 Hs are available for H/D exchange on B3PyMPM, only 16 were exchanged (the authors did not specify which were exchanged (*personal communication*). Nevertheless, B3PyMPM was sufficiently deuterated to initiate a MEL response. In a similar way, the isotope effect was investigated for Alq_3_‐d_18_ (**174**) devices.^[^ ^312^, ^313^, ^314^ ^]^

##### Neutron Reflectometry Measurements

Deuterated materials are also useful in probing the properties of fabricated devices, e.g., interlayer diffusion and degradation, through neutron reflectometry (NR), wherein it is possible using deuterated compounds to obtain excellent contrast between different materials and organic layers, enabling better resolution within the samples and of the changes that occur.^[^ ^294^, ^315^ ^]^ One NR study involved the perdeuterated phenanthroline‐based compound electron and hole blocking layer material BCP‐d_15_ (**193**), which can be prepared in 80% yield from the unlabeled analogue under harsh conditions (Scheme 45b).^[^ ^311^ ^]^

### 5.3 Mechanistic Elucidation

As has already been mentioned, isotope labelling (and specifically, with D) is useful in the determination of organic and biochemical reaction mechanisms.^[^ ^316^ ^]^ This can be illustrated with two examples involving deuterated *N*‐heteroaromatic compounds.

In the first example,^[^ ^317^ ^]^ selective trideuteration of pyridines assists in understanding the mechanism of phototransposition (a type of photochemical reaction in which UV light induces the migration or rearrangement of atoms or groups within a molecule) in the vapor phase at 254 nm. Although initially hypothesized to occur through random scrambling, isotope labelling validated a well‐defined transposition mechanism (“each trideuteriopyridine isomer undergoes phototransposition to yield two isomeric trideuteriopyridine products”) involving photocyclization, nitrogen migration around the five sides of the cyclopentenyl ring, and rearomatization.

**Scheme 46:**
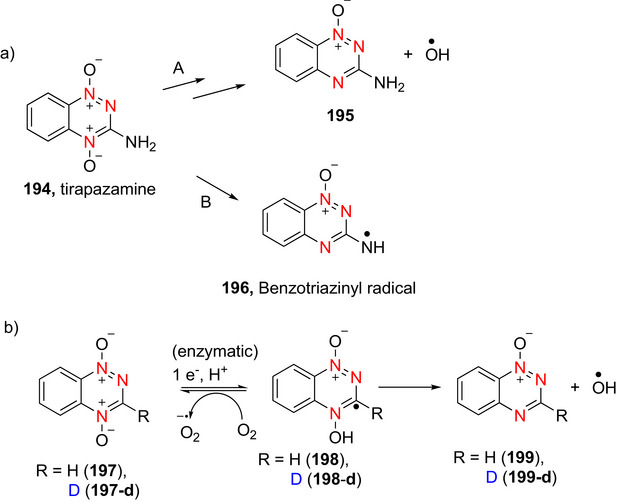
a) Two proposed pathways involving radical species for the action of the drug Tirapaazamine, **194**; b) Isotope labelling studies involving a comparison of reaction pathways for **197** versus **197‐d** helped elucidate the operative mechanistic pathway.

In the second example,^[^ ^318^ ^]^ we consider the mechanism of action of Tirapazamine (194), a drug used to selectively kill cancer cells via the formation of a reactive radical that can damage the DNA of cancer cells, especially under hypoxic conditions, resulting in the formation of the major drug metabolite, 194 (Scheme 46a).

However, the active radical species is not known, with two possible radicals (and their respective mechanisms of action) being debated: A) a hydroxy radical, formed alongside **195**, or B) a highly reactive benzotriazinyl radical **196** able to abstract hydrogen atoms from DNA. Tirapazamine analogues such as **197** (Scheme 32b) alternatively provide radical **198**. Deuterium–labeling of **198** (to form benzotriazine‐1,4‐di‐*N*‐oxide‐d_1_,198‐d) could also help in identifying the operative mechanistic pathway as follows: the proposed generation of the aryl radical **199** from **198** in the presence of a deuterium atom donor (MeOD‐d_4_) would produce the corresponding deuterated drug metabolite **199‐d**, whereas release of the hydroxyl radical from **198** would provide the nondeuterated metabolite **199**. The authors reported that the results of two different, but complementary, isotopic labeling studies provided evidence for a mechanism involving the release of the hydroxyl radical from the reductively activated drug intermediate **198**.

## 6 Concluding remarks and future perspectives

Due to their ubiquity and chemical properties, *N*‐heteroaromatics have found applications in a broad spectrum of fields including pharmaceutical drugs, agrochemicals, functional materials, dyes and imaging, and catalysis. Deuteration of molecules (including deuterated *N*‐heteroaromatic compounds) has played an important role in enhancing certain properties of molecules involved in these applications. Most commonly, this has taken the form of improved pharmacokinetic properties of pharmaceutical compounds, resulting in the approval to use select drugs containing deuterated *N*‐heteroaromatic components commercially (e.g., VX‐984, **14**, Figure 2). Deuterated N‐heteroaromatics have also been investigated in functional materials, e.g., OLEDs, dyes, catalysis. For example, partially deuterated Os(II) complex **182** demonstrated significantly higher PLQY compared with its nondeuterated counterpart due to suppressed stretching vibrations of C─D cf. C─H in the molecule, causing a decrease in the nonradiative transition processes (see Scheme 43).

In this paper, we have comprehensively reviewed synthetic approaches to D‐labelled 6NHetAr. We have identified that deuteration has not yet been demonstrated for certain *N*‐heteroaromatic compounds, namely: imidazole (although imidazole‐d_4_ is available commercially), pyrrolopyrimidine, phthalazines, and phenazines, all of which find utility in specific applications. In addition, deuterated analogues of cyclazine, pentazine, and heptazine^[^ ^319^ ^]^ might be useful for fundamental science investigation as well as for new functional materials in various applications.

Other opportunities include the following. There is a need for the development of new synthetic approaches to deuterated 6NHetAr that are more efficient and sustainable. This might include new metal‐free approaches such as organocatalysis or mechanochemistry, the use of biocatalysis, or translating from batch to continuous flow chemistry systems. For example, recent work involving regioselective functionalization via deprotonative metalation using Lithium Amide and Zinc Chloride Diamine Complex^[^ ^320^ ^]^ could be adapted to the TM‐free deuteration of 6NHetAr molecules. Similarly, skeletal editing to incorporate D into 6NHetAr compounds is yet to be demonstrated but is a promising approach for rapidly preparing these compounds from easily accessible substrates, as has been reported for the preparation of other scaffolds.^[^ ^321^, ^322^ ^]^TM‐free approaches such as the metalation procedure reported by Feng et al.^[^ ^320^ ^]^ might be fruitful in enabling selective deuteration on 6NHetAr.

Additionally, while improving the inherent stability (e.g., oxidative / photochemical) of compounds through the exchange of C─H for C─D bonds has been demonstrated for N‐heteroaromatic compounds in this review, but there remain many other opportunities outside of the applications discussed herein. For example, deuteration could be used to enhance the brightness and photochemical stability of organic fluorophores used in nonlinear optics and bioimaging^[^ ^323^, ^324^, ^325^ ^]^ and to increase the lifetime of catalysts featuring N‐heteroaromatic ligands that are prone to degradation. ^[^ ^326^, ^327^ ^]^ Similarly, as shown in section 5.2.1 selective D improves the PLQYs of N‐heteroaromatic containing light‐emissive compounds (through suppression of nonradiative decay processes); this could potentially also be demonstrated in novel Long‐Lived Room Temperature Phosphorescence (LL‐RTP)^[^ ^323^, ^328^ ^]^ and organic radical‐based^[^ ^329^, ^330^ ^]^ emitters (and other emitters) finding use in applications requiring long lifetimes, e.g., wearable and biomedical devices.

The future for deuterated 6NHetAr‐containing compounds is bright. We hope that this review inspires further research in this area and new technologies with more favorable properties because of D‐labelling. Since the review reveals the methods currently used for the preparation of deuterated heteroaromatics while highlighting gaps in the literature, we hope that it can be a useful resource for practitioners of heterocyclic chemistry, isotope labelling, green and sustainable chemistry, medicinal chemistry, and material sciences.

## Conflict of Interest

All the authors declare no conflict of interest.

## Data Availability

Data sharing not applicable—no new data generated.
